# Supplementary material for: Deimplementation strategy to reduce overtreatment of asymptomatic bacteriuria: a study protocol for a stepped-wedge cluster randomised trial
Source: BMJ Open. 2021 Feb 9;11(2):e039085. doi: 10.1136/bmjopen-2020-039085 (PMC7875305; doi:10.1136/bmjopen-2020-039085)
Supplement: Author's manuscript [file bmjopen-2020-039085.draft_revisions.pdf]

BMJ Open is committed to open peer review. As part of this commitment we make the peer review history of every article we publish publicly available.

When an article is published we post the peer reviewers' comments and the authors' responses online. We also post the versions of the paper that were used during peer review. These are the versions that the peer review comments apply to.

The versions of the paper that follow are the versions that were submitted during the peer review process. They are not the versions of record or the final published versions. They should not be cited or distributed as the published version of this manuscript.

BMJ Open is an open access journal and the full, final, typeset and author-corrected version of record of the manuscript is available on our site with no access controls, subscription charges or pay-per-view fees (http://bmjopen.bmj.com).

If you have any questions on BMJ Open's open peer review process please email info.bmjopen@bmj.com

BMJ Open

De-implementation strategy to reduce overtreatment of asymptomatic bacteriuria: a study protocol for a stepped wedge cluster randomized trial

Journal:	*BMJ Open*
Manuscript ID	bmjopen-2020-039085
Article Type:	Protocol
Date Submitted by the Author:	03-Apr-2020

Complete List of Authors:	van Horrik, Tessa; Amsterdam UMC - Locatie AMC, Internal Medicine, Infectious Diseases<br>Geerlings, Suzanne; Amsterdam UMC - Locatie AMC, Internal Medicine, Infectious Diseases<br>Stalenhoef, Janneke; OLVG Locatie Oost, Internal Medicine, Infectious Diseases<br>van Nieuwkoop, Cees; HagaZiekenhuis, Internal Medicine, Infectious Diseases<br>Saanen, Joppe; Amsterdam UMC - Locatie AMC, Emergency Medicine<br>Schneeberger, Caroline; Amsterdam UMC - Locatie AMC, Medical Microbiology<br>Laan, Bart; Amsterdam UMC - Locatie AMC, Internal Medicine, Infectious Diseases

Keywords:	Urinary tract infections < UROLOGY, INFECTIOUS DISEASES, Diagnostic microbiology < INFECTIOUS DISEASES

SCHOLARONE™
Manuscripts

I, the Submitting Author has the right to grant and does grant on behalf of all authors of the Work (as defined in the below author licence), an exclusive licence and/or a non-exclusive licence for contributions from authors who are: i) UK Crown employees; ii) where BMJ has agreed a CC-BY licence shall apply, and/or iii) in accordance with the terms applicable for US Federal Government officers or employees acting as part of their official duties; on a worldwide, perpetual, irrevocable, royalty-free basis to BMJ Publishing Group Ltd ("BMJ") its licensees and where the relevant Journal is co-owned by BMJ to the co-owners of the Journal, to publish the Work in this journal and any other BMJ products and to exploit all rights, as set out in our licence.

The Submitting Author accepts and understands that any supply made under these terms is made by BMJ to the Submitting Author unless you are acting as an employee on behalf of your employer or a postgraduate student of an affiliated institution which is paying any applicable article publishing charge ("APC") for Open Access articles. Where the Submitting Author wishes to make the Work available on an Open Access basis (and intends to pay the relevant APC), the terms of reuse of such Open Access shall be governed by a Creative Commons licence – details of these licences and which Creative Commons licence will apply to this Work are set out in our licence referred to above.

Other than as permitted in any relevant BMJ Author's Self Archiving Policies, I confirm this Work has not been accepted for publication elsewhere, is not being considered for publication elsewhere and does not duplicate material already published. I confirm all authors consent to publication of this Work and authorise the granting of this licence.

For peer review only

TITLE PAGE

Title: De-implementation strategy to reduce overtreatment of asymptomatic bacteriuria: a study protocol for a stepped wedge cluster randomized trial

Authors: T. M. Z. X. K. van Horrik[1], S. E. Geerlings[1], J. E. Stalenhoef[2], C. van Nieuwkoop[3], J. B. Saanen[4], C. Schneeberger-van der Linden[5], B. J. Laan[1]

[1]Amsterdam UMC, University of Amsterdam, Internal Medicine, Infectious Diseases, Amsterdam, The Netherlands

[2]OLVG, Internal Medicine, Infectious diseases, Amsterdam, The Netherlands

[3]Haga Teaching Hospital, Internal Medicine, Infectious Diseases, The Hague, The Netherlands

[4]Amsterdam UMC, University of Amsterdam, Emergency Medicine, Amsterdam, The Netherlands

[5]Amsterdam UMC, University of Amsterdam, Medical Microbiology, Amsterdam, The Netherlands

Correspondence to:

Tessa M. Z. X. K. van Horrik, Amsterdam UMC, University of Amsterdam, Internal Medicine, Infectious Diseases, Meibergdreef 9, Amsterdam, Netherlands, +31 20 5665992, t.m.vanhorrik@amsterdamumc.nl

Word count: 2654

For peer review only

ABSTRACT

Introduction Antimicrobials for asymptomatic bacteriuria is one of the most common unnecessary treatments. Earlier studies showed that this inappropriate treatment has a prevalence of 45%. With regard to international guidelines and antimicrobial stewardship, successful multifaceted interventions showed a decrease in overtreatment of ASB. The main objective of our study is to reduce overtreatment of ASB by 50% using a de-implementation strategy.

Methods and analysis We will use a stepped wedge cluster randomized design for our multifaceted de-implementation strategy, comparing outcomes before and after introduction of our intervention in the emergency department of five hospitals (clusters) in the Netherlands. The de-implementation strategy consists of a combination of interventions, such as education, audit and feedback. All patients (≥18 years old) who had a urine test while presented at the emergency department will be screened for eligibility. The primary endpoint is overtreatment of ASB without risk factors (e.g. pregnancy, planned invasive urologic procedures and neutropenia). Secondary endpoints are the duration of antimicrobial treatment for ASB, the number of urine cultures and urinalysis per 1000 patients, and overtreatment of positive urinalysis in asymptomatic patients.

Ethics and dissemination Our study aims to reduce inappropriate screening and treatment of ASB in emergency departments, improve healthcare quality, lower the increase in antimicrobial resistance, and save costs. If proven (cost)-effective, this study provides a well suited strategy for a nationwide approach to reduce overtreatment of ASB.

Ethical approval was obtained from Medical Ethics Research Committee of the Academic Medical Centre, with a waiver for informed consent. Local feasibility will be obtained by the local institutional review boards of all participating hospitals.

Registration details This trial is registered at Netherlands Trial Register, trial NL8242, trialregister.nl/trial/8242.

Keywords asymptomatic bacteriuria, urinary tract infections, antimicrobial stewardship, implementation science, unnecessary procedures.

For peer review only

STRENGTHS AND LIMITATIONS OF THIS STUDY

- We will use a stepped wedge cluster randomized design, which leads to introduction of our intervention in all participating hospitals.
- Our strategy is well suited for broad-scale de-implementation in emergency departments.
- A possible limitation of this study is that we are not able to evaluate the impact of an individual intervention, since we are using a multifaceted de-implementation strategy.

For peer review only

For peer review only - http://bmjopen.bmj.com/site/about/guidelines.xhtml

INTRODUCTION

To improve healthcare quality and save costs, practices with the aim to reduce unnecessary diagnostics, diagnostic error and related overtreatment, so called low-value care, have become more important. In the past years, international campaigns, such as Choosing Wisely and Do not Do prompts, have been launched to reduce unnecessary care, resulting in less risks for the patients, saving costs and giving room for valuable care.[1 2] In the Netherlands, the campaign ‘To Do or Not to Do?’ was introduced to de-implement unnecessary care.[3]

Antimicrobial resistance has become a worldwide problem to public health due to antibiotic overconsumption, and there is global consensus about reducing the use of antibiotics.[4] Urinary tract infection (UTI) is one of the most common infections for which antimicrobials are prescribed.[5] However, not all antimicrobial prescriptions are necessary. This is especially true in asymptomatic bacteriuria (ASB), which is the presence of bacteria in the urine of a patient, who does not have symptoms of a UTI. ASB is a common finding especially among women, elderly, and patients with urinary catheters. Guidelines strongly recommend not to screen for or treat ASB with antimicrobials, except for specific patients at risk of developing a complicated UTI.[5] This is also included in the American Choosing Wisely campaign, judging treatment of ASB as one of five low-value care services that should be avoided.[6] In 2015, this resulted in the recommendation: “Do not use antimicrobials to treat bacteriuria in older adults unless specific urinary tract symptoms are present”.[6]

Regardless, antimicrobials are still frequently used for patients with ASB. A systematic review and meta-analysis from 2017 showed a pooled prevalence of 45% among 4129 ASB cases (95% CI 39-50) who received inappropriate antimicrobial treatment.[7] The prevalence was even higher in a recent retrospective evaluation of 25 hospitals in the USA, in which 64% of the 2225 positive urine cultures were classified as ASB and 72% of 961 patients with ASB were treated with antimicrobials.[8] The exact use of antimicrobials for ASB in hospitals in the Netherlands is unknown. However, in 2015 a prospective study performed in ten nursing homes in the Netherlands showed that 115 (32%) of 356 residents with a possible UTI were treated inappropriately, and treatment of ASB was the most common reason.[9] These results indicate that reducing the treatment of ASB is an appropriate target of antimicrobial stewardship strategies.

Earlier studies showed effective multifaceted interventions to reduce overtreatment of ASB in nursing home residents and patients with urinary catheters. In 2005, a cluster randomized trial in 12 nursing homes showed a

For peer review only - http://bmjopen.bmj.com/site/about/guidelines.xhtml

reduction in antimicrobial use after a multifaceted implementation of a diagnostic and treatment algorithm (1.17 versus 1.59 courses of antimicrobials/1000 resident days).[10] For catheterized patients, a controlled before-after study in 2015 showed a decrease of urine culture ordering from 41.2 to 23.3 per 1000 bed-days.[11] Furthermore, short educational sessions combined with feedback in two academic tertiary acute care hospitals in 2015 resulted in a reduction of overtreatment for ASB of 8% (2 of 24 patients) in the intervention group compared to 52% (14 of 29 patients) in the control group (OR 0.1; 95% CI 0.02-0.49).[12] In 2018, these findings resulted in the development of an implementation guide to reduce overtreatment of ASB.[13]

However, effectiveness studies after the launch of this implementation guide are not yet performed in emergency departments. The results of earlier studies suggest that improving ASB management in the emergency department is necessary. In 2017, a prospective observational study performed in the emergency department of a tertiary care centre in the USA showed that 27 of 71 (38%) older adults were not correctly diagnosed with a UTI.[14] Furthermore, overtreatment of a suspected (but unconfirmed) UTI was very common in a retrospective study performed in the emergency department of another tertiary care centre in the USA in 2013, where 63 of 66 (95%) patients were treated while they had a negative urine culture.[15]

In this study, our aim is to reduce overtreatment of ASB by 50% by a multifaceted de-implementation strategy based on the implementation guide described above.

METHODS AND ANALYSIS

Study design and setting

We will use repeated measurement stepped wedge cluster randomized design. The de-implementation strategy will be introduced in the emergency department of five hospitals (clusters) in the Netherlands (one university and four general teaching hospitals) in six time periods consisting of one or two months, depending on how many patients will be included in the first month. Therefore, the total study duration is six to twelve months (Figure 1). The order in which the participating hospitals will receive the intervention will be randomly determined by an independent data manager. Every cluster will start in control condition and eventually every cluster will receive the intervention by the end of the trial. We planned to start our study on 1 May 2020. However, due to COVID-19 pandemic, the start of our study will be postponed until further notice.

For peer review only - http://bmjopen.bmj.com/site/about/guidelines.xhtml

Patient selection

We will screen all patients (≥ 18 years old) who had urine tests (culture and/or urinalysis), being performed while presented at the emergency department (Figure 2). First, we will exclude all negative urinalysis (defined as absence of leukocyte esterase and nitrite in dipsticks, and absence of leukocytes and bacteria in microscopic analysis) and negative urine cultures (Table 1). Further exclusion criteria are patients with a symptomatic UTI (systemic or local symptoms, according to the Centre for Disease Control's (CDC) National Healthcare Safety Network for Urinary Tract Infection) (Table 1),[16] active treatment for a UTI on presentation, patients with an alternate site of infection for which they receive antimicrobial treatment, and patients with ASB and risk factors, defined as pregnant women, patients prior to planned invasive urologic procedures associated with mucosal trauma (including transurethral surgery of the prostate or bladder, ureteroscopy including lithotripsy, and percutaneous stone surgery), and high-risk neutropenia (defined as absolute neutrophil count <500 cells/µl).

Table 1 Definitions of positive urinalysis, urinary tract infection and asymptomatic bacteriuria

Item	Definition
Positive urinalysis[17]	Urinalysis must have one or more of the following results:<br>Dipstick<br>• Abnormal nitrites<br>• Abnormal leukocyte esterase<br>Microscopic analysis:<br>• >5 leukocytes/high-powered field (or reference ranges according to local standard operating procedures)<br>• Abnormal amount of bacteria according local operating procedures.
Asymptomatic bacteriuria[5]	The presence of one or more species of bacteria growing in the urine at specified quantitative counts (≥10^5 CFU/ml) regardless of the presence of pyuria, without UTI symptoms or signs.
Urinary tract infection[16]	Patient has at least one of the following signs or symptoms: fever (>38.0°C), suprapubic tenderness†, costovertebral angle pain or tenderness†, urinary urgency, urinary frequency, or dysuria* AND

For peer review only

For peer review only - http://bmjopen.bmj.com/site/about/guidelines.xhtml

	patient has a urine culture with no more than two species of organisms identified, at least one of which is a bacterium of ≥10^5 CFU/ml.
Catheter-associated* urinary tract infection[16]	A urinary tract infection where a urinary catheter was in place for >2 days on the date of event, with day of placement being day 1, and was in place on the date of event or the day before.

†With no other recognized cause. *A urinary catheter in situ could cause patient complaints of frequency, urgency, or dysuria. Therefore, these symptoms cannot be used in patients with catheters.

Primary and secondary endpoints

The primary endpoint is the percentage of patients with ASB without risk factors or other alternative site of infections, who are treated with antimicrobials. We define ASB according to the international guideline provided by the Infectious Diseases Society of America (IDSA) (Table 1).[5] Secondary endpoints are the duration of antimicrobial treatment for ASB, the number of urine cultures and urinalysis (dipsticks and microscopic analysis) per 1000 patients at the emergency department, the percentage of asymptomatic patients treated for urine cultures that are in daily practice considered positive; a quantitative count of bacteria $\geq 10^3$ CFU/ml, and the percentage of patients without symptoms or risk factors treated with antimicrobials for a positive urinalysis without an obtained culture. In addition, the total number of urine cultures ordered in the hospital will be assessed explorative. Furthermore, the indications for the negative urine cultures will be assessed explorative during the baseline period.

Data collection

A list of all urine cultures and urinalyses performed in the emergency department will be composed of the microbiology laboratory and clinical chemistry data. All relevant patient information, including the presence of other possible infections, of those with positive cultures will be reviewed from medical and nursing records by a research physician to determine if patients have ASB. For patients with ASB, we will collect data on antimicrobial treatment, including antimicrobial prescription and duration of antimicrobial therapy, and the specified bacterial count according to the standard operating procedure of the microbiology laboratory. For feasibility reasons, a random sample of a total number of 220 negative urine cultures will be screened by medical record review to evaluate the indication for testing during the baseline period. We will use this data in the further development of the additional local part of the de-implementation strategy based on the assessment of

For peer review only - http://bmjopen.bmj.com/site/about/guidelines.xhtml

determinants of current practice. Furthermore, we will collect the patient level variables, including patient characteristics (age, gender, residency in nursing home), Charlson comorbidity index,[18] Modified Early Warning Score (MEWS),[19] chronic urinary catheter use, reason for presentation at the emergency department, and use of antimicrobials for uropathogens in the last seven days before presentation at the emergency department. Data from medical records will be collected in an electronic GCP-compliant database, and we will audit a random sample of 10% to assure the validity of data collection.

De-implementation strategy

Our de-implementation strategy is a tailored multifaceted intervention based on a Dutch de-implementation guide and an international implementation guide to reduce overtreatment of ASB in low-risk patients.[13 20] We will use a combination of interventions, such as education, audit and feedback and organizational adjustments, because these strategies have shown to be successful for reducing treatment of ASB in prior studies.[10 21] The de-implementation strategy can be divided into a general part that will be introduced in every hospital by our project group, and an additional local part by local healthcare workers, that will be focused on the local setting (Table 2).

For the general part of the strategy, we will start the intervention period with one or more educational meetings for all healthcare workers at the emergency department about overtreatment of ASB, where the results of the baseline period will be used as competitive feedback report between the participating hospitals. This competitive feedback will contribute to raise awareness.[22] During this meeting we will distribute posters and pocket cards with an algorithm (Figure 3a and 3b) for appropriate indications for urine cultures and urinalysis, and overtreatment of ASB, based on current guidelines.[5 23] These tools will be available throughout the intervention period and after the intervention period, if necessary. We will send emails provided with the anonymous feedback report and the algorithm to healthcare workers who were not able to join the meeting.

For the additional local part of the strategy, a local champion, one of the leading physicians, will be appointed in each participating hospital and will be responsible for the de-implementation strategy. The local champion will start an asymptomatic bacteriuria quality improvement team (ASB-team), which will consist of at least an emergency doctor, infectious disease physician, a resident internal medicine, and a nurse from the emergency department. The ASB-team will be motivated to assess barriers and facilitators within their hospital and to start

For peer review only - http://bmjopen.bmj.com/site/about/guidelines.xhtml

additional interventions based on the local setting. For example, by educating and/or reminding healthcare workers about appropriate urine testing and treatment for ASB, and evaluating and/or adjusting local protocols to reduce inappropriate urine testing and antimicrobial use. Further, the ASB-team will collaborate with the antibiotic stewardship teams. During the intervention period, the project coordinator will be in contact with the local champion and/or the ASB-team once every two weeks to exchange ideas and experiences based on interim results (ordering patterns of urine culture) and assessment of local facilitators and barriers.

Table 2 Summary of the de-implementation strategy

**Item**	**Description**
**General part**	
Educational meeting with competitive feedback	Combinations of strategies to reduce treatment of ASB.
Clinical decision tools	Pocket cards and posters with algorithms as an aid and reminder for appropriate and inappropriate urine testing and treatment for ASB.
**Local part**	
Local champion	One of the leading physicians who is responsible for the de-implementation strategy.
ASB-team	Consists of a local champion, emergency doctor, infectious disease physician, resident internal medicine, and nurse for the emergency department.
Additional interventions	Based on local setting. For example, education and/or reminding healthcare workers about appropriate urine testing and treatment of ASB.

Sample size

We will use a stepped wedge hospital-randomized design with six time periods and five steps, in which one hospital will switch from control to treatment at each step. Previous studies showed reduction of inappropriate treatment for ASB of approximately 50%.[7] Therefore, we anticipate that the proportion of patients overtreated for ASB will be reduced by 50% from 0.450 to 0.225 after the de-implementation strategy. Assuming an intra-hospital correlation coefficient of 0.10, a total of 420 patients (= 14 patients * 6 time periods * 5 hospitals) will achieve 80% power to detect this reduction using the two-sided Wald Z-Test with a significance level of 0.05.

For peer review only - http://bmjopen.bmj.com/site/about/guidelines.xhtml

We used PASS version 15.0.10 to calculate this sample size. During the same time period, we will included additional patients with positive urinalysis.

Statistical analysis

Effect evaluation

Categorical data were summarised as frequency and percentage, and continuous data as mean (standard deviation) or median (range). We will use fixed effects models to analyse the overall effect of our de-implementation strategy on overtreatment of ASB.[24] To adjust for clustering and temporal trends, we will include the clusters (hospitals) as a categorical predictor and the time in months as a fixed effect in the model. To correct for the Level 2 variance, we will multiply the standard error estimates by the DEFT, which is the square root of the design effect. We will evaluate the differences between baseline period and intervention period according an intention to treat principle. To adjust for confounders, we will include the main risk factors for overtreatment of ASB (namely age, acutely altered mental status, and positive urinalysis)[25] in the model. We will use the Akaike's Information Criterion and ANOVA tables to compare the fitting of the models. We will report the results of the adjusted, unadjusted, and intercept model. A two-sided p value < 0.05 was considered significant.

Process evaluation

During the planned regular meetings with members of the local ASB teams to report results and feedback, we will evaluate the introduced interventions. By exchanging thoughts and experiences on the implemented interventions, we will be able to determine what intervention(s) will work best in daily practice. For the process evaluation, we will evaluate the de-implementation strategy adherence and compliance. Therefore, all elements of the de-implementation strategy will be explored in all participating hospitals. We will award the hospitals with feedback points for all elements of the de-implementation strategy.[26] For example, points will be given for the number of persons present at the planned meetings and for every strategy that is implemented.

Economic evaluation

We will primarily perform a cost-effectiveness analysis, in which we evaluate the cost effectiveness ratio (CER) (CER = cost of de-implementation / healthcare cost reduction). Our goal is to achieve a reduction in urine culture, urinalysis, and antimicrobial prescriptions, and thereby cost saving. The costs of our de-implementation

For peer review only - http://bmjopen.bmj.com/site/about/guidelines.xhtml

strategy will be divided in non-recurrent study-related costs and recurrent de-implementation-related costs. Non-recurrent study-related costs include material costs, developmental costs of the de-implementation strategy, and costs of evaluation of the de-implementation. Recurrent costs include the costs to introduce the de-implementation strategy. For feasibility reasons, we will use the number of all urine cultures, urinalyses and antimicrobial prescriptions, representing the main cost benefits due to our strategy. The costs for urine cultures, urinalysis, and antimicrobials used in the analyses will be based on Dutch guideline on healthcare economic evaluations.[27] The result of the cost-effectiveness analysis will be used in a budget impact analysis to assess the financial impact on the hospital's budget to implement and sustain the de-implementation strategy.

Ethical approval

Ethical approval was obtained on 13 December 2019 from Medical Ethics Research Committee of the Academic Medical Centre, with a waiver for informed consent. Local feasibility will be obtained by the local institutional scientific review boards of all participated hospitals. This trial is registered at Netherlands Trial Register, trial NL8242, trialregister.nl/trial/8242.

Patient and Public Involvement

This research will be performed without patient involvement. Patients will not be invited to comment on the study design and will not be consulted to develop patient relevant outcomes or interpret the results. Patients will not be invited to contribute to the writing or editing of this document for readability or accuracy.

DISCUSSION

This study protocol describes the design, de-implementation strategy and process evaluation to reduce overtreatment of ASB in daily practice of emergency medicine. Our de-implementation strategy could prevent the use of unnecessary urine diagnostics and subsequent inappropriate treatment of ASB, improve healthcare quality, lower the increase in antimicrobial resistance, and save costs. If proven (cost) effective, this study provides a strategy for a nationwide approach to reduce overtreatment of ASB.

In our study, we will not evaluate the clinical outcome of our strategy, including potential clinical benefits, such as less side effects of antibiotics, since previous studies already showed that ASB in non-risk patients does not lead to increased risk of symptomatic UTI and non-treatment of ASB is safe.[28 29]

For peer review only - http://bmjopen.bmj.com/site/about/guidelines.xhtml

By using a stepped wedge cluster randomized design, we will be able to compare the outcomes within a cluster between the time intervals in which a cluster received the control and the interventions. Furthermore, our intervention will be enrolled in a systematic manner and eventually be introduced in all participating hospitals. A limitation of stepped wedge designs is that this require larger sample sizes, as patients' characteristics may be similar within one cluster. By randomization of the start time of the clusters, we intend to balance these important characteristics. However, this cannot be ensured with our relatively small number of clusters.[30] Another limitation is that we cannot evaluate the impact of an individual intervention. However, we expect that our de-implementation strategy is low cost and well suitable for broad scale implementation.

For peer review only

REFERENCES

1. The National Institute for Health and Care Excellence (NICE). NICE Do not Do prompts 2013 [Available from: https://www.nice.org.uk/ sharedlearning/nice-do-not-do-prompts accessed 09-09-2019.

2. American Board of Internal Medicine Foundation. Choosing Wisely [Available from: http://www.choosingwisely.org/ accessed 09-09-2019.

3. Nederlandse Federatie van UMC. Doen of laten? [Available from: https://www.doenoflaten.nl/ accessed 09-09-2019.

4. World Health Organization. Global action plan on antimicrobial resistance. Geneva2015 [Available from: https://apps.who.int/iris/bitstream/handle/10665/193736/9789241509763_eng.pdf?sequence=1 accessed 10-10-2019.

5. Nicolle LE, Gupta K, Bradley SF, et al. Clinical Practice Guideline for the Management of Asymptomatic Bacteriuria: 2019 Update by the Infectious Diseases Society of Americaa. *Clinical infectious diseases : an official publication of the Infectious Diseases Society of America* 2019 doi: 10.1093/cid/ciy1121 [published Online First: 2019/03/22]

6. Choosing Wisely: an initiative of the ABIM Foundation. Ten things physicians and patients should question 2013 [Available from: https://www.choosingwisely.org/doctor-patient-lists/american-geriatrics-society/ accessed 09-09-2019.

7. Flokas ME, Andreatos N, Alevizakos M, et al. Inappropriate Management of Asymptomatic Patients With Positive Urine Cultures: A Systematic Review and Meta-analysis. *Open forum infectious diseases* 2017;4(4):ofx207. doi: 10.1093/ofid/ofx207 [published Online First: 2017/12/12]

8. Spivak ES, Burk M, Zhang R, et al. Management of Bacteriuria in Veterans Affairs Hospitals. *Clinical infectious diseases : an official publication of the Infectious Diseases Society of America* 2017;65(6):910-17. doi: 10.1093/cid/cix474 [published Online First: 2017/05/23]

9. van Buul LW, Veenhuizen RB, Achterberg WP, et al. Antibiotic prescribing in Dutch nursing homes: how appropriate is it? *J Am Med Dir Assoc* 2015;16(3):229-37. doi: 10.1016/j.jamda.2014.10.003 [published Online First: 2014/12/03]

10. Loeb M, Brazil K, Lohfeld L, et al. Effect of a multifaceted intervention on number of antimicrobial prescriptions for suspected urinary tract infections in residents of nursing homes: cluster randomised controlled trial. *BMJ (Clinical research ed)* 2005;331(7518):669. doi: 10.1136/bmj.38602.586343.55 [published Online First: 2005/09/10]

For peer review only - http://bmjopen.bmj.com/site/about/guidelines.xhtml

11. Trautner BW, Grigoryan L, Petersen NJ, et al. Effectiveness of an Antimicrobial Stewardship Approach for Urinary Catheter-Associated Asymptomatic Bacteriuria. *JAMA internal medicine* 2015;175(7):1120-7. doi: 10.1001/jamainternmed.2015.1878 [published Online First: 2015/05/27]

12. Irfan N, Brooks A, Mithoowani S, et al. A Controlled Quasi-Experimental Study of an Educational Intervention to Reduce the Unnecessary Use of Antimicrobials For Asymptomatic Bacteriuria. *PloS one* 2015;10(7):e0132071. doi: 10.1371/journal.pone.0132071 [published Online First: 2015/07/17]

13. Daniel M, Keller S, Mozafarihashjin M, et al. An Implementation Guide to Reducing Overtreatment of Asymptomatic Bacteriuria. *JAMA internal medicine* 2018;178(2):271-76. doi: 10.1001/jamainternmed.2017.7290 [published Online First: 2017/12/12]

14. Caterino JM, Leininger R, Kline DM, et al. Accuracy of Current Diagnostic Criteria for Acute Bacterial Infection in Older Adults in the Emergency Department. *Journal of the American Geriatrics Society* 2017;65(8):1802-09. doi: 10.1111/jgs.14912 [published Online First: 2017/04/26]

15. Gordon LB, Waxman MJ, Ragsdale L, et al. Overtreatment of presumed urinary tract infection in older women presenting to the emergency department. *Journal of the American Geriatrics Society* 2013;61(5):788-92. doi: 10.1111/jgs.12203 [published Online First: 2013/04/18]

16. CDC National Healthcare Safety Network (NHSN). Patient Safety Component Manual 2019 [Available from: https://www.cdc.gov/nhsn/pdfs/pscmanual/pcsmanual_current.pdf accessed 26-09-2019.

17. Deville WL, Yzermans JC, van Duijn NP, et al. The urine dipstick test useful to rule out infections. A meta-analysis of the accuracy. *BMC urology* 2004;4:4. doi: 10.1186/1471-2490-4-4 [published Online First: 2004/06/04]

18. Quan H, Li B, Couris CM, et al. Updating and validating the Charlson comorbidity index and score for risk adjustment in hospital discharge abstracts using data from 6 countries. *American journal of epidemiology* 2011;173(6):676-82. doi: 10.1093/aje/kwq433 [published Online First: 2011/02/19]

19. Subbe CP, Kruger M, Rutherford P, et al. Validation of a modified Early Warning Score in medical admissions. *QJM* 2001;94(10):521-6. doi: 10.1093/qjmed/94.10.521 [published Online First: 2001/10/06]

20. van Dulmen S, Kool T, Verkerk E. Deïmplementatiegids 2019 [Available from: https://www.doenoflaten.nl/pdf/deïmplementatiegids_jan_2019.pdf accessed 10-10-2019.

For peer review only - http://bmjopen.bmj.com/site/about/guidelines.xhtml

21. McMaughan DK, Nwaiwu O, Zhao H, et al. Impact of a decision-making aid for suspected urinary tract infections on antibiotic overuse in nursing homes. *BMC geriatrics* 2016;16:81. doi: 10.1186/s12877-016-0255-9 [published Online First: 2016/04/17]

22. Spoorenberg V, Hulscher ME, Geskus RB, et al. A Cluster-Randomized Trial of Two Strategies to Improve Antibiotic Use for Patients with a Complicated Urinary Tract Infection. *PloS one* 2015;10(12):e0142672. doi: 10.1371/journal.pone.0142672 [published Online First: 2015/12/05]

23. Verenso. Urineweginfecties bij kwetsbare ouderen 2018 [Available from: https://www.verenso.nl/richtlijnen-en-praktijkvoering/richtlijnendatabase/urineweginfecties accessed 11-10-2019.

24. McNeish D, Stapleton LM. Modeling Clustered Data with Very Few Clusters. *Multivariate Behav Res* 2016;51(4):495-518. doi: 10.1080/00273171.2016.1167008 [published Online First: 2016/06/09]

25. Petty LA, Vaughn VM, Flanders SA, et al. Risk Factors and Outcomes Associated With Treatment of Asymptomatic Bacteriuria in Hospitalized Patients. *JAMA internal medicine* 2019 doi: 10.1001/jamainternmed.2019.2871 [published Online First: 2019/08/27]

26. Laan BJ, Maaskant JM, Spijkerman IJB, et al. De-implementation strategy to reduce inappropriate use of intravenous and urinary catheters (RICAT): a multicentre, prospective, interrupted time-series and before and after study. *The Lancet Infectious diseases* 2020 doi: 10.1016/S1473-3099(19)30709-1 [published Online First: 2020/03/11]

27. Hakkaart-van Roijen L, van der Linden N, Bouwmans C, et al. Kostenhandleiding: Methodologie van kostenonderzoek en referentieprijzen voor economische evaluaties in de gezondheidszorg 2016 [Available from: https://www.zorginstituutnederland.nl/publicaties/publicatie/2016/02/29/richtlijn-voor-het-uitvoeren-van-economische-evaluaties-in-de-gezondheidszorg accessed 27-09-2019.

28. Harding GK, Zhanel GG, Nicolle LE, et al. Antimicrobial treatment in diabetic women with asymptomatic bacteriuria. *The New England journal of medicine* 2002;347(20):1576-83. doi: 10.1056/NEJMoa021042 [published Online First: 2002/11/15]

29. Bengtsson C, Bengtsson U, Bjorkelund C, et al. Bacteriuria in a population sample of women: 24-year follow-up study. Results from the prospective population-based study of women in Gothenburg, Sweden. *Scandinavian journal of urology and nephrology* 1998;32(4):284-9. [published Online First: 1998/10/09]

30. Ellenberg SS. The Stepped-Wedge Clinical Trial: Evaluation by Rolling Deployment. *Jama* 2018;319(6):607-08. doi: 10.1001/jama.2017.21993 [published Online First: 2018/02/17]

For peer review only

For peer review only

For peer review only - http://bmjopen.bmj.com/site/about/guidelines.xhtml

AUTHORS' CONTRIBUTIONS

BJL and SEG were involved in the conception of the study and coordinated the funding application. TVH, SEG and BJL drafted the manuscript. BJL outlined the statistical analysis with support of the Clinical Research Unit of the Academic Medical Centre. All authors read and approved the final version of the manuscript.

FUNDING STATEMENT

This study is supported by the Netherlands Organisation for Health Research and Development (ZonMw) grant number 839205002. They will have no role in the study design or analysis.

COMPETING INTERESTS STATEMENT

The authors declare that they have no competing interests.

FIGURES

Figure 1 The stepped wedge design for de-implementation strategy.

Figure 2 Study profile. ASB=Asymptomatic bacteriuria. UTI=Urinary tract infection.

*Risk factors are pregnancy, patients prior to planned urologic procedures associated with mucosal trauma, and high-risk neutropenia.

Figure 3a Algorithm of indications for urinalysis and cultures in symptomatic patients.

ASB=Asymptomatic bacteriuria. UTI=Urinary tract infection. SWAB=Stichting Werkgroep Antibiotica Beleid (the Dutch Working Party on Antibiotic Policy).

Figure 3b Algorithm of indications for urinalysis and cultures in asymptomatic patients.

ASB=Asymptomatic bacteriuria. UTI=Urinary tract infection. SWAB=Stichting Werkgroep Antibiotica Beleid (the Dutch Working Party on Antibiotic Policy).

*Fever (>38°C), painful urination, urinary urgency, frequent miction, suprapubic pain, flank pain.

For peer review only

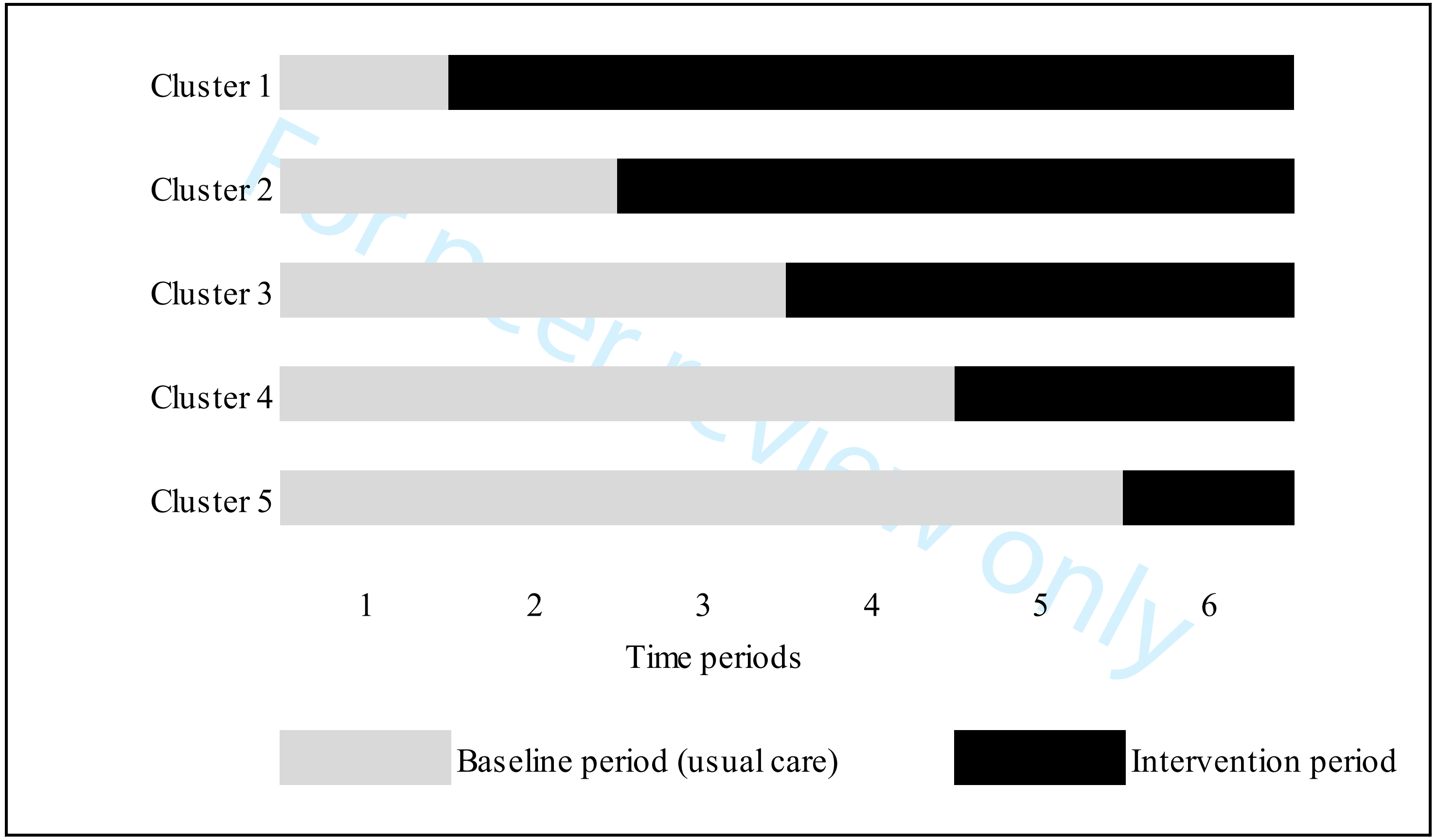
Cluster 1
Cluster 2
Cluster 3
Cluster 4
Cluster 5
1
2
3
4
5
6
Time periods
Baseline period (usual care)
Intervention period

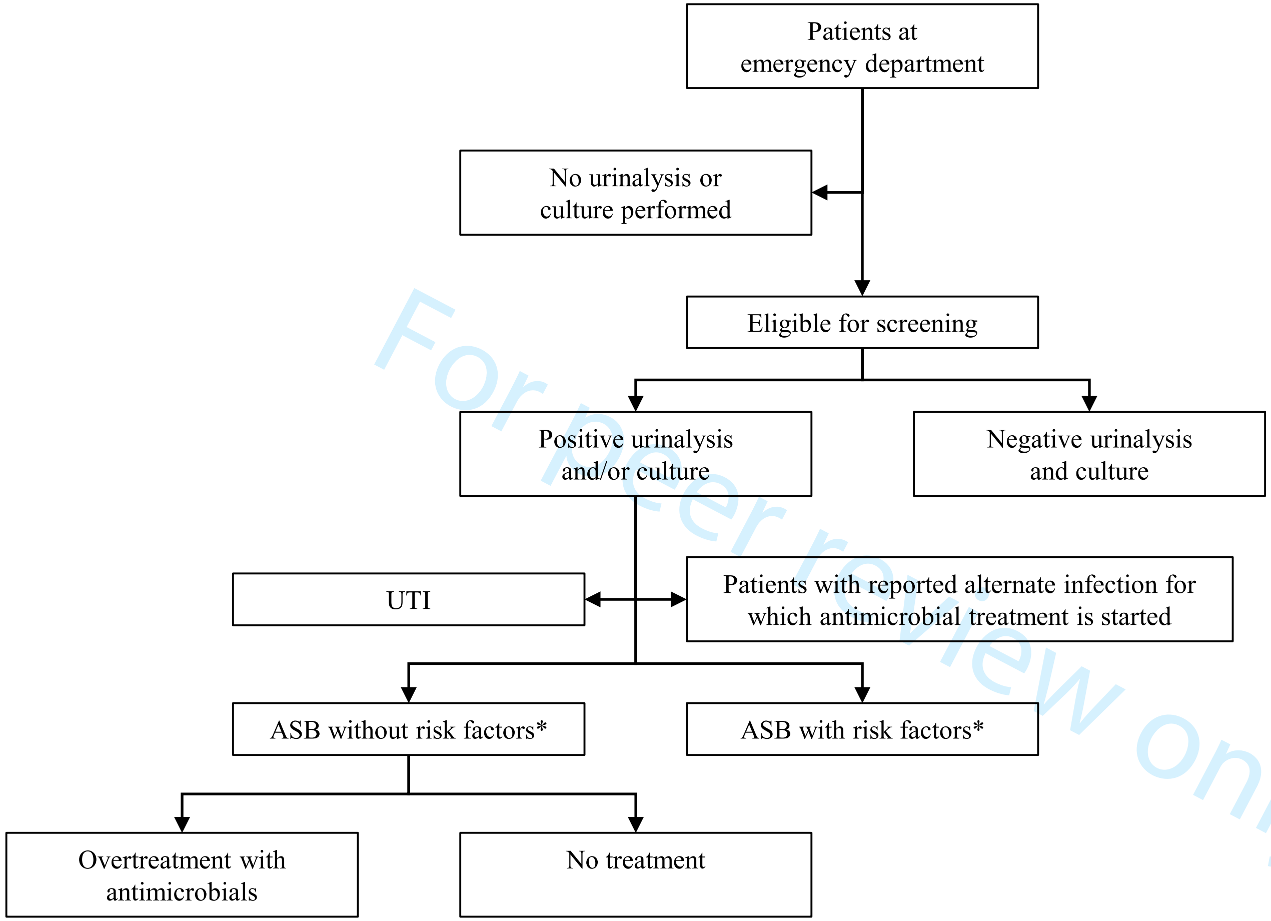
Patients at emergency department
No urinalysis or culture performed
Eligible for screening
Positive urinalysis and/or culture
Negative urinalysis and culture
UTI
Patients with reported alternate infection for which antimicrobial treatment is started
ASB without risk factors*
ASB with risk factors*
Overtreatment with antimicrobials
No treatment

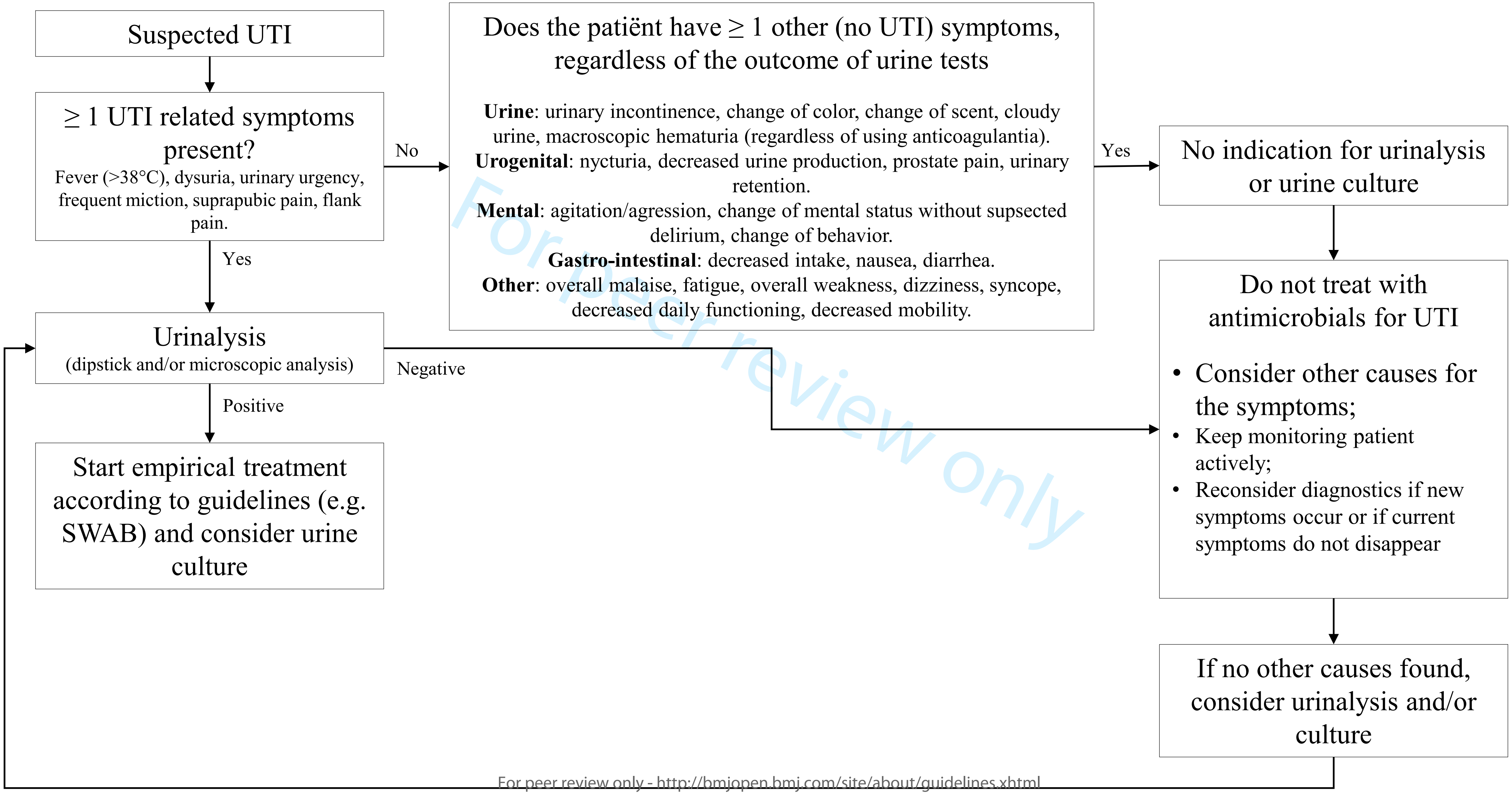

For peer review only

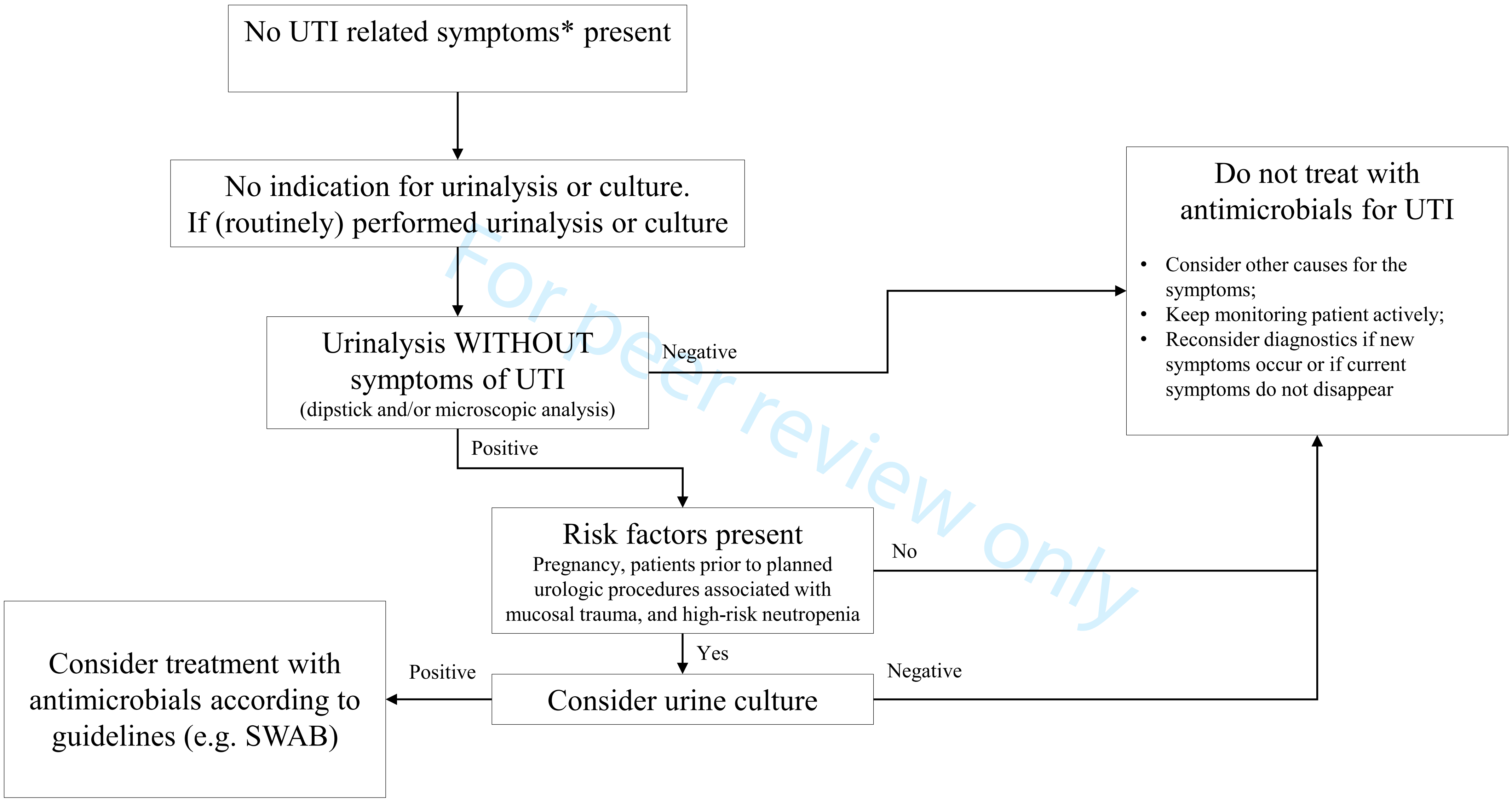
No UTI related symptoms* present
No indication for urinalysis or culture.
If (routinely) performed urinalysis or culture
Urinalysis WITHOUT symptoms of UTI
(dipstick and/or microscopic analysis)
Negative
Positive
Do not treat with antimicrobials for UTI
• Consider other causes for the symptoms;
• Keep monitoring patient actively;
• Reconsider diagnostics if new symptoms occur or if current symptoms do not disappear
Risk factors present
Pregnancy, patients prior to planned urologic procedures associated with mucosal trauma, and high-risk neutropenia
No
Yes
Consider urine culture
Positive
Negative
Consider treatment with antimicrobials according to guidelines (e.g. SWAB)

Reporting checklist for protocol of a clinical trial.

Based on the SPIRIT guidelines.

Instructions to authors

Complete this checklist by entering the page numbers from your manuscript where readers will find each of the items listed below.

Your article may not currently address all the items on the checklist. Please modify your text to include the missing information. If you are certain that an item does not apply, please write "n/a" and provide a short explanation.

Upload your completed checklist as an extra file when you submit to a journal.

In your methods section, say that you used the SPIRITreporting guidelines, and cite them as:

Chan A-W, Tetzlaff JM, Altman DG, Laupacis A, Gøtzsche PC, Krleža-Jerić K, Hróbjartsson A, Mann H, Dickersin K, Berlin J, Doré C, Parulekar W, Summerskill W, Groves T, Schulz K, Sox H, Rockhold FW, Rennie D, Moher D. SPIRIT 2013 Statement: Defining standard protocol items for clinical trials. Ann Intern Med. 2013;158(3):200-207

		Reporting Item	Page Number
**Administrative information**			
Title	#1	Descriptive title identifying the study design, population, interventions, and, if applicable, trial acronym	1
Trial registration	#2a	Trial identifier and registry name. If not yet registered, name of intended registry	11
Trial registration: data set	#2b	All items from the World Health Organization Trial Registration Data Set	11
Protocol version	#3	Date and version identifier	1
Funding	#4	Sources and types of financial, material, and other support	17
Roles and responsibilities: contributorship	#5a	Names, affiliations, and roles of protocol contributors	17

For peer review only

Roles and responsibilities: sponsor contact information	#5b	Name and contact information for the trial sponsor	17
Roles and responsibilities: sponsor and funder	#5c	Role of study sponsor and funders, if any, in study design; collection, management, analysis, and interpretation of data; writing of the report; and the decision to submit the report for publication, including whether they will have ultimate authority over any of these activities	17
Roles and responsibilities: committees	#5d	Composition, roles, and responsibilities of the coordinating centre, steering committee, endpoint adjudication committee, data management team, and other individuals or groups overseeing the trial, if applicable (see Item 21a for data monitoring committee)	17
**Introduction**			
Background and rationale	#6a	Description of research question and justification for undertaking the trial, including summary of relevant studies (published and unpublished) examining benefits and harms for each intervention	4
Background and rationale: choice of comparators	#6b	Explanation for choice of comparators	4
Objectives	#7	Specific objectives or hypotheses	4
Trial design	#8	Description of trial design including type of trial (eg, parallel group, crossover, factorial, single group), allocation ratio, and framework (eg, superiority, equivalence, non-inferiority, exploratory)	5
**Methods: Participants, interventions, and outcomes**			
Study setting	#9	Description of study settings (eg, community clinic, academic hospital) and list of countries where data will be collected. Reference to where list of study sites can be obtained	5
Eligibility criteria	#10	Inclusion and exclusion criteria for participants. If applicable, eligibility criteria for study centres and individuals who will	6

For peer review only

		perform the interventions (eg, surgeons, psychotherapists)	
Interventions: description	#11a	Interventions for each group with sufficient detail to allow replication, including how and when they will be administered	8
Interventions: modifications	#11b	Criteria for discontinuing or modifying allocated interventions for a given trial participant (eg, drug dose change in response to harms, participant request, or improving / worsening disease)	8
Interventions: adherance	#11c	Strategies to improve adherence to intervention protocols, and any procedures for monitoring adherence (eg, drug tablet return; laboratory tests)	9
Interventions: concomitant care	#11d	Relevant concomitant care and interventions that are permitted or prohibited during the trial	9
Outcomes	#12	Primary, secondary, and other outcomes, including the specific measurement variable (eg, systolic blood pressure), analysis metric (eg, change from baseline, final value, time to event), method of aggregation (eg, median, proportion), and time point for each outcome. Explanation of the clinical relevance of chosen efficacy and harm outcomes is strongly recommended	7
Participant timeline	#13	Time schedule of enrolment, interventions (including any run-ins and washouts), assessments, and visits for participants. A schematic diagram is highly recommended (see Figure)	5
Sample size	#14	Estimated number of participants needed to achieve study objectives and how it was determined, including clinical and statistical assumptions supporting any sample size calculations	9
Recruitment	#15	Strategies for achieving adequate participant enrolment to reach target sample size	6
**Methods: Assignment of interventions (for controlled trials)**			
Allocation: sequence generation	#16a	Method of generating the allocation sequence (eg, computer-generated random numbers), and list of any factors for stratification. To reduce predictability of a random sequence, details of any planned restriction (eg, blocking) should be provided in a separate document that is unavailable to those who enrol participants or assign interventions	5

For peer review only

Allocation concealment mechanism	#16b	Mechanism of implementing the allocation sequence (eg, central telephone; sequentially numbered, opaque, sealed envelopes), describing any steps to conceal the sequence until interventions are assigned	5
Allocation: implementation	#16c	Who will generate the allocation sequence, who will enrol participants, and who will assign participants to interventions	5
Blinding (masking)	#17a	Who will be blinded after assignment to interventions (eg, trial participants, care providers, outcome assessors, data analysts), and how	n/a
Blinding (masking): emergency unblinding	#17b	If blinded, circumstances under which unblinding is permissible, and procedure for revealing a participant's allocated intervention during the trial	n/a
**Methods: Data collection, management, and analysis**			
Data collection plan	#18a	Plans for assessment and collection of outcome, baseline, and other trial data, including any related processes to promote data quality (eg, duplicate measurements, training of assessors) and a description of study instruments (eg, questionnaires, laboratory tests) along with their reliability and validity, if known. Reference to where data collection forms can be found, if not in the protocol	7
Data collection plan: retention	#18b	Plans to promote participant retention and complete follow-up, including list of any outcome data to be collected for participants who discontinue or deviate from intervention protocols	7
Data management	#19	Plans for data entry, coding, security, and storage, including any related processes to promote data quality (eg, double data entry; range checks for data values). Reference to where details of data management procedures can be found, if not in the protocol	8
Statistics: outcomes	#20a	Statistical methods for analysing primary and secondary outcomes. Reference to where other details of the statistical analysis plan can be found, if not in the protocol	10
Statistics: additional analyses	#20b	Methods for any additional analyses (eg, subgroup and adjusted analyses)	10

For peer review only

Statistics: analysis population and missing data	#20c	Definition of analysis population relating to protocol non-adherence (eg, as randomised analysis), and any statistical methods to handle missing data (eg, multiple imputation)	10
**Methods: Monitoring**			
Data monitoring: formal committee	#21a	Composition of data monitoring committee (DMC); summary of its role and reporting structure; statement of whether it is independent from the sponsor and competing interests; and reference to where further details about its charter can be found, if not in the protocol. Alternatively, an explanation of why a DMC is not needed	n/a
Data monitoring: interim analysis	#21b	Description of any interim analyses and stopping guidelines, including who will have access to these interim results and make the final decision to terminate the trial	n/a
Harms	#22	Plans for collecting, assessing, reporting, and managing solicited and spontaneously reported adverse events and other unintended effects of trial interventions or trial conduct	11
Auditing	#23	Frequency and procedures for auditing trial conduct, if any, and whether the process will be independent from investigators and the sponsor	11
**Ethics and dissemination**			
Research ethics approval	#24	Plans for seeking research ethics committee / institutional review board (REC / IRB) approval	11
Protocol amendments	#25	Plans for communicating important protocol modifications (eg, changes to eligibility criteria, outcomes, analyses) to relevant parties (eg, investigators, REC / IRBs, trial participants, trial registries, journals, regulators)	n/a
Consent or assent	#26a	Who will obtain informed consent or assent from potential trial participants or authorised surrogates, and how (see Item 32)	11
Consent or assent: ancillary studies	#26b	Additional consent provisions for collection and use of participant data and biological specimens in ancillary studies, if applicable	11
Confidentiality	#27	How personal information about potential and enrolled participants will be collected, shared, and maintained in order to protect confidentiality before, during, and after the trial	11

For peer review only

Declaration of interests	#28	Financial and other competing interests for principal investigators for the overall trial and each study site	17
Data access	#29	Statement of who will have access to the final trial dataset, and disclosure of contractual agreements that limit such access for investigators	11
Ancillary and post trial care	#30	Provisions, if any, for ancillary and post-trial care, and for compensation to those who suffer harm from trial participation	n/a
Dissemination policy: trial results	#31a	Plans for investigators and sponsor to communicate trial results to participants, healthcare professionals, the public, and other relevant groups (eg, via publication, reporting in results databases, or other data sharing arrangements), including any publication restrictions	11
Dissemination policy: authorship	#31b	Authorship eligibility guidelines and any intended use of professional writers	11
Dissemination policy: reproducible research	#31c	Plans, if any, for granting public access to the full protocol, participant-level dataset, and statistical code	11
**Appendices**			
Informed consent materials	#32	Model consent form and other related documentation given to participants and authorised surrogates	n/a
Biological specimens	#33	Plans for collection, laboratory evaluation, and storage of biological specimens for genetic or molecular analysis in the current trial and for future use in ancillary studies, if applicable	n/a

The SPIRIT checklist is distributed under the terms of the Creative Commons Attribution License CC-BY-ND 3.0. This checklist was completed on 03. April 2020 using https://www.goodreports.org/, a tool made by the EQUATOR Network in collaboration with Penelope.ai

For peer review only

De-implementation strategy to reduce overtreatment of asymptomatic bacteriuria: a study protocol for a stepped-wedge cluster randomized trial

Journal:	*BMJ Open*
Manuscript ID	bmjopen-2020-039085.R1
Article Type:	Protocol
Date Submitted by the Author:	04-Aug-2020
Complete List of Authors:	van Horrik, Tessa; Amsterdam UMC - Locatie AMC, Internal Medicine, Infectious Diseases<br>Geerlings, Suzanne; Amsterdam UMC - Locatie AMC, Internal Medicine, Infectious Diseases<br>Stalenhoef, Janneke; OLVG Locatie Oost, Internal Medicine, Infectious Diseases<br>van Nieuwkoop, Cees; HagaZiekenhuis, Internal Medicine, Infectious Diseases<br>Saanen, Joppe; Amsterdam UMC - Locatie AMC, Emergency Medicine<br>Schneeberger, Caroline; Amsterdam UMC - Locatie AMC, Medical Microbiology<br>Laan, Bart; Amsterdam UMC - Locatie AMC, Internal Medicine, Infectious Diseases
**Primary Subject Heading**:	Infectious diseases
Secondary Subject Heading:	Emergency medicine, Infectious diseases
Keywords:	Urinary tract infections < UROLOGY, INFECTIOUS DISEASES, Diagnostic microbiology < INFECTIOUS DISEASES

SCHOLARONE™
Manuscripts

I, the Submitting Author has the right to grant and does grant on behalf of all authors of the Work (as defined in the below author licence), an exclusive licence and/or a non-exclusive licence for contributions from authors who are: i) UK Crown employees; ii) where BMJ has agreed a CC-BY licence shall apply, and/or iii) in accordance with the terms applicable for US Federal Government officers or employees acting as part of their official duties; on a worldwide, perpetual, irrevocable, royalty-free basis to BMJ Publishing Group Ltd ("BMJ") its licensees and where the relevant Journal is co-owned by BMJ to the co-owners of the Journal, to publish the Work in this journal and any other BMJ products and to exploit all rights, as set out in our licence.

The Submitting Author accepts and understands that any supply made under these terms is made by BMJ to the Submitting Author unless you are acting as an employee on behalf of your employer or a postgraduate student of an affiliated institution which is paying any applicable article publishing charge ("APC") for Open Access articles. Where the Submitting Author wishes to make the Work available on an Open Access basis (and intends to pay the relevant APC), the terms of reuse of such Open Access shall be governed by a Creative Commons licence – details of these licences and which Creative Commons licence will apply to this Work are set out in our licence referred to above.

Other than as permitted in any relevant BMJ Author's Self Archiving Policies, I confirm this Work has not been accepted for publication elsewhere, is not being considered for publication elsewhere and does not duplicate material already published. I confirm all authors consent to publication of this Work and authorise the granting of this licence.

For peer review only

TITLE PAGE

Title: De-implementation strategy to reduce overtreatment of asymptomatic bacteriuria: a study protocol for a stepped-wedge cluster randomized trial

Authors: T. M. Z. X. K. van Horrik[1], S. E. Geerlings[1], J. E. Stalenhoef[2], C. van Nieuwkoop[3], J. B. Saanen[4], C. Schneeberger-van der Linden[5], B. J. Laan[1]

[1]Amsterdam UMC, University of Amsterdam, Internal Medicine, Infectious Diseases, Amsterdam, The Netherlands

[2]OLVG, Internal Medicine, Infectious Diseases, Amsterdam, The Netherlands

[3]Haga Teaching Hospital, Internal Medicine, Infectious Diseases, The Hague, The Netherlands

[4]Amsterdam UMC, University of Amsterdam, Emergency Medicine, Amsterdam, The Netherlands

[5]Amsterdam UMC, University of Amsterdam, Medical Microbiology, Amsterdam, The Netherlands

Correspondence to:

Tessa M. Z. X. K. van Horrik, Amsterdam UMC, University of Amsterdam, Internal Medicine, Infectious Diseases, Meibergdreef 9, Amsterdam, Netherlands, +31 20 5665992, t.m.vanhorrik@amsterdamumc.nl

Word count: 3194

For peer review only - http://bmjopen.bmj.com/site/about/guidelines.xhtml

ABSTRACT

Introduction Antimicrobial treatment of asymptomatic bacteriuria (ASB) is one of the most common unnecessary uses of antimicrobials. Earlier studies have shown that this inappropriate treatment has a prevalence of up to 45%. Multifaceted interventions based on international guidelines and antimicrobial stewardship can decrease overtreatment of ASB. We have designed a study protocol with the main objective of reducing overtreatment of ASB by 50% through use of a de-implementation strategy.

Methods and analysis We will use a stepped-wedge cluster randomized design for our multifaceted de-implementation strategy, comparing outcomes before and after introduction of our intervention in the emergency department of five hospitals (clusters) in the Netherlands. All patients (≥18 years old) who have a urine test performed in the emergency department will be screened for eligibility. The de-implementation strategy consists of a combination of interventions, including education, audit and feedback. The primary endpoint will be overtreatment of ASB in patients without risk factors (e.g. pregnancy, planned invasive urologic procedures and neutropenia). Secondary endpoints are the duration of antimicrobial treatment for ASB, the number of urine cultures and urinalysis per 1000 patients, and overtreatment of positive urinalysis in asymptomatic patients.

Ethics and dissemination

Ethical approval was obtained from Medical Ethics Research Committee of the Academic Medical Centre, (Amsterdam, the Netherlands) with a waiver for informed consent. Local feasibility will be obtained by the local institutional review boards of all participating hospitals. Our study aims to reduce inappropriate screening and treatment of ASB in emergency departments, improve healthcare quality, lower the increase in antimicrobial resistance, and save costs. If proven (cost)-effective, this study provides a well suited strategy for a nationwide approach to reduce overtreatment of ASB.

Registration details This trial is registered at Netherlands Trial Register, trial NL8242, trialregister.nl/trial/8242.

Keywords asymptomatic bacteriuria, urinary tract infections, antimicrobial stewardship, implementation science, unnecessary procedures.

For peer review only - http://bmjopen.bmj.com/site/about/guidelines.xhtml

STRENGTHS AND LIMITATIONS OF THIS STUDY

- We will use a stepped-wedge cluster randomized design, ultimately resulting in implementation of our intervention in all participating hospitals.

- Our strategy is well suited for broad-scale de-implementation in emergency departments.

- A possible limitation of this study is that we are not able to evaluate the impact of an individual intervention, since we are using a multifaceted de-implementation strategy.

- Another possible limitation is that we are limited by documentation in the patient files and therefore, we might overestimate the number of patients with asymptomatic bacteriuria.

For peer review only

For peer review only - http://bmjopen.bmj.com/site/about/guidelines.xhtml

INTRODUCTION

There is a growing urgency to improve healthcare quality and save costs by implementing practices to reduce unnecessary diagnostics, diagnostic error and related overtreatment, so called low-value care practices. This is exemplified by international campaigns, such as Choosing Wisely and Do not Do prompts, that were launched to reduce unnecessary care in several medical services, resulting in less risks for the patients, saving costs and increasing value for care.[1 2] A parallel campaign initiative, titled 'To Do or Not to Do?' was introduced to de-implement unnecessary care in the Netherlands.[3]

Antimicrobial resistance, as a result of antibiotic overconsumption, is a global threat to public health and there is global consensus about reducing the use of antibiotics.[4] Urinary tract infections (UTI) are one of the most common categories of infections for which antimicrobials are prescribed.[5] However, not all antimicrobial prescriptions are necessary. This is especially true for asymptomatic bacteriuria (ASB), which is the presence of bacteria in the urine of a patient who does not have symptoms of a UTI. ASB is a common finding, especially among women, elderly, and patients with urinary catheters. The prevalence of ASB in adults over 65 years is high, since the prevalence of ASB increases with age.[6] Guidelines strongly recommend not to screen for or treat ASB with antimicrobials, except for specific patients at risk of developing a complicated UTI.[5] This is also outlined in the American Choosing Wisely campaign, which judges treatment of ASB as one of five low-value care services that should be avoided.[7] In 2015, this resulted in the recommendation: "Do not use antimicrobials to treat bacteriuria in older adults unless specific urinary tract symptoms are present".[7]

Regardless, antimicrobials are still frequently used for patients with ASB. A systematic review and meta-analysis from 2017 showed a pooled prevalence of 45% among 4129 ASB cases (95% CI 39-50) who received inappropriate antimicrobial treatment.[8] The prevalence was even higher in a recent retrospective evaluation of 25 hospitals in the United States of America (USA), in which 64% of the 2225 positive urine cultures were classified as ASB and 72% of 961 patients with ASB were treated with antimicrobials.[9] The exact use of antimicrobials for ASB in hospitals in the Netherlands is unknown. However, in 2015, a prospective study performed in ten nursing homes in the Netherlands showed that 115 (32%) of 356 residents with a possible UTI were treated inappropriately, and treatment of ASB was the most common reason.[10] These results indicate that reducing the treatment of ASB is an appropriate target of antimicrobial stewardship strategies.

For peer review only - http://bmjopen.bmj.com/site/about/guidelines.xhtml

Earlier studies demonstrated that multifaceted interventions can effectively reduce overtreatment of ASB in nursing home residents and patients with urinary catheters. In 2005, a cluster randomized trial in 12 nursing homes in Canada and the USA showed a reduction in antimicrobial use after the multifaceted implementation of a diagnostic and treatment algorithm (1.17 versus 1.59 courses of antimicrobials/1000 resident days).[11] For catheterized patients in the USA, a controlled before-after study in 2015 showed a decrease of urine culture ordering from 41.2 to 23.3 per 1000 bed-days.[12] Furthermore, short educational sessions combined with feedback in two academic tertiary acute care hospitals in Canada, performed in 2015, resulted in a reduction of overtreatment for ASB of 8% (2 of 24 patients) in the intervention group compared to 52% (14 of 29 patients) in the control group (OR 0.1; 95% CI 0.02-0.49).[13] In 2018, these findings resulted in the development of an implementation guide to reduce overtreatment of ASB.[14]

However, effectiveness studies after the launch of this implementation guide have not yet been performed in emergency departments (ED). The results of earlier studies suggest that improving ASB management in the emergency department is necessary. In 2017, a prospective observational study performed in the ED of a tertiary care centre in the USA showed that 27 of 71 (38%) older adults were not correctly diagnosed with a UTI.[15] Furthermore, overtreatment of a suspected (but unconfirmed) UTI was very common in a retrospective study performed in the emergency department of another tertiary care centre in the USA in 2013, where 63 of 66 (95%) patients were treated while they had a negative urine culture.[16]

In this study, our aim is to reduce overtreatment of ASB by 50% by using a multifaceted de-implementation strategy based on the implementation guide described above.

METHODS AND ANALYSIS

Study design and setting

For the development of our study protocol, we used the reporting guidelines for stepped-wedge cluster randomised trials.[17] We will use a repeated measurement stepped-wedge cluster randomized design. The de-implementation strategy will be introduced in the emergency department of five hospitals (clusters) in the Netherlands (one university and four general teaching hospitals) in six time periods consisting of one or two months, depending on how many patients will be included in the first month. Therefore, the total study duration is six to twelve months (Figure 1). The order in which the participating hospitals will receive the intervention

For peer review only - http://bmjopen.bmj.com/site/about/guidelines.xhtml

will be randomly determined by an independent data manager. Every cluster will start in control condition and by the end of the trial every cluster will have received the intervention. We planned to start our study on 1 May 2020. However, due to the COVID-19 pandemic, the start of our study will be postponed until 1 October 2020.

Patient selection

We will screen all patients (≥ 18 years old) who have urine tests (culture and/or urinalysis) that were obtained during an ED presentation (Figure 2). First, we will exclude all negative urinalyses (defined as absence of leukocyte esterase and nitrite in dipsticks, and absence of leukocytes and bacteria in microscopic analysis) and negative urine cultures (Table 1). Further exclusion criteria are patients with a symptomatic UTI (systemic or local symptoms, according to the Centre for Disease Control's (CDC) National Healthcare Safety Network for Urinary Tract Infection) (Table 1),[18] active treatment for a UTI on presentation, patients with an alternate site of infection for which they receive antimicrobial treatment, and patients with ASB and risk factors, defined as pregnant women, patients prior to planned invasive urologic procedures associated with mucosal trauma (including transurethral surgery of the prostate or bladder, ureteroscopy including lithotripsy, and percutaneous stone surgery), and high-risk neutropenia (defined as absolute neutrophil count <500 cells/µl).

Table 1 Definitions of positive urinalysis, urinary tract infection and asymptomatic bacteriuria

Item	Definition
Positive urinalysis[19]	Urinalysis must have one or more of the following results:<br>Dipstick<br>• Abnormal nitrites<br>• Abnormal leukocyte esterase<br>Microscopic analysis:<br>• >5 leukocytes/high-powered field (or reference ranges according to local standard operating procedures)<br>• Abnormal amount of bacteria according local operating procedures.
Asymptomatic bacteriuria[5]	The presence of one or more species of bacteria growing in the urine at specified quantitative counts ($\geq 10^5$ CFU/ml) regardless of the presence of pyuria, without UTI symptoms or signs.

For peer review only - http://bmjopen.bmj.com/site/about/guidelines.xhtml

Urinary tract infection[18]	Patient has at least one of the following signs or symptoms: fever (>38.0°C)†, suprapubic tenderness†, costovertebral angle pain or tenderness†, urinary urgency, urinary frequency, or dysuria* AND patient has a urine culture with no more than two species of organisms identified, at least one of which is a bacterium of ≥10^5 CFU/ml.
Catheter-associated* urinary tract infection[18]	A urinary tract infection where a urinary catheter was in place for >2 days on the date of event, with day of placement being day 1, and was in place on the date of event or the day before.

†With no other recognized cause. *A urinary catheter in situ could cause patient complaints of frequency, urgency, or dysuria. Therefore, these symptoms cannot be used in patients with catheters.

Primary and secondary endpoints

The primary endpoint is the percentage of patients with ASB without risk factors or another alternative site of infections, who are treated with antimicrobials. We define ASB according to the international guideline provided by the Infectious Diseases Society of America (IDSA) (Table 1).[5] Secondary endpoints are the duration of antimicrobial treatment for ASB, the number of urine cultures and urinalysis (dipsticks and microscopic analysis) per 1000 patients at the emergency department, the percentage of asymptomatic patients treated for urine cultures that are considered positive in daily practice; a quantitative count of bacteria ≥10^3 CFU/ml, and the percentage of patients without symptoms or risk factors treated with antimicrobials for a positive urinalysis without an obtained culture. Explorative analyses will evaluate the total number of urine cultures ordered in the hospital and indications for negative urine cultures during the baseline period.

Data collection

The study will collate a list of all urine cultures and urinalyses performed in the emergency department including corresponding microbiology laboratory and clinical chemistry data. All relevant patient information, including the presence of other possible infections, of those with positive cultures will be reviewed from medical and nursing records by a research physician to determine if patients have ASB. For patients with ASB, we will collect data on antimicrobial treatment, including antimicrobial prescription and duration of antimicrobial therapy, and the specified bacterial count and pathogen according to the standard operating procedure of the microbiology laboratory. For feasibility reasons, a random sample of a total number of 220 negative urine

For peer review only - http://bmjopen.bmj.com/site/about/guidelines.xhtml

cultures will be screened by medical record review to evaluate the indication for testing during the baseline period. We will use this data in the further development of an additional local de-implementation strategy based on the assessment of determinants of current practice. Furthermore, we will collect the patient level variables, including patient characteristics (age, gender, residency in nursing home), Charlson comorbidity index,[20] Modified Early Warning Score (MEWS),[21] chronic urinary catheter use, reason for presentation at the emergency department, and use of antimicrobials for uropathogens in the last seven days before presentation at the emergency department. We will also collect the reasons for urine testing documented in the medical record. Data from medical records will be collected in an electronic GCP-compliant database, and we will audit a random sample of 10% to assure the validity of data collection.

De-implementation strategy

Our de-implementation strategy is a tailored multifaceted intervention based on a Dutch de-implementation guide and an international implementation guide to reduce overtreatment of ASB in low-risk patients.[14 22] We will use a combination of interventions, such as education, audit and feedback and organizational adjustments, because these strategies have shown to be successful for reducing treatment of ASB in prior studies.[11 23] The de-implementation strategy can be divided into a general part that will be introduced in every hospital by our project group, and additional local strategies introduced by local healthcare workers, focused on the local setting (Table 2).

For the general strategy, we will start the intervention period by holding one or more educational meetings about overtreatment of ASB for all healthcare workers in the emergency department. During these educational meetings, we will inform all healthcare workers about the current ASB guidelines and discuss the local barriers and facilitators to reduce its overtreatment. Next to this, the results of the baseline period will be used as competitive feedback report between the participating hospitals. This competitive feedback report will be used to raise awareness among healthcare workers.[24] During this meeting we will distribute posters and pocket cards with an algorithm (Figure 3a and 3b) for appropriate indications for urine cultures and urinalysis, and overtreatment of ASB, based on current guidelines.[5 25] The kick-off meeting will be led by the project coordinator and the local champion. These tools will be available throughout the intervention period and after the intervention period, if necessary. We will send emails with the anonymous feedback report and the algorithm to healthcare workers who were not able to join the meeting.

For peer review only - http://bmjopen.bmj.com/site/about/guidelines.xhtml

For the additional local part of the strategy, a local champion, one of the leading physicians, will be appointed in each participating hospital and will be responsible for the de-implementation strategy. The local champion will start an asymptomatic bacteriuria quality improvement team (ASB-team), which will consist of at least an emergency doctor, infectious disease physician, a resident internal medicine, and a nurse from the emergency department. The ASB-team will be motivated to assess barriers and facilitators within their hospital and to start additional interventions based on the local setting. For example, by educating and/or reminding healthcare workers about appropriate urine testing and treatment for ASB, and evaluating and/or adjusting local protocols to reduce inappropriate urine testing and antimicrobial use. Further, the ASB-team will collaborate with the antibiotic stewardship teams. During the intervention period, the project coordinator will be in contact with the local champion and/or the ASB-team once every two weeks to exchange ideas and experiences based on interim results (ordering patterns of urine culture) and assessment of local facilitators and barriers.

Table 2 Summary of the de-implementation strategy

**Item**	**Description**
**General part**	
Educational meeting with competitive feedback	Combinations of strategies to reduce treatment of ASB.
Clinical decision tools	Pocket cards and posters with algorithms as an aid and reminder for appropriate and inappropriate urine testing and treatment for ASB.
**Local part**	
Local champion	One of the leading physicians who is responsible for the de-implementation strategy.
ASB-team	Consists of a local champion, emergency doctor, infectious disease physician, resident internal medicine, and nurse for the emergency department.
Additional interventions	Based on local setting. For example, education and/or reminding healthcare workers about appropriate urine testing and treatment of ASB.

Sample size

For peer review only - http://bmjopen.bmj.com/site/about/guidelines.xhtml

We will use a stepped-wedge hospital-randomized design with six time periods and five steps, in which one hospital will switch from control to treatment at each step. Previous studies have shown reduction of inappropriate treatment for ASB of approximately 50% following de-implementation strategies.[8] Therefore, we anticipate that the proportion of patients who received inappropriate antimicrobial treatment for ASB will be reduced by 50% from 0.450 to 0.225 after the de-implementation strategy. Assuming an intra-hospital correlation coefficient of 0.10, a total of at least 420 patients (= 14 patients * 6 time periods * 5 hospitals) will achieve 80% power to detect this reduction using the two-sided Wald Z-Test with a significance level of 0.05. We used PASS version 15.0.10 to calculate this sample size. During the same time period, we will include additional patients with positive urinalyses.

Statistical analysis

Effect evaluation

Categorical data were summarised as frequency and percentage, and continuous data as mean (standard deviation) or median (range). We will use fixed effects models to analyse the overall effect of our de-implementation strategy on overtreatment of ASB.[26] To adjust for clustering and temporal trends, we will include the clusters (hospitals) as a categorical predictor and the time in months as a fixed effect in the model. To correct for the Level 2 variance, we will multiply the standard error estimates by the DEFT, which is the square root of the design effect. We will evaluate the differences between baseline period and intervention period according an intention to treat principle. To adjust for confounders, we will include the main risk factors for overtreatment of ASB (namely age, acutely altered mental status, and positive urinalysis)[27] in the model. We will use the Akaike's Information Criterion and ANOVA tables to compare the fitting of the models. We will report the results of the adjusted, unadjusted, and intercept model. A two-sided p value < 0.05 was considered significant.

Process evaluation

During the planned regular meetings with members of the local ASB teams to report results and feedback, we will evaluate the introduced interventions. By exchanging thoughts and experiences on the implemented interventions, we will be able to determine what intervention(s) will work best in daily practice. For the process evaluation, we will evaluate the de-implementation strategy adherence and compliance. Therefore, all elements of the de-implementation strategy will be explored in all participating hospitals. We will award the hospitals with

For peer review only - http://bmjopen.bmj.com/site/about/guidelines.xhtml

feedback points for all elements of the de-implementation strategy.[28] For example, points will be given for the number of persons present at the planned meetings and for every strategy that is implemented.

Economic evaluation

We will primarily perform a cost-effectiveness analysis, in which we evaluate the cost effectiveness ratio (CER) (CER = cost of de-implementation / healthcare cost reduction). Our goal is to achieve a reduction in urine culture, urinalysis, and antimicrobial prescriptions, and thereby a reduction in costs. The costs of our de-implementation strategy will be divided in non-recurrent study-related costs and recurrent de-implementation-related costs. Non-recurrent study-related costs include material costs, developmental costs of the de-implementation strategy, and costs of evaluation of the de-implementation. Recurrent costs include the costs to introduce the de-implementation strategy, such as time of the extra meetings of the ASB-team and time invested by the local champion. For feasibility reasons, we will use the number of all urine cultures, urinalyses and antimicrobial prescriptions, representing the main cost benefits due to our strategy. The costs for urine cultures, urinalyses, and antimicrobials used in the analyses will be based on Dutch guideline on healthcare economic evaluations.[29] The result of the cost-effectiveness analysis will be used in a budget impact analysis to assess the financial impact on the hospital's budget to implement and sustain the de-implementation strategy.

ETHICS AND DISSEMINATION

Ethical approval was obtained on 13 December 2019 from Medical Ethics Research Committee of the Academic Medical Centre (Amsterdam, the Netherlands), with a waiver for informed consent. Local feasibility will be obtained by the local institutional scientific review boards of all participated hospitals. This trial is registered at Netherlands Trial Register, trial NL8242, trialregister.nl/trial/8242. All relevant results of our study will be disseminated through publications in peer-reviewed journals and presentations at relevant (scientific) conferences. No identifiable patient data will be disseminated.

Patient and Public Involvement

This research will be performed without patient involvement. Patients will not be invited to comment on the study design and will not be consulted to develop patient relevant outcomes or interpret the results. Patients will not be invited to contribute to the writing or editing of this document for readability or accuracy.

For peer review only - http://bmjopen.bmj.com/site/about/guidelines.xhtml

For peer review only

DISCUSSION

This study protocol describes the design, de-implementation strategy and process evaluation to reduce overtreatment of ASB in the daily practice of emergency medicine. Our de-implementation strategy could prevent the use of unnecessary urine diagnostics and subsequent inappropriate treatment of ASB, improve healthcare quality, lower the increase in antimicrobial resistance, and save costs. If proven to be (cost) effective, this study could provide a nationwide strategy to reduce overtreatment of ASB.

In our study, we will not evaluate the clinical outcome of our strategy, including potential clinical benefits, such as reduced side effects of antibiotics, since previous studies have already shown that ASB in non-risk patients does not lead to an increased risk of symptomatic UTI and that it is safe to not treat ASB.[30 31]

By using a stepped-wedge cluster randomized design, we will be able to compare the outcomes within a cluster between the time intervals in which a cluster received the control and the interventions. Furthermore, our intervention will be enrolled in a systematic manner and eventually be introduced in all participating hospitals. A novel part of our study compared to previous studies that aimed to reduce overtreatment of ASB is that we will be using local champions in each hospital and adjust the intervention based on the local situation.

A limitation of stepped wedge designs is that this require larger sample sizes, as patients' characteristics may be similar within one cluster. By randomization of the start time of the clusters, we intend to balance these important characteristics. However, this cannot be ensured with our relatively small number of clusters.[32] Another possible limitation is that we will not prospectively assess clinical symptoms ourselves, and are limited to documentation of symptomatology in patient files. This might overestimate the number of patients with asymptomatic bacteriuria in the baseline and intervention periods. Since this should be similar in the baseline and intervention periods, we do not expect that this will influence our results. Furthermore, we cannot evaluate the impact of an individual intervention. However, we expect that our de-implementation strategy is low cost and well suitable for broad scale implementation.

For peer review only - http://bmjopen.bmj.com/site/about/guidelines.xhtml

REFERENCES

1. The National Institute for Health and Care Excellence (NICE). NICE Do not Do prompts 2013 [Available from: https://www.nice.org.uk/ sharedlearning/nice-do-not-do-prompts accessed 09-09-2019.

2. American Board of Internal Medicine Foundation. Choosing Wisely [Available from: http://www.choosingwisely.org/ accessed 09-09-2019.

3. Nederlandse Federatie van UMC. Doen of laten? [Available from: https://www.doenoflaten.nl/ accessed 09-09-2019.

4. World Health Organization. Global action plan on antimicrobial resistance. Geneva2015 [Available from: https://apps.who.int/iris/bitstream/handle/10665/193736/9789241509763_eng.pdf?sequence=1 accessed 10-10-2019.

5. Nicolle LE, Gupta K, Bradley SF, et al. Clinical Practice Guideline for the Management of Asymptomatic Bacteriuria: 2019 Update by the Infectious Diseases Society of Americaa. *Clinical infectious diseases : an official publication of the Infectious Diseases Society of America* 2019 doi: 10.1093/cid/ciy1121 [published Online First: 2019/03/22]

6. Nicolle LE. Asymptomatic bacteriuria: when to screen and when to treat. *Infectious disease clinics of North America* 2003;17(2):367-94. [published Online First: 2003/07/10]

7. Choosing Wisely: an initiative of the ABIM Foundation. Ten things physicians and patients should question 2013 [Available from: https://www.choosingwisely.org/doctor-patient-lists/american-geriatrics-society/ accessed 09-09-2019.

8. Flokas ME, Andreatos N, Alevizakos M, et al. Inappropriate Management of Asymptomatic Patients With Positive Urine Cultures: A Systematic Review and Meta-analysis. *Open forum infectious diseases* 2017;4(4):ofx207. doi: 10.1093/ofid/ofx207 [published Online First: 2017/12/12]

9. Spivak ES, Burk M, Zhang R, et al. Management of Bacteriuria in Veterans Affairs Hospitals. *Clinical infectious diseases : an official publication of the Infectious Diseases Society of America* 2017;65(6):910-17. doi: 10.1093/cid/cix474 [published Online First: 2017/05/23]

10. van Buul LW, Veenhuizen RB, Achterberg WP, et al. Antibiotic prescribing in Dutch nursing homes: how appropriate is it? *J Am Med Dir Assoc* 2015;16(3):229-37. doi: 10.1016/j.jamda.2014.10.003 [published Online First: 2014/12/03]

11. Loeb M, Brazil K, Lohfeld L, et al. Effect of a multifaceted intervention on number of antimicrobial prescriptions for suspected urinary tract infections in residents of nursing homes: cluster randomised

For peer review only - http://bmjopen.bmj.com/site/about/guidelines.xhtml

controlled trial. *BMJ (Clinical research ed)* 2005;331(7518):669. doi: 10.1136/bmj.38602.586343.55 [published Online First: 2005/09/10]

12. Trautner BW, Grigoryan L, Petersen NJ, et al. Effectiveness of an Antimicrobial Stewardship Approach for Urinary Catheter-Associated Asymptomatic Bacteriuria. *JAMA internal medicine* 2015;175(7):1120-7. doi: 10.1001/jamainternmed.2015.1878 [published Online First: 2015/05/27]
13. Irfan N, Brooks A, Mithoowani S, et al. A Controlled Quasi-Experimental Study of an Educational Intervention to Reduce the Unnecessary Use of Antimicrobials For Asymptomatic Bacteriuria. *PloS one* 2015;10(7):e0132071. doi: 10.1371/journal.pone.0132071 [published Online First: 2015/07/17]
14. Daniel M, Keller S, Mozafarihashjin M, et al. An Implementation Guide to Reducing Overtreatment of Asymptomatic Bacteriuria. *JAMA internal medicine* 2018;178(2):271-76. doi: 10.1001/jamainternmed.2017.7290 [published Online First: 2017/12/12]
15. Caterino JM, Leininger R, Kline DM, et al. Accuracy of Current Diagnostic Criteria for Acute Bacterial Infection in Older Adults in the Emergency Department. *Journal of the American Geriatrics Society* 2017;65(8):1802-09. doi: 10.1111/jgs.14912 [published Online First: 2017/04/26]
16. Gordon LB, Waxman MJ, Ragsdale L, et al. Overtreatment of presumed urinary tract infection in older women presenting to the emergency department. *Journal of the American Geriatrics Society* 2013;61(5):788-92. doi: 10.1111/jgs.12203 [published Online First: 2013/04/18]
17. Hemming K, Haines TP, Chilton PJ, et al. The stepped wedge cluster randomised trial: rationale, design, analysis, and reporting. *BMJ (Clinical research ed)* 2015;350:h391. doi: 10.1136/bmj.h391 [published Online First: 2015/02/11]
18. CDC National Healthcare Safety Network (NHSN). Patient Safety Component Manual 2019 [Available from: https://www.cdc.gov/nhsn/pdfs/pscmanual/pcsmanual_current.pdf accessed 26-09-2019.
19. Deville WL, Yzermans JC, van Duijn NP, et al. The urine dipstick test useful to rule out infections. A meta-analysis of the accuracy. *BMC urology* 2004;4:4. doi: 10.1186/1471-2490-4-4 [published Online First: 2004/06/04]
20. Quan H, Li B, Couris CM, et al. Updating and validating the Charlson comorbidity index and score for risk adjustment in hospital discharge abstracts using data from 6 countries. *American journal of epidemiology* 2011;173(6):676-82. doi: 10.1093/aje/kwq433 [published Online First: 2011/02/19]

For peer review only - http://bmjopen.bmj.com/site/about/guidelines.xhtml

21. Subbe CP, Kruger M, Rutherford P, et al. Validation of a modified Early Warning Score in medical admissions. *QJM* 2001;94(10):521-6. doi: 10.1093/qjmed/94.10.521 [published Online First: 2001/10/06]

22. van Dulmen S, Kool T, Verkerk E. Deïmplementatiegids 2019 [Available from: https://www.doenoflaten.nl/pdf/deïmplementatiegids_jan_2019.pdf accessed 10-10-2019.

23. McMaughan DK, Nwaiwu O, Zhao H, et al. Impact of a decision-making aid for suspected urinary tract infections on antibiotic overuse in nursing homes. *BMC geriatrics* 2016;16:81. doi: 10.1186/s12877-016-0255-9 [published Online First: 2016/04/17]

24. Spoorenberg V, Hulscher ME, Geskus RB, et al. A Cluster-Randomized Trial of Two Strategies to Improve Antibiotic Use for Patients with a Complicated Urinary Tract Infection. *PloS one* 2015;10(12):e0142672. doi: 10.1371/journal.pone.0142672 [published Online First: 2015/12/05]

25. Verenso. Urineweginfecties bij kwetsbare ouderen 2018 [Available from: https://www.verenso.nl/richtlijnen-en-praktijkvoering/richtlijnendatabase/urineweginfecties accessed 11-10-2019.

26. McNeish D, Stapleton LM. Modeling Clustered Data with Very Few Clusters. *Multivariate Behav Res* 2016;51(4):495-518. doi: 10.1080/00273171.2016.1167008 [published Online First: 2016/06/09]

27. Petty LA, Vaughn VM, Flanders SA, et al. Risk Factors and Outcomes Associated With Treatment of Asymptomatic Bacteriuria in Hospitalized Patients. *JAMA internal medicine* 2019 doi: 10.1001/jamainternmed.2019.2871 [published Online First: 2019/08/27]

28. Laan BJ, Maaskant JM, Spijkerman IJB, et al. De-implementation strategy to reduce inappropriate use of intravenous and urinary catheters (RICAT): a multicentre, prospective, interrupted time-series and before and after study. *The Lancet Infectious diseases* 2020 doi: 10.1016/S1473-3099(19)30709-1 [published Online First: 2020/03/11]

29. Hakkaart-van Roijen L, van der Linden N, Bouwmans C, et al. Kostenhandleiding: Methodologie van kostenonderzoek en referentieprijzen voor economische evaluaties in de gezondheidszorg 2016 [Available from: https://www.zorginstituutnederland.nl/publicaties/publicatie/2016/02/29/richtlijn-voor-het-uitvoeren-van-economische-evaluaties-in-de-gezondheidszorg accessed 27-09-2019.

30. Harding GK, Zhanel GG, Nicolle LE, et al. Antimicrobial treatment in diabetic women with asymptomatic bacteriuria. *The New England journal of medicine* 2002;347(20):1576-83. doi: 10.1056/NEJMoa021042 [published Online First: 2002/11/15]

For peer review only - http://bmjopen.bmj.com/site/about/guidelines.xhtml

31. Bengtsson C, Bengtsson U, Bjorkelund C, et al. Bacteriuria in a population sample of women: 24-year follow-up study. Results from the prospective population-based study of women in Gothenburg, Sweden. *Scandinavian journal of urology and nephrology* 1998;32(4):284-9. [published Online First: 1998/10/09]

32. Ellenberg SS. The Stepped-Wedge Clinical Trial: Evaluation by Rolling Deployment. *Jama* 2018;319(6):607-08. doi: 10.1001/jama.2017.21993 [published Online First: 2018/02/17]

For peer review only

For peer review only - http://bmjopen.bmj.com/site/about/guidelines.xhtml

AUTHORS' CONTRIBUTIONS

BJL and SEG were involved in the conception of the study and coordinated the funding application. TVH, SEG and BJL drafted the manuscript. BJL outlined the statistical analysis with support of the Clinical Research Unit of the Academic Medical Centre (Amsterdam, the Netherlands). JES, CN, JBS, and CS revised the manuscript critically for important intellectual content. All authors read and approved the final version of the manuscript.

FUNDING STATEMENT

This study is supported by the Netherlands Organisation for Health Research and Development (ZonMw) grant number 839205002. They will have no role in the study design or analysis.

COMPETING INTERESTS STATEMENT

The authors declare that they have no competing interests.

ACKNOWLEDGEMENTS

We thank Vanessa C. Harris (physician Infectious Diseases, Internal Medicine, Amsterdam UMC, University of Amsterdam, Netherlands), for proof-reading and spell checking the manuscript.

FIGURES

Figure 1 The stepped wedge design for de-implementation strategy.

Figure 2 Study profile. ASB=Asymptomatic bacteriuria. UTI=Urinary tract infection.

*Risk factors are pregnancy, patients prior to planned urologic procedures associated with mucosal trauma, and high-risk neutropenia.

Figure 3a Algorithm of indications for urinalysis and cultures in symptomatic patients.

ASB=Asymptomatic bacteriuria. UTI=Urinary tract infection. SWAB=Stichting Werkgroep Antibiotica Beleid (the Dutch Working Party on Antibiotic Policy).

Figure 3b Algorithm of indications for urinalysis and cultures in asymptomatic patients.

ASB=Asymptomatic bacteriuria. UTI=Urinary tract infection. SWAB=Stichting Werkgroep Antibiotica Beleid (the Dutch Working Party on Antibiotic Policy).

For peer review only - http://bmjopen.bmj.com/site/about/guidelines.xhtml

For peer review only

*Fever (>38°C), painful urination, urinary urgency, frequent miction, suprapubic pain, flank pain.

For peer review only

For peer review only - http://bmjopen.bmj.com/site/about/guidelines.xhtml

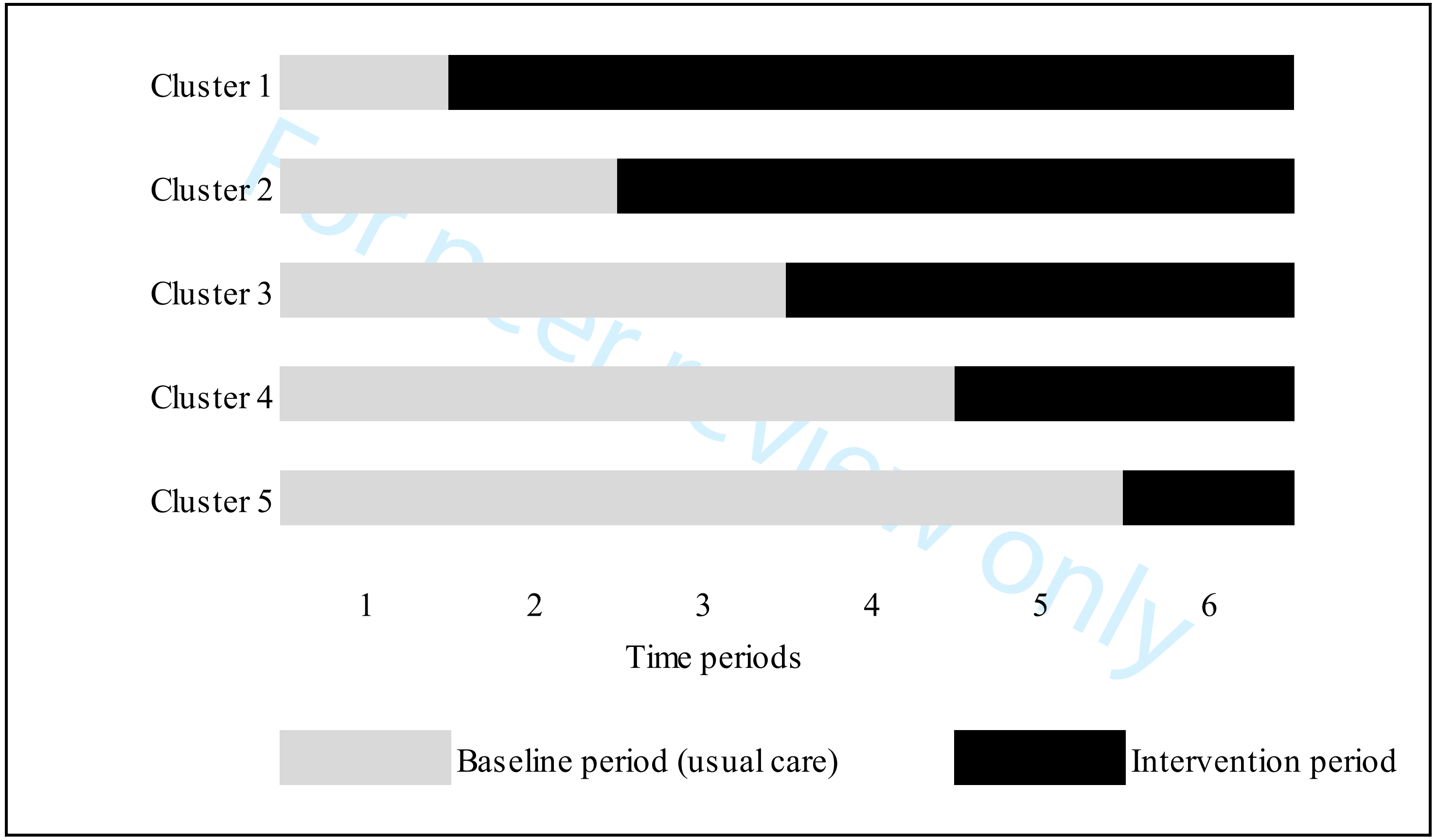

For peer review only

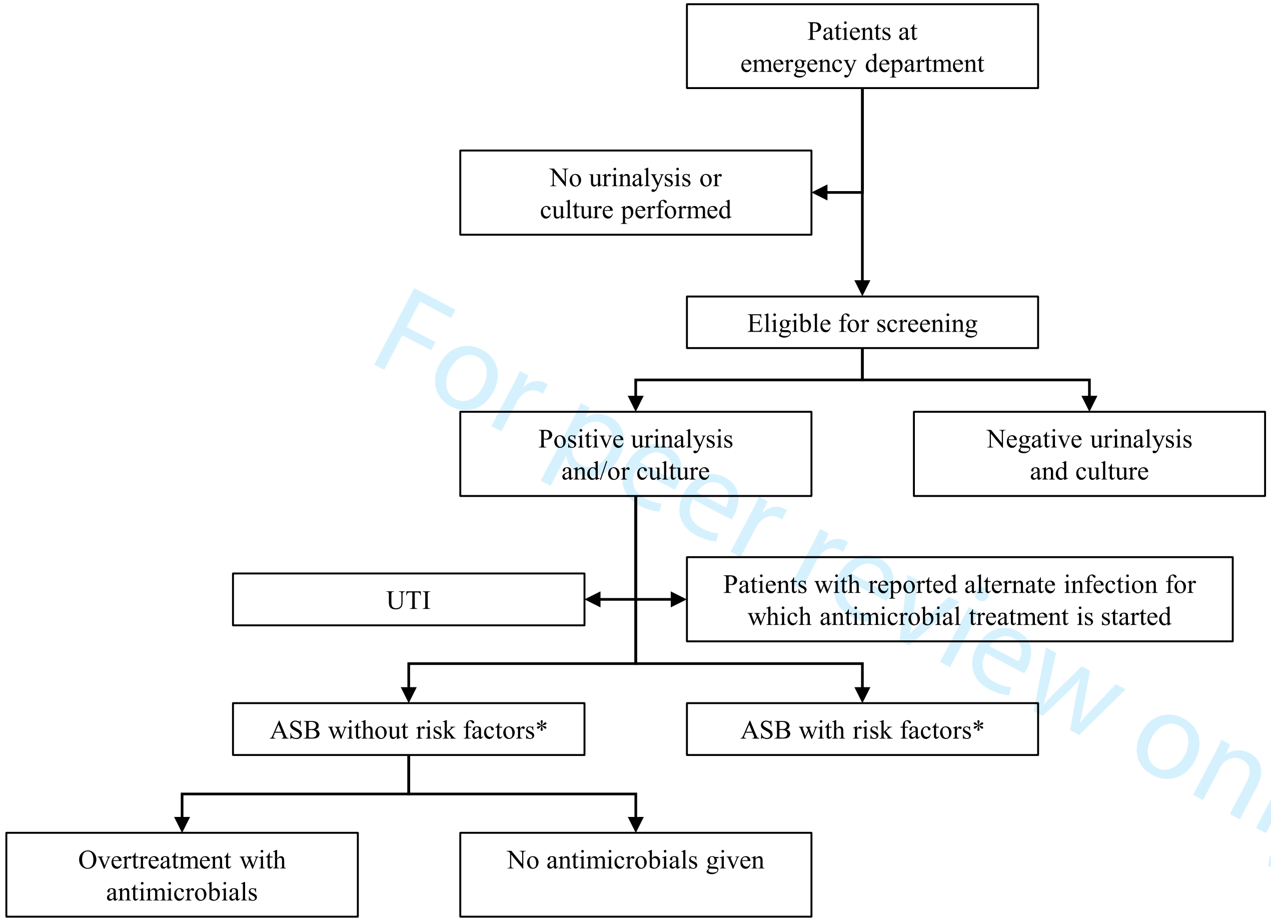

For peer review only

For peer review only - http://bmjopen.bmj.com/site/about/guidelines.xhtml

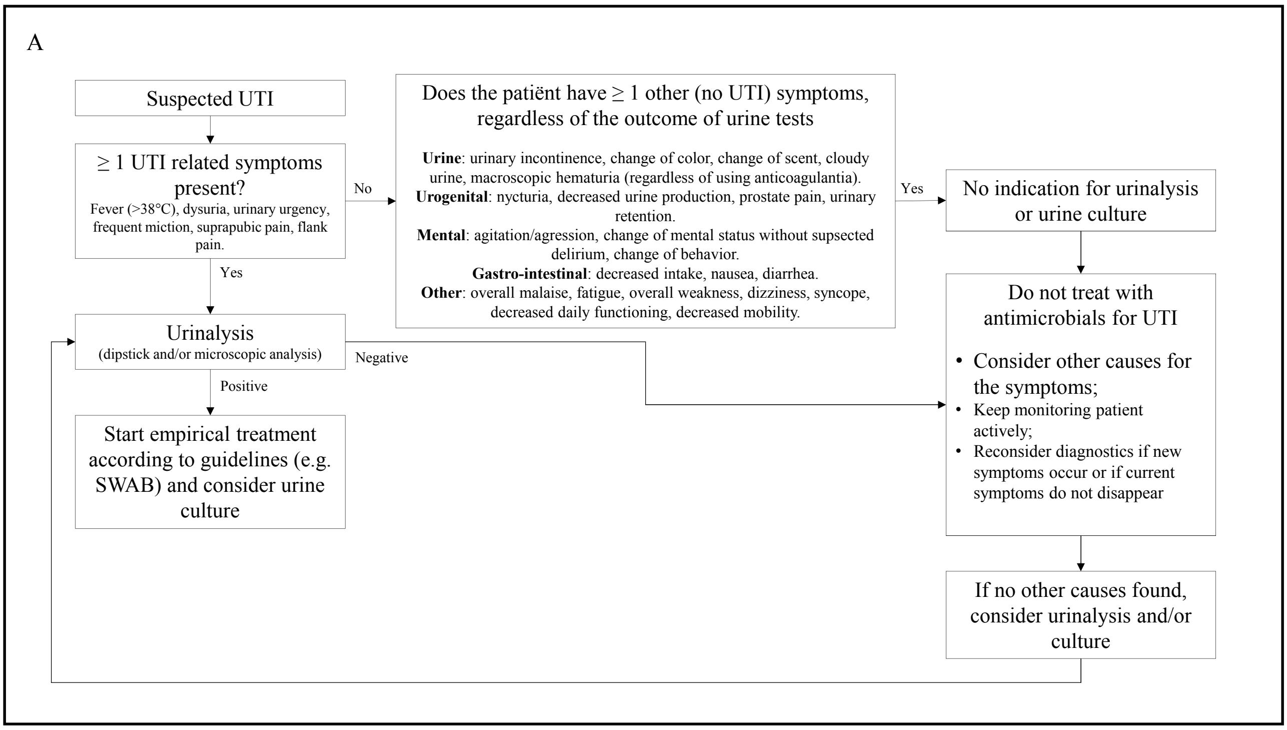
A
Suspected UTI
≥ 1 UTI related symptoms present?
Fever (>38°C), dysuria, urinary urgency, frequent miction, suprapubic pain, flank pain.
No
Does the patiënt have ≥ 1 other (no UTI) symptoms, regardless of the outcome of urine tests
Urine: urinary incontinence, change of color, change of scent, cloudy urine, macroscopic hematuria (regardless of using anticoagulantia).
Urogenital: nycturia, decreased urine production, prostate pain, urinary retention.
Mental: agitation/agression, change of mental status without supsected delirium, change of behavior.
Gastro-intestinal: decreased intake, nausea, diarrhea.
Other: overall malaise, fatigue, overall weakness, dizziness, syncope, decreased daily functioning, decreased mobility.
Yes
No indication for urinalysis or urine culture
Yes
Urinalysis
(dipstick and/or microscopic analysis)
Negative
Positive
Start empirical treatment according to guidelines (e.g. SWAB) and consider urine culture
Do not treat with antimicrobials for UTI
• Consider other causes for the symptoms;
• Keep monitoring patient actively;
• Reconsider diagnostics if new symptoms occur or if current symptoms do not disappear
If no other causes found, consider urinalysis and/or culture

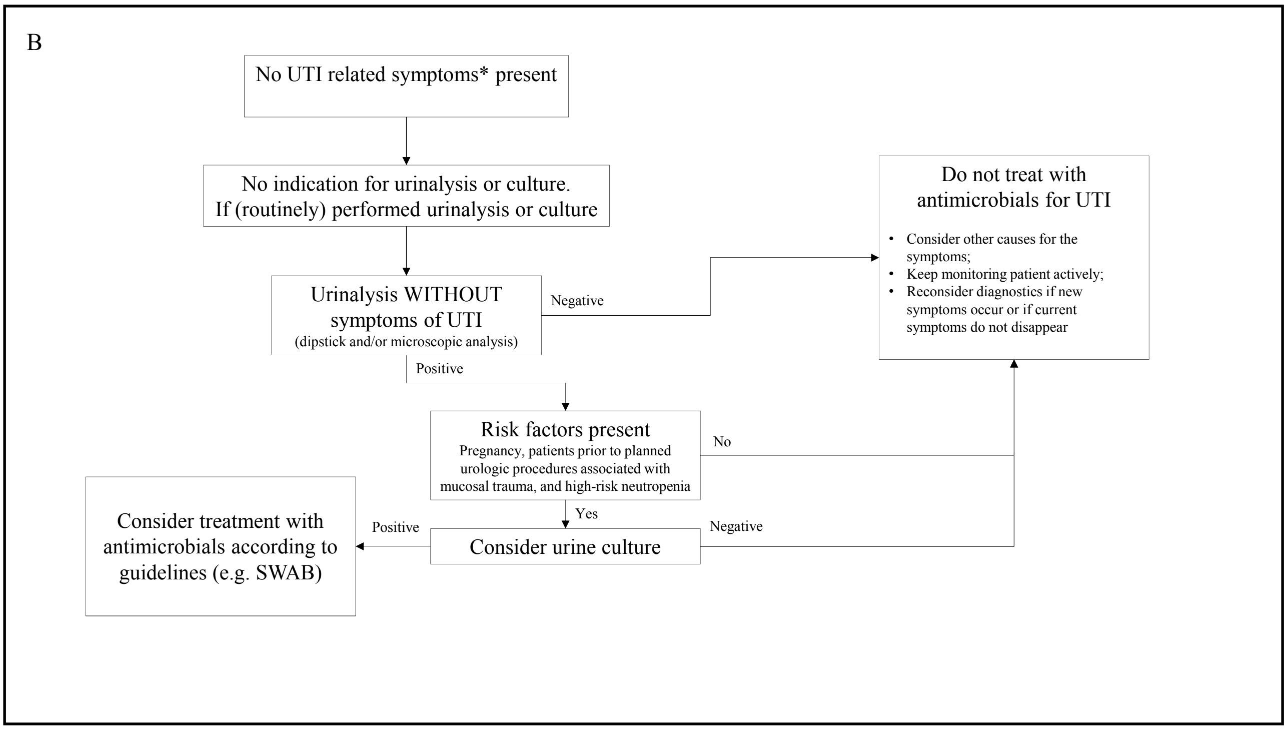
B
No UTI related symptoms* present
No indication for urinalysis or culture.
If (routinely) performed urinalysis or culture
Urinalysis WITHOUT symptoms of UTI
(dipstick and/or microscopic analysis)
Negative
Positive
Do not treat with antimicrobials for UTI
• Consider other causes for the symptoms;
• Keep monitoring patient actively;
• Reconsider diagnostics if new symptoms occur or if current symptoms do not disappear
Risk factors present
Pregnancy, patients prior to planned urologic procedures associated with mucosal trauma, and high-risk neutropenia
No
Yes
Consider urine culture
Negative
Positive
Consider treatment with antimicrobials according to guidelines (e.g. SWAB)

Reporting checklist for protocol of a clinical trial.

Based on the SPIRIT guidelines.

Instructions to authors

Complete this checklist by entering the page numbers from your manuscript where readers will find each of the items listed below.

Your article may not currently address all the items on the checklist. Please modify your text to include the missing information. If you are certain that an item does not apply, please write "n/a" and provide a short explanation.

Upload your completed checklist as an extra file when you submit to a journal.

In your methods section, say that you used the SPIRITreporting guidelines, and cite them as:

Chan A-W, Tetzlaff JM, Altman DG, Laupacis A, Gøtzsche PC, Krleža-Jerić K, Hróbjartsson A, Mann H, Dickersin K, Berlin J, Doré C, Parulekar W, Summerskill W, Groves T, Schulz K, Sox H, Rockhold FW, Rennie D, Moher D. SPIRIT 2013 Statement: Defining standard protocol items for clinical trials. Ann Intern Med. 2013;158(3):200-207

		Reporting Item	Page Number
**Administrative information**			
Title	#1	Descriptive title identifying the study design, population, interventions, and, if applicable, trial acronym	1
Trial registration	#2a	Trial identifier and registry name. If not yet registered, name of intended registry	11
Trial registration: data set	#2b	All items from the World Health Organization Trial Registration Data Set	11
Protocol version	#3	Date and version identifier	1
Funding	#4	Sources and types of financial, material, and other support	17
Roles and responsibilities: contributorship	#5a	Names, affiliations, and roles of protocol contributors	17

For peer review only

Roles and responsibilities: sponsor contact information	#5b	Name and contact information for the trial sponsor	17
Roles and responsibilities: sponsor and funder	#5c	Role of study sponsor and funders, if any, in study design; collection, management, analysis, and interpretation of data; writing of the report; and the decision to submit the report for publication, including whether they will have ultimate authority over any of these activities	17
Roles and responsibilities: committees	#5d	Composition, roles, and responsibilities of the coordinating centre, steering committee, endpoint adjudication committee, data management team, and other individuals or groups overseeing the trial, if applicable (see Item 21a for data monitoring committee)	17
**Introduction**			
Background and rationale	#6a	Description of research question and justification for undertaking the trial, including summary of relevant studies (published and unpublished) examining benefits and harms for each intervention	4
Background and rationale: choice of comparators	#6b	Explanation for choice of comparators	4
Objectives	#7	Specific objectives or hypotheses	4
Trial design	#8	Description of trial design including type of trial (eg, parallel group, crossover, factorial, single group), allocation ratio, and framework (eg, superiority, equivalence, non-inferiority, exploratory)	5
**Methods: Participants, interventions, and outcomes**			
Study setting	#9	Description of study settings (eg, community clinic, academic hospital) and list of countries where data will be collected. Reference to where list of study sites can be obtained	5
Eligibility criteria	#10	Inclusion and exclusion criteria for participants. If applicable, eligibility criteria for study centres and individuals who will	6

For peer review only

		perform the interventions (eg, surgeons, psychotherapists)	
Interventions: description	#11a	Interventions for each group with sufficient detail to allow replication, including how and when they will be administered	8
Interventions: modifications	#11b	Criteria for discontinuing or modifying allocated interventions for a given trial participant (eg, drug dose change in response to harms, participant request, or improving / worsening disease)	8
Interventions: adherance	#11c	Strategies to improve adherence to intervention protocols, and any procedures for monitoring adherence (eg, drug tablet return; laboratory tests)	9
Interventions: concomitant care	#11d	Relevant concomitant care and interventions that are permitted or prohibited during the trial	9
Outcomes	#12	Primary, secondary, and other outcomes, including the specific measurement variable (eg, systolic blood pressure), analysis metric (eg, change from baseline, final value, time to event), method of aggregation (eg, median, proportion), and time point for each outcome. Explanation of the clinical relevance of chosen efficacy and harm outcomes is strongly recommended	7
Participant timeline	#13	Time schedule of enrolment, interventions (including any run-ins and washouts), assessments, and visits for participants. A schematic diagram is highly recommended (see Figure)	5
Sample size	#14	Estimated number of participants needed to achieve study objectives and how it was determined, including clinical and statistical assumptions supporting any sample size calculations	9
Recruitment	#15	Strategies for achieving adequate participant enrolment to reach target sample size	6
**Methods: Assignment of interventions (for controlled trials)**			
Allocation: sequence generation	#16a	Method of generating the allocation sequence (eg, computer-generated random numbers), and list of any factors for stratification. To reduce predictability of a random sequence, details of any planned restriction (eg, blocking) should be provided in a separate document that is unavailable to those who enrol participants or assign interventions	5

For peer review only

Allocation concealment mechanism	#16b	Mechanism of implementing the allocation sequence (eg, central telephone; sequentially numbered, opaque, sealed envelopes), describing any steps to conceal the sequence until interventions are assigned	5
Allocation: implementation	#16c	Who will generate the allocation sequence, who will enrol participants, and who will assign participants to interventions	5
Blinding (masking)	#17a	Who will be blinded after assignment to interventions (eg, trial participants, care providers, outcome assessors, data analysts), and how	n/a
Blinding (masking): emergency unblinding	#17b	If blinded, circumstances under which unblinding is permissible, and procedure for revealing a participant's allocated intervention during the trial	n/a
**Methods: Data collection, management, and analysis**			
Data collection plan	#18a	Plans for assessment and collection of outcome, baseline, and other trial data, including any related processes to promote data quality (eg, duplicate measurements, training of assessors) and a description of study instruments (eg, questionnaires, laboratory tests) along with their reliability and validity, if known. Reference to where data collection forms can be found, if not in the protocol	7
Data collection plan: retention	#18b	Plans to promote participant retention and complete follow-up, including list of any outcome data to be collected for participants who discontinue or deviate from intervention protocols	7
Data management	#19	Plans for data entry, coding, security, and storage, including any related processes to promote data quality (eg, double data entry; range checks for data values). Reference to where details of data management procedures can be found, if not in the protocol	8
Statistics: outcomes	#20a	Statistical methods for analysing primary and secondary outcomes. Reference to where other details of the statistical analysis plan can be found, if not in the protocol	10
Statistics: additional analyses	#20b	Methods for any additional analyses (eg, subgroup and adjusted analyses)	10

For peer review only

Statistics: analysis population and missing data	#20c	Definition of analysis population relating to protocol non-adherence (eg, as randomised analysis), and any statistical methods to handle missing data (eg, multiple imputation)	10
**Methods: Monitoring**			
Data monitoring: formal committee	#21a	Composition of data monitoring committee (DMC); summary of its role and reporting structure; statement of whether it is independent from the sponsor and competing interests; and reference to where further details about its charter can be found, if not in the protocol. Alternatively, an explanation of why a DMC is not needed	n/a
Data monitoring: interim analysis	#21b	Description of any interim analyses and stopping guidelines, including who will have access to these interim results and make the final decision to terminate the trial	n/a
Harms	#22	Plans for collecting, assessing, reporting, and managing solicited and spontaneously reported adverse events and other unintended effects of trial interventions or trial conduct	11
Auditing	#23	Frequency and procedures for auditing trial conduct, if any, and whether the process will be independent from investigators and the sponsor	11
**Ethics and dissemination**			
Research ethics approval	#24	Plans for seeking research ethics committee / institutional review board (REC / IRB) approval	11
Protocol amendments	#25	Plans for communicating important protocol modifications (eg, changes to eligibility criteria, outcomes, analyses) to relevant parties (eg, investigators, REC / IRBs, trial participants, trial registries, journals, regulators)	n/a
Consent or assent	#26a	Who will obtain informed consent or assent from potential trial participants or authorised surrogates, and how (see Item 32)	11
Consent or assent: ancillary studies	#26b	Additional consent provisions for collection and use of participant data and biological specimens in ancillary studies, if applicable	11
Confidentiality	#27	How personal information about potential and enrolled participants will be collected, shared, and maintained in order to protect confidentiality before, during, and after the trial	11

For peer review only

Declaration of interests	#28	Financial and other competing interests for principal investigators for the overall trial and each study site	17
Data access	#29	Statement of who will have access to the final trial dataset, and disclosure of contractual agreements that limit such access for investigators	11
Ancillary and post trial care	#30	Provisions, if any, for ancillary and post-trial care, and for compensation to those who suffer harm from trial participation	n/a
Dissemination policy: trial results	#31a	Plans for investigators and sponsor to communicate trial results to participants, healthcare professionals, the public, and other relevant groups (eg, via publication, reporting in results databases, or other data sharing arrangements), including any publication restrictions	11
Dissemination policy: authorship	#31b	Authorship eligibility guidelines and any intended use of professional writers	11
Dissemination policy: reproducible research	#31c	Plans, if any, for granting public access to the full protocol, participant-level dataset, and statistical code	11
**Appendices**			
Informed consent materials	#32	Model consent form and other related documentation given to participants and authorised surrogates	n/a
Biological specimens	#33	Plans for collection, laboratory evaluation, and storage of biological specimens for genetic or molecular analysis in the current trial and for future use in ancillary studies, if applicable	n/a

The SPIRIT checklist is distributed under the terms of the Creative Commons Attribution License CC-BY-ND 3.0. This checklist was completed on 03. April 2020 using https://www.goodreports.org/, a tool made by the EQUATOR Network in collaboration with Penelope.ai

For peer review only

BMJ Open

De-implementation strategy to reduce overtreatment of asymptomatic bacteriuria: a study protocol for a stepped-wedge cluster randomized trial

Journal:	*BMJ Open*
Manuscript ID	bmjopen-2020-039085.R2
Article Type:	Protocol
Date Submitted by the Author:	22-Oct-2020
Complete List of Authors:	van Horrik, Tessa; Amsterdam UMC - Locatie AMC, Internal Medicine, Infectious Diseases<br>Geerlings, Suzanne; Amsterdam UMC - Locatie AMC, Internal Medicine, Infectious Diseases<br>Stalenhoef, Janneke; OLVG Locatie Oost, Internal Medicine, Infectious Diseases<br>van Nieuwkoop, Cees; HagaZiekenhuis, Internal Medicine, Infectious Diseases<br>Saanen, Joppe; Amsterdam UMC - Locatie AMC, Emergency Medicine<br>Schneeberger, Caroline; Amsterdam UMC - Locatie AMC, Medical Microbiology<br>Laan, Bart; Amsterdam UMC - Locatie AMC, Internal Medicine, Infectious Diseases
**Primary Subject Heading**:	Infectious diseases
Secondary Subject Heading:	Emergency medicine, Infectious diseases
Keywords:	Urinary tract infections < UROLOGY, INFECTIOUS DISEASES, Diagnostic microbiology < INFECTIOUS DISEASES

SCHOLARONE™
Manuscripts

I, the Submitting Author has the right to grant and does grant on behalf of all authors of the Work (as defined in the below author licence), an exclusive licence and/or a non-exclusive licence for contributions from authors who are: i) UK Crown employees; ii) where BMJ has agreed a CC-BY licence shall apply, and/or iii) in accordance with the terms applicable for US Federal Government officers or employees acting as part of their official duties; on a worldwide, perpetual, irrevocable, royalty-free basis to BMJ Publishing Group Ltd ("BMJ") its licensees and where the relevant Journal is co-owned by BMJ to the co-owners of the Journal, to publish the Work in this journal and any other BMJ products and to exploit all rights, as set out in our licence.

The Submitting Author accepts and understands that any supply made under these terms is made by BMJ to the Submitting Author unless you are acting as an employee on behalf of your employer or a postgraduate student of an affiliated institution which is paying any applicable article publishing charge ("APC") for Open Access articles. Where the Submitting Author wishes to make the Work available on an Open Access basis (and intends to pay the relevant APC), the terms of reuse of such Open Access shall be governed by a Creative Commons licence – details of these licences and which Creative Commons licence will apply to this Work are set out in our licence referred to above.

Other than as permitted in any relevant BMJ Author's Self Archiving Policies, I confirm this Work has not been accepted for publication elsewhere, is not being considered for publication elsewhere and does not duplicate material already published. I confirm all authors consent to publication of this Work and authorise the granting of this licence.

For peer review only

TITLE PAGE

Title: De-implementation strategy to reduce overtreatment of asymptomatic bacteriuria: a study protocol for a stepped-wedge cluster randomized trial

Authors: T. M. Z. X. K. van Horrik[1], S. E. Geerlings[1], J. E. Stalenhoef[2], C. van Nieuwkoop[3], J. B. Saanen[4], C. Schneeberger-van der Linden[5], B. J. Laan[1]

[1]Amsterdam UMC, University of Amsterdam, Internal Medicine, Infectious Diseases, Amsterdam, The Netherlands

[2]OLVG, Internal Medicine, Infectious Diseases, Amsterdam, The Netherlands

[3]Haga Teaching Hospital, Internal Medicine, Infectious Diseases, The Hague, The Netherlands

[4]Amsterdam UMC, University of Amsterdam, Emergency Medicine, Amsterdam, The Netherlands

[5]Amsterdam UMC, University of Amsterdam, Medical Microbiology, Amsterdam, The Netherlands

Correspondence to:

Tessa M. Z. X. K. van Horrik, Amsterdam UMC, University of Amsterdam, Internal Medicine, Infectious Diseases, Meibergdreef 9, Amsterdam, Netherlands, +31 20 5665992, t.m.vanhorrik@amsterdamumc.nl

Word count: 3092

For peer review only

ABSTRACT

Introduction Antimicrobial treatment of asymptomatic bacteriuria (ASB) is one of the most common unnecessary uses of antimicrobials. Earlier studies have shown that the prevalence this inappropriate treatment ranges from 45 to 83%. Multifaceted interventions based on international guidelines and antimicrobial stewardship can decrease overtreatment of ASB. We have designed a study protocol with the main objective of reducing overtreatment of ASB by 50% through use of a de-implementation strategy.

Methods and analysis We will use a stepped-wedge cluster randomized design for our multifaceted de-implementation strategy, comparing outcomes before and after introduction of our intervention in the emergency department of five hospitals (clusters) in the Netherlands. All patients (≥18 years old) who have a urine test performed in the emergency department will be screened for eligibility. The de-implementation strategy consists of a combination of interventions, including education, audit and feedback. The primary endpoint will be overtreatment of ASB in patients without risk factors (e.g. pregnancy, planned invasive urologic procedures and neutropenia). Secondary endpoints are the duration of antimicrobial treatment for ASB, the number of urine cultures and urinalysis per 1000 patients, and overtreatment of positive urinalysis in asymptomatic patients.

Ethics and dissemination

Ethical approval was obtained from Medical Ethics Research Committee of the Academic Medical Centre, (Amsterdam, the Netherlands) with a waiver for informed consent. Local feasibility will be obtained by the local institutional review boards of all participating hospitals. Our study aims to reduce inappropriate screening and treatment of ASB in emergency departments, improve healthcare quality, lower the increase in antimicrobial resistance, and save costs. If proven (cost)-effective, this study provides a well suited strategy for a nationwide approach to reduce overtreatment of ASB.

Registration details This trial is registered at Netherlands Trial Register, trial NL8242, trialregister.nl/trial/8242.

Keywords asymptomatic bacteriuria, urinary tract infections, antimicrobial stewardship, implementation science, unnecessary procedures.

For peer review only

STRENGTHS AND LIMITATIONS OF THIS STUDY

- We will use a stepped-wedge cluster randomized design, ultimately resulting in implementation of our intervention in all participating hospitals.
- Our strategy is well suited for broad-scale de-implementation in emergency departments.
- A possible limitation of this study is that we are not able to evaluate the impact of an individual intervention, since we are using a multifaceted de-implementation strategy.
- Another possible limitation is that we are limited by documentation in the patient files and therefore, we might overestimate the number of patients with asymptomatic bacteriuria.

For peer review only

INTRODUCTION

There is a growing urgency to improve healthcare quality and save costs by implementing practices to reduce unnecessary diagnostics, diagnostic error and related overtreatment, so called low-value care practices. This is exemplified by international campaigns, such as Choosing Wisely and Do not Do prompts, that were launched to reduce unnecessary care in several medical services, resulting in less risks for the patients, saving costs and increasing value for care.[1 2] A parallel campaign initiative, titled 'To Do or Not to Do?' was introduced to de-implement unnecessary care in the Netherlands.[3]

Antimicrobial resistance, as a result of antibiotic overconsumption, is a global threat to public health and there is global consensus about reducing the use of antibiotics.[4] Urinary tract infections (UTI) are one of the most common categories of infections for which antimicrobials are prescribed.[5] However, not all antimicrobial prescriptions are necessary. This is especially true for asymptomatic bacteriuria (ASB), which is the presence of bacteria in the urine of a patient who does not have symptoms of a UTI. ASB is a common finding, especially among women, elderly, and patients with urinary catheters. The prevalence of ASB in adults over 65 years is high, since the prevalence of ASB increases with age.[6] Guidelines strongly recommend not to screen for or treat ASB with antimicrobials, except for specific patients at risk of developing a complicated UTI.[5] This is also outlined in the American Choosing Wisely campaign, which judges treatment of ASB as one of five low-value care services that should be avoided.[7] In 2015, this resulted in the recommendation: "Do not use antimicrobials to treat bacteriuria in older adults unless specific urinary tract symptoms are present".[7]

Regardless, antimicrobials are still frequently used for patients with ASB. A systematic review and meta-analysis from 2017 showed a pooled prevalence of 45% among 4129 ASB cases (95% CI 39-50) who received inappropriate antimicrobial treatment.[8] The prevalence was even higher in a recent retrospective evaluation of 25 hospitals in the United States of America (USA), in which 64% of the 2225 positive urine cultures were classified as ASB and 72% of 961 patients with ASB were treated with antimicrobials.[9] The exact use of antimicrobials for ASB in hospitals in the Netherlands is unknown. However, in 2015, a prospective study performed in ten nursing homes in the Netherlands showed that 115 (32%) of 356 residents with a possible UTI were treated inappropriately, and treatment of ASB was the most common reason.[10] These results indicate that reducing the treatment of ASB is an appropriate target of antimicrobial stewardship strategies.

For peer review only - http://bmjopen.bmj.com/site/about/guidelines.xhtml

Earlier studies demonstrated that multifaceted interventions can effectively reduce overtreatment of ASB in nursing home residents and patients with urinary catheters. In 2005, a cluster randomized trial in 12 nursing homes in Canada and the USA showed a reduction in antimicrobial use after the multifaceted implementation of a diagnostic and treatment algorithm (1.17 versus 1.59 courses of antimicrobials/1000 resident days).[11] For catheterized patients in the USA, a controlled before-after study in 2015 showed a decrease of urine culture ordering from 41.2 to 23.3 per 1000 bed-days.[12] Furthermore, short educational sessions combined with feedback in two academic tertiary acute care hospitals in Canada, performed in 2015, resulted in a reduction of overtreatment for ASB of 8% (2 of 24 patients) in the intervention group compared to 52% (14 of 29 patients) in the control group (OR 0.1; 95% CI 0.02-0.49).[13] In 2018, these findings resulted in the development of an implementation guide to reduce overtreatment of ASB.[14]

However, effectiveness studies after the launch of this implementation guide have not yet been performed in emergency departments (ED). The results of earlier studies suggest that improving ASB management in the emergency department is necessary. In 2017, a prospective observational study performed in the ED of a tertiary care centre in the USA showed that 27 of 71 (38%) older adults were not correctly diagnosed with a UTI.[15] Furthermore, overtreatment of a suspected (but unconfirmed) UTI was very common in a retrospective study performed in the emergency department of another tertiary care centre in the USA in 2013, where 63 of 66 (95%) patients were treated while they had a negative urine culture.[16]

In this study, our aim is to reduce overtreatment of ASB by 50% by using a multifaceted de-implementation strategy based on the implementation guide described above.

METHODS AND ANALYSIS

Study design and setting

For the development of our study protocol, we used the reporting guidelines for stepped-wedge cluster randomised trials.[17] We will use a repeated measurement stepped-wedge cluster randomized design. The de-implementation strategy will be introduced in the emergency department of five hospitals (clusters) in the Netherlands (one university and four general teaching hospitals) in six time periods consisting of one or two months, depending on how many patients will be included in the first month. Therefore, the total study duration is six to twelve months (Figure 1). The order in which the participating hospitals will receive the intervention

For peer review only - http://bmjopen.bmj.com/site/about/guidelines.xhtml

will be randomly determined by an independent data manager. Every cluster will start in control condition and by the end of the trial every cluster will have received the intervention. We planned to start our study on 1 May 2020. However, due to the COVID-19 pandemic, the start of our study will be postponed until 1 October 2020.

Patient selection

We will screen all patients (≥ 18 years old) who have urine tests (culture and/or urinalysis) that were obtained during an ED presentation (Figure 2). First, we will exclude all patients that have no positive urinalysis or urine culture, so if both the urinalysis and culture are negative. (Table 1). Further exclusion criteria are patients with a symptomatic UTI (systemic or local symptoms, according to the Centre for Disease Control's (CDC) National Healthcare Safety Network for Urinary Tract Infection) (Table 1),[18] active treatment for a UTI on presentation, patients with an alternate site of infection for which they receive antimicrobial treatment, and patients with ASB and risk factors, defined as pregnant women, patients prior to planned invasive urologic procedures associated with mucosal trauma (including transurethral surgery of the prostate or bladder, ureteroscopy including lithotripsy, and percutaneous stone surgery), and high-risk neutropenia (defined as absolute neutrophil count <500 cells/µl). Patients with altered mental status (AMS) and with possible systemic symptoms of an infection and bacteriuria, since treatment with antimicrobials is then considered appropriate.

Table 1 Definitions of positive urinalysis, urinary tract infection and asymptomatic bacteriuria

Item	Definition
Positive urinalysis[19]	Urinalysis must have one or more of the following results:<br>Dipstick<br>• Abnormal nitrites<br>• Abnormal leukocyte esterase<br>Microscopic analysis:<br>• >5 leukocytes/high-powered field (or reference ranges according to local standard operating procedures)<br>• Abnormal amount of bacteria according local operating procedures.

For peer review only - http://bmjopen.bmj.com/site/about/guidelines.xhtml

Asymptomatic bacteriuria[5]	The presence of one or more species of bacteria growing in the urine at specified quantitative counts ($\geq 10^5$ CFU/ml) regardless of the presence of pyuria, without UTI symptoms or signs.
Urinary tract infection[18]	Patient has at least one of the following signs or symptoms: fever (>38.0°C)†, suprapubic tenderness†, costovertebral angle pain or tenderness†, urinary urgency, urinary frequency, or dysuria* AND patient has a urine culture with no more than two species of organisms identified, at least one of which is a bacterium of ≥10^5 CFU/ml.
Catheter-associated* urinary tract infection[18]	A urinary tract infection where a urinary catheter was in place for >2 days on the date of event, with day of placement being day 1, and was in place on the date of event or the day before.

†With no other recognized cause. *A urinary catheter in situ could cause patient complaints of frequency, urgency, or dysuria. Therefore, these symptoms cannot be used in patients with catheters.

Primary and secondary endpoints

The primary endpoint is the percentage of patients with ASB without risk factors or another alternative site of infections, who are treated with antimicrobials. We define ASB according to the international guideline provided by the Infectious Diseases Society of America (IDSA) (Table 1).[5] Secondary endpoints are the duration of antimicrobial treatment for ASB, the number of urine cultures and urinalysis (dipsticks and microscopic analysis) per 1000 patients at the emergency department, the percentage of asymptomatic patients treated for urine cultures that are considered positive in daily practice; a quantitative count of bacteria $\geq 10^3$ CFU/ml, and the percentage of patients without symptoms or risk factors treated with antimicrobials for a positive urinalysis without an obtained culture. Explorative analyses will evaluate the total number of urine cultures ordered in the hospital and indications for negative urine cultures during the baseline period.

Data collection

The study will collate a list of all urine cultures and urinalyses performed in the emergency department including corresponding microbiology laboratory and clinical chemistry data. All relevant patient information, including the presence of other possible infections, of those with positive cultures will be reviewed from medical and nursing records by a research physician to determine if patients have ASB. For patients with ASB, we will

For peer review only - http://bmjopen.bmj.com/site/about/guidelines.xhtml

collect data on antimicrobial treatment, including antimicrobial prescription and duration of antimicrobial therapy, and the specified bacterial count and pathogen according to the standard operating procedure of the microbiology laboratory. For feasibility reasons, a random sample of a total number of 220 negative urine cultures will be screened by medical record review to evaluate the indication for testing during the baseline period. We will use this data in the further development of an additional local de-implementation strategy based on the assessment of determinants of current practice. Furthermore, we will collect the patient level variables, including patient characteristics (age, gender, residency in nursing home), Charlson comorbidity index,[20] Modified Early Warning Score (MEWS),[21] chronic urinary catheter use, reason for presentation at the emergency department, and use of antimicrobials for uropathogens in the last seven days before presentation at the emergency department. We will also collect the reasons for urine testing documented in the medical record. Data from medical records will be collected in an electronic GCP-compliant database, and we will audit a random sample of 10% to assure the validity of data collection.

De-implementation strategy

Our de-implementation strategy is a tailored multifaceted intervention based on a Dutch de-implementation guide and an international implementation guide to reduce overtreatment of ASB in low-risk patients.[14 22] We will use a combination of interventions, such as education, audit and feedback and organizational adjustments, because these strategies have shown to be successful for reducing treatment of ASB in prior studies.[11 23] The de-implementation strategy can be divided into a general part that will be introduced in every hospital by our project group, and additional local strategies introduced by local healthcare workers, focused on the local setting (Table 2).

For the general strategy, we will start the intervention period by holding one or more educational meetings about overtreatment of ASB for all healthcare workers in the emergency department. During these educational meetings, we will inform all healthcare workers about the current ASB guidelines and discuss the local barriers and facilitators to reduce its overtreatment. Next to this, the results of the baseline period will be used as competitive feedback report between the participating hospitals. This competitive feedback report will be used to raise awareness among healthcare workers.[24] During this meeting we will distribute posters and pocket cards with an algorithm (Figure 3a and 3b) for appropriate indications for urine cultures and urinalysis, and overtreatment of ASB, based on current guidelines.[5 25] The kick-off meeting will be led by the project

For peer review only - http://bmjopen.bmj.com/site/about/guidelines.xhtml

coordinator and the local champion. These tools will be available throughout the intervention period and after the intervention period, if necessary. We will send emails with the anonymous feedback report and the algorithm to healthcare workers who were not able to join the meeting.

For the additional local part of the strategy, a local champion, one of the leading physicians, will be appointed in each participating hospital and will be responsible for the de-implementation strategy. The local champion will start an asymptomatic bacteriuria quality improvement team (ASB-team), which will consist of at least an emergency doctor, infectious disease physician, a resident internal medicine, and a nurse from the emergency department. The ASB-team will be motivated to assess barriers and facilitators within their hospital and to start additional interventions based on the local setting. For example, by educating and/or reminding healthcare workers about appropriate urine testing and treatment for ASB, and evaluating and/or adjusting local protocols to reduce inappropriate urine testing and antimicrobial use. Further, the ASB-team will collaborate with the antibiotic stewardship teams. During the intervention period, the project coordinator will be in contact with the local champion and/or the ASB-team once every two weeks to exchange ideas and experiences based on interim results (ordering patterns of urine culture) and assessment of local facilitators and barriers.

Table 2 Summary of the de-implementation strategy

**Item**	**Description**
**General part**	
Educational meeting with competitive feedback	Combinations of strategies to reduce treatment of ASB.
Clinical decision tools	Pocket cards and posters with algorithms as an aid and reminder for appropriate and inappropriate urine testing and treatment for ASB.
**Local part**	
Local champion	One of the leading physicians who is responsible for the de-implementation strategy.
ASB-team	Consists of a local champion, emergency doctor, infectious disease physician, resident internal medicine, and nurse for the emergency department.

For peer review only - http://bmjopen.bmj.com/site/about/guidelines.xhtml

Additional interventions	Based on local setting. For example, education and/or reminding healthcare workers about appropriate urine testing and treatment of ASB.

Sample size

We will use a stepped-wedge hospital-randomized design with six time periods and five steps, in which one hospital will switch from control to treatment at each step. Previous studies have shown reduction of inappropriate treatment for ASB of approximately 50% following de-implementation strategies.[8] Therefore, we anticipate that the proportion of patients who received inappropriate antimicrobial treatment for ASB will be reduced by 50% from 0.450 to 0.225 after the de-implementation strategy. Assuming an intra-hospital correlation coefficient of 0.10, a total of at least 420 patients (= 14 patients * 6 time periods * 5 hospitals) will achieve 80% power to detect this reduction using the two-sided Wald Z-Test with a significance level of 0.05. We used PASS version 15.0.10 to calculate this sample size. During the same time period, we will include additional patients with positive urinalyses.

Statistical analysis

Effect evaluation

Categorical data were summarised as frequency and percentage, and continuous data as mean (standard deviation) or median (range). We will use fixed effects models to analyse the overall effect of our de-implementation strategy on overtreatment of ASB.[26] To adjust for clustering and temporal trends, we will include the clusters (hospitals) as a categorical predictor and the time in months as a fixed effect in the model. To correct for the Level 2 variance, we will multiply the standard error estimates by the DEFT, which is the square root of the design effect. We will evaluate the differences between baseline period and intervention period according an intention to treat principle. To adjust for confounders, we will include the main risk factors for overtreatment of ASB (namely age, acutely altered mental status, and positive urinalysis)[27] in the model. We will use the Akaike's Information Criterion and ANOVA tables to compare the fitting of the models. We will report the results of the adjusted, unadjusted, and intercept model. A two-sided p value < 0.05 was considered significant.

Process evaluation

For peer review only - http://bmjopen.bmj.com/site/about/guidelines.xhtml

During the planned regular meetings with members of the local ASB teams to report results and feedback, we will evaluate the introduced interventions. By exchanging thoughts and experiences on the implemented interventions, we will be able to determine what intervention(s) will work best in daily practice. For the process evaluation, we will evaluate the de-implementation strategy adherence and compliance. Therefore, all elements of the de-implementation strategy will be explored in all participating hospitals. We will award the hospitals with feedback points for all elements of the de-implementation strategy.[28] For example, points will be given for the number of persons present at the planned meetings and for every strategy that is implemented.

Economic evaluation

We will primarily perform a cost-effectiveness analysis, in which we evaluate the cost effectiveness ratio (CER) (CER = cost of de-implementation / healthcare cost reduction). Our goal is to achieve a reduction in urine culture, urinalysis, and antimicrobial prescriptions, and thereby a reduction in costs. The costs of our de-implementation strategy will be divided in non-recurrent study-related costs and recurrent de-implementation-related costs. Non-recurrent study-related costs include material costs, developmental costs of the de-implementation strategy, and costs of evaluation of the de-implementation. Recurrent costs include the costs to introduce the de-implementation strategy, such as time of the extra meetings of the ASB-team and time invested by the local champion. For feasibility reasons, we will use the number of all urine cultures, urinalyses and antimicrobial prescriptions, representing the main cost benefits due to our strategy. The costs for urine cultures, urinalyses, and antimicrobials used in the analyses will be based on Dutch guideline on healthcare economic evaluations.[29] The result of the cost-effectiveness analysis will be used in a budget impact analysis to assess the financial impact on the hospital's budget to implement and sustain the de-implementation strategy.

ETHICS AND DISSEMINATION

Ethical approval was obtained on 13 December 2019 from Medical Ethics Research Committee of the Academic Medical Centre (Amsterdam, the Netherlands), with a waiver for informed consent. Local feasibility will be obtained by the local institutional scientific review boards of all participated hospitals. This trial is registered at Netherlands Trial Register, trial NL8242, trialregister.nl/trial/8242. All relevant results of our study will be disseminated through publications in peer-reviewed journals and presentations at relevant (scientific) conferences. No identifiable patient data will be disseminated.

For peer review only - http://bmjopen.bmj.com/site/about/guidelines.xhtml

For peer review only

Patient and Public Involvement

This research will be performed without patient involvement. Patients will not be invited to comment on the study design and will not be consulted to develop patient relevant outcomes or interpret the results. Patients will not be invited to contribute to the writing or editing of this document for readability or accuracy.

DISCUSSION

This study protocol describes the design, de-implementation strategy and process evaluation to reduce overtreatment of ASB in the daily practice of emergency medicine. Our de-implementation strategy could prevent the use of unnecessary urine diagnostics and subsequent inappropriate treatment of ASB, improve healthcare quality, lower the increase in antimicrobial resistance, and save costs. If proven to be (cost) effective, this study could provide a nationwide strategy to reduce overtreatment of ASB.

In our study, we will not evaluate the clinical outcome of our strategy, including potential clinical benefits, such as reduced side effects of antibiotics, since previous studies have already shown that ASB in non-risk patients does not lead to an increased risk of symptomatic UTI and that it is safe to not treat ASB.[30 31]

By using a stepped-wedge cluster randomized design, we will be able to compare the outcomes within a cluster between the time intervals in which a cluster received the control and the interventions. Furthermore, our intervention will be enrolled in a systematic manner and eventually be introduced in all participating hospitals. A novel part of our study compared to previous studies that aimed to reduce overtreatment of ASB is that we will be using local champions in each hospital and adjust the intervention based on the local situation.

A limitation of stepped wedge designs is that this require larger sample sizes, as patients' characteristics may be similar within one cluster. By randomization of the start time of the clusters, we intend to balance these important characteristics. However, this cannot be ensured with our relatively small number of clusters.[32] Another possible limitation is that we will not prospectively assess clinical symptoms ourselves, and are limited to documentation of symptomatology in patient files. This might overestimate the number of patients with asymptomatic bacteriuria in the baseline and intervention periods. Since this should be similar in the baseline and intervention periods, we do not expect that this will influence our results. Furthermore, we cannot evaluate

For peer review only

For peer review only - http://bmjopen.bmj.com/site/about/guidelines.xhtml

the impact of an individual intervention. However, we expect that our de-implementation strategy is low cost and well suitable for broad scale implementation.

For peer review only

For peer review only - http://bmjopen.bmj.com/site/about/guidelines.xhtml

REFERENCES

1. The National Institute for Health and Care Excellence (NICE). NICE Do not Do prompts 2013 [Available from: https://www.nice.org.uk/ sharedlearning/nice-do-not-do-prompts accessed 09-09-2019.

2. American Board of Internal Medicine Foundation. Choosing Wisely [Available from: http://www.choosingwisely.org/ accessed 09-09-2019.

3. Nederlandse Federatie van UMC. Doen of laten? [Available from: https://www.doenoflaten.nl/ accessed 09-09-2019.

4. World Health Organization. Global action plan on antimicrobial resistance. Geneva2015 [Available from: https://apps.who.int/iris/bitstream/handle/10665/193736/9789241509763_eng.pdf?sequence=1 accessed 10-10-2019.

5. Nicolle LE, Gupta K, Bradley SF, et al. Clinical Practice Guideline for the Management of Asymptomatic Bacteriuria: 2019 Update by the Infectious Diseases Society of Americaa. *Clinical infectious diseases : an official publication of the Infectious Diseases Society of America* 2019 doi: 10.1093/cid/ciy1121 [published Online First: 2019/03/22]

6. Nicolle LE. Asymptomatic bacteriuria: when to screen and when to treat. *Infectious disease clinics of North America* 2003;17(2):367-94. [published Online First: 2003/07/10]

7. Choosing Wisely: an initiative of the ABIM Foundation. Ten things physicians and patients should question 2013 [Available from: https://www.choosingwisely.org/doctor-patient-lists/american-geriatrics-society/ accessed 09-09-2019.

8. Flokas ME, Andreatos N, Alevizakos M, et al. Inappropriate Management of Asymptomatic Patients With Positive Urine Cultures: A Systematic Review and Meta-analysis. *Open forum infectious diseases* 2017;4(4):ofx207. doi: 10.1093/ofid/ofx207 [published Online First: 2017/12/12]

9. Spivak ES, Burk M, Zhang R, et al. Management of Bacteriuria in Veterans Affairs Hospitals. *Clinical infectious diseases : an official publication of the Infectious Diseases Society of America* 2017;65(6):910-17. doi: 10.1093/cid/cix474 [published Online First: 2017/05/23]

10. van Buul LW, Veenhuizen RB, Achterberg WP, et al. Antibiotic prescribing in Dutch nursing homes: how appropriate is it? *J Am Med Dir Assoc* 2015;16(3):229-37. doi: 10.1016/j.jamda.2014.10.003 [published Online First: 2014/12/03]

11. Loeb M, Brazil K, Lohfeld L, et al. Effect of a multifaceted intervention on number of antimicrobial prescriptions for suspected urinary tract infections in residents of nursing homes: cluster randomised

For peer review only - http://bmjopen.bmj.com/site/about/guidelines.xhtml

controlled trial. *BMJ (Clinical research ed)* 2005;331(7518):669. doi: 10.1136/bmj.38602.586343.55 [published Online First: 2005/09/10]

12. Trautner BW, Grigoryan L, Petersen NJ, et al. Effectiveness of an Antimicrobial Stewardship Approach for Urinary Catheter-Associated Asymptomatic Bacteriuria. *JAMA internal medicine* 2015;175(7):1120-7. doi: 10.1001/jamainternmed.2015.1878 [published Online First: 2015/05/27]
13. Irfan N, Brooks A, Mithoowani S, et al. A Controlled Quasi-Experimental Study of an Educational Intervention to Reduce the Unnecessary Use of Antimicrobials For Asymptomatic Bacteriuria. *PloS one* 2015;10(7):e0132071. doi: 10.1371/journal.pone.0132071 [published Online First: 2015/07/17]
14. Daniel M, Keller S, Mozafarihashjin M, et al. An Implementation Guide to Reducing Overtreatment of Asymptomatic Bacteriuria. *JAMA internal medicine* 2018;178(2):271-76. doi: 10.1001/jamainternmed.2017.7290 [published Online First: 2017/12/12]
15. Caterino JM, Leininger R, Kline DM, et al. Accuracy of Current Diagnostic Criteria for Acute Bacterial Infection in Older Adults in the Emergency Department. *Journal of the American Geriatrics Society* 2017;65(8):1802-09. doi: 10.1111/jgs.14912 [published Online First: 2017/04/26]
16. Gordon LB, Waxman MJ, Ragsdale L, et al. Overtreatment of presumed urinary tract infection in older women presenting to the emergency department. *Journal of the American Geriatrics Society* 2013;61(5):788-92. doi: 10.1111/jgs.12203 [published Online First: 2013/04/18]
17. Hemming K, Haines TP, Chilton PJ, et al. The stepped wedge cluster randomised trial: rationale, design, analysis, and reporting. *BMJ (Clinical research ed)* 2015;350:h391. doi: 10.1136/bmj.h391 [published Online First: 2015/02/11]
18. CDC National Healthcare Safety Network (NHSN). Patient Safety Component Manual 2019 [Available from: https://www.cdc.gov/nhsn/pdfs/pscmanual/pcsmanual_current.pdf accessed 26-09-2019.
19. Deville WL, Yzermans JC, van Duijn NP, et al. The urine dipstick test useful to rule out infections. A meta-analysis of the accuracy. *BMC urology* 2004;4:4. doi: 10.1186/1471-2490-4-4 [published Online First: 2004/06/04]
20. Quan H, Li B, Couris CM, et al. Updating and validating the Charlson comorbidity index and score for risk adjustment in hospital discharge abstracts using data from 6 countries. *American journal of epidemiology* 2011;173(6):676-82. doi: 10.1093/aje/kwq433 [published Online First: 2011/02/19]

For peer review only - http://bmjopen.bmj.com/site/about/guidelines.xhtml

21. Subbe CP, Kruger M, Rutherford P, et al. Validation of a modified Early Warning Score in medical admissions. *QJM* 2001;94(10):521-6. doi: 10.1093/qjmed/94.10.521 [published Online First: 2001/10/06]

22. van Dulmen S, Kool T, Verkerk E. Deïmplementatiegids 2019 [Available from: https://www.doenoflaten.nl/pdf/deïmplementatiegids_jan_2019.pdf accessed 10-10-2019.

23. McMaughan DK, Nwaiwu O, Zhao H, et al. Impact of a decision-making aid for suspected urinary tract infections on antibiotic overuse in nursing homes. *BMC geriatrics* 2016;16:81. doi: 10.1186/s12877-016-0255-9 [published Online First: 2016/04/17]

24. Spoorenberg V, Hulscher ME, Geskus RB, et al. A Cluster-Randomized Trial of Two Strategies to Improve Antibiotic Use for Patients with a Complicated Urinary Tract Infection. *PloS one* 2015;10(12):e0142672. doi: 10.1371/journal.pone.0142672 [published Online First: 2015/12/05]

25. Verenso. Urineweginfecties bij kwetsbare ouderen 2018 [Available from: https://www.verenso.nl/richtlijnen-en-praktijkvoering/richtlijnendatabase/urineweginfecties accessed 11-10-2019.

26. McNeish D, Stapleton LM. Modeling Clustered Data with Very Few Clusters. *Multivariate Behav Res* 2016;51(4):495-518. doi: 10.1080/00273171.2016.1167008 [published Online First: 2016/06/09]

27. Petty LA, Vaughn VM, Flanders SA, et al. Risk Factors and Outcomes Associated With Treatment of Asymptomatic Bacteriuria in Hospitalized Patients. *JAMA internal medicine* 2019 doi: 10.1001/jamainternmed.2019.2871 [published Online First: 2019/08/27]

28. Laan BJ, Maaskant JM, Spijkerman IJB, et al. De-implementation strategy to reduce inappropriate use of intravenous and urinary catheters (RICAT): a multicentre, prospective, interrupted time-series and before and after study. *The Lancet Infectious diseases* 2020 doi: 10.1016/S1473-3099(19)30709-1 [published Online First: 2020/03/11]

29. Hakkaart-van Roijen L, van der Linden N, Bouwmans C, et al. Kostenhandleiding: Methodologie van kostenonderzoek en referentieprijzen voor economische evaluaties in de gezondheidszorg 2016 [Available from: https://www.zorginstituutnederland.nl/publicaties/publicatie/2016/02/29/richtlijn-voor-het-uitvoeren-van-economische-evaluaties-in-de-gezondheidszorg accessed 27-09-2019.

30. Harding GK, Zhanel GG, Nicolle LE, et al. Antimicrobial treatment in diabetic women with asymptomatic bacteriuria. *The New England journal of medicine* 2002;347(20):1576-83. doi: 10.1056/NEJMoa021042 [published Online First: 2002/11/15]

For peer review only - http://bmjopen.bmj.com/site/about/guidelines.xhtml

31. Bengtsson C, Bengtsson U, Bjorkelund C, et al. Bacteriuria in a population sample of women: 24-year follow-up study. Results from the prospective population-based study of women in Gothenburg, Sweden. *Scandinavian journal of urology and nephrology* 1998;32(4):284-9. [published Online First: 1998/10/09]

32. Ellenberg SS. The Stepped-Wedge Clinical Trial: Evaluation by Rolling Deployment. *Jama* 2018;319(6):607-08. doi: 10.1001/jama.2017.21993 [published Online First: 2018/02/17]

For peer review only

AUTHORS' CONTRIBUTIONS

BJL and SEG were involved in the conception of the study and coordinated the funding application. TVH, SEG and BJL drafted the manuscript. BJL outlined the statistical analysis with support of the Clinical Research Unit of the Academic Medical Centre (Amsterdam, the Netherlands). JES, CN, JBS, and CS revised the manuscript critically for important intellectual content. All authors read and approved the final version of the manuscript.

FUNDING STATEMENT

This study is supported by the Netherlands Organisation for Health Research and Development (ZonMw) grant number 839205002. They will have no role in the study design or analysis.

COMPETING INTERESTS STATEMENT

The authors declare that they have no competing interests.

ACKNOWLEDGEMENTS

We thank Vanessa C. Harris (physician Infectious Diseases, Internal Medicine, Amsterdam UMC, University of Amsterdam, Netherlands), for proof-reading and spell checking the manuscript.

FIGURES

Figure 1 The stepped wedge design for de-implementation strategy.

Figure 2 Study profile. ASB=Asymptomatic bacteriuria. UTI=Urinary tract infection.

*Risk factors are pregnancy, patients prior to planned urologic procedures associated with mucosal trauma, and high-risk neutropenia.

Figure 3a Algorithm of indications for urinalysis and cultures in symptomatic patients.

ASB=Asymptomatic bacteriuria. UTI=Urinary tract infection. SWAB=Stichting Werkgroep Antibiotica Beleid (the Dutch Working Party on Antibiotic Policy).

Figure 3b Algorithm of indications for urinalysis and cultures in asymptomatic patients.

ASB=Asymptomatic bacteriuria. UTI=Urinary tract infection. SWAB=Stichting Werkgroep Antibiotica Beleid (the Dutch Working Party on Antibiotic Policy).

For peer review only - http://bmjopen.bmj.com/site/about/guidelines.xhtml

For peer review only

*Fever (>38°C), painful urination, urinary urgency, frequent miction, suprapubic pain, flank pain.

For peer review only

For peer review only - http://bmjopen.bmj.com/site/about/guidelines.xhtml

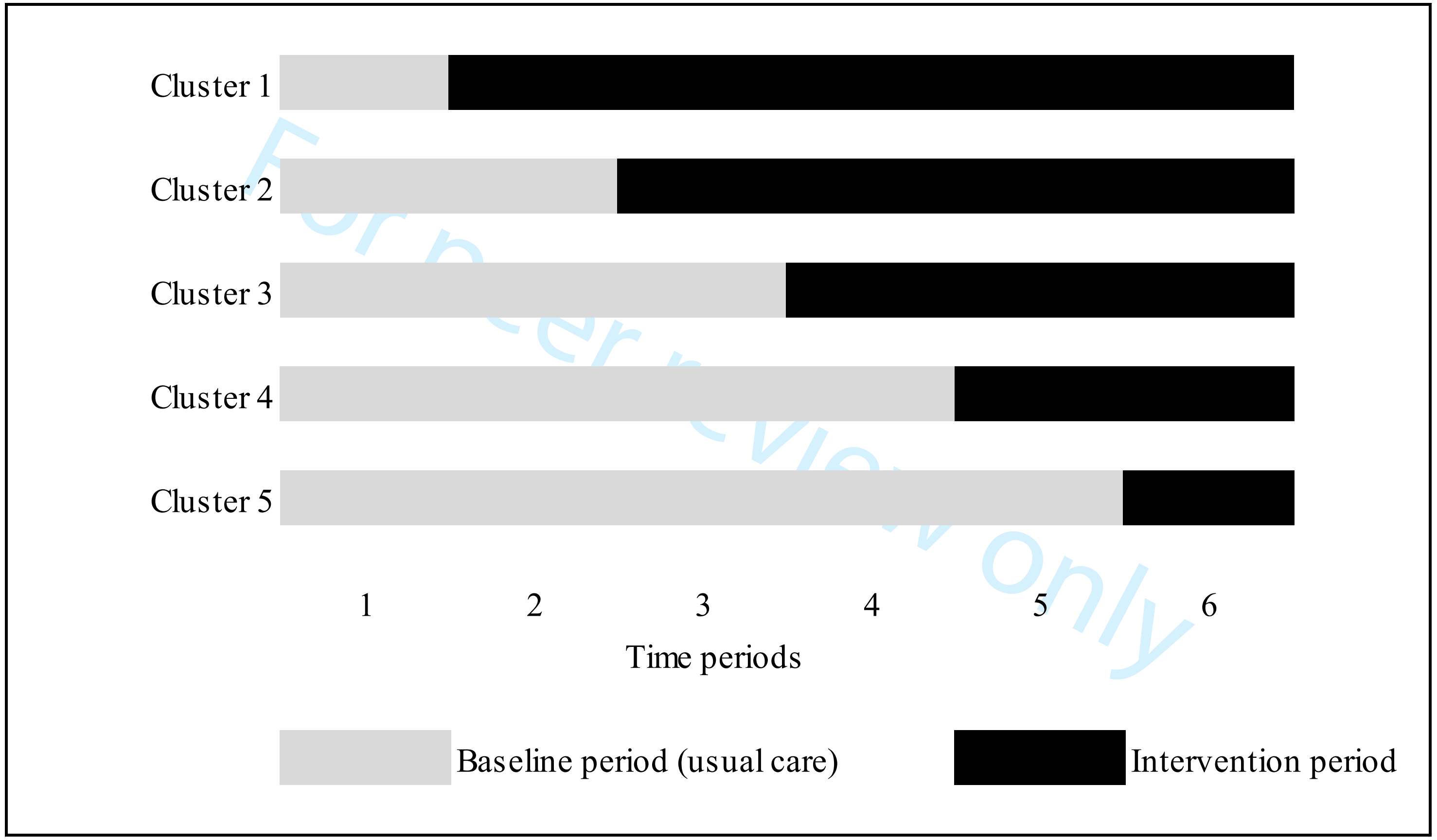
Cluster 1
Cluster 2
Cluster 3
Cluster 4
Cluster 5
1
2
3
4
5
6
Time periods
Baseline period (usual care)
Intervention period

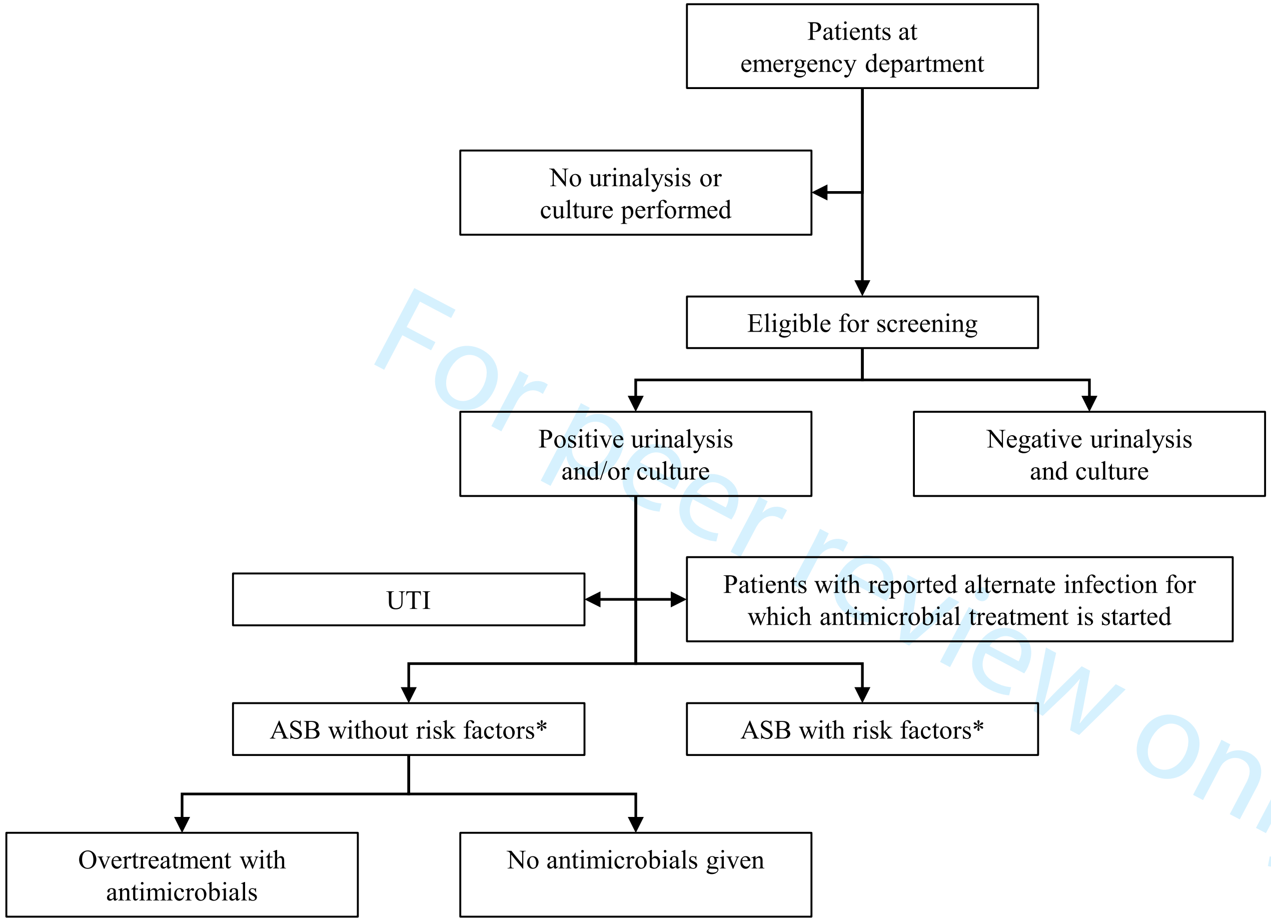

For peer review only - http://bmjopen.bmj.com/site/about/guidelines.xhtml

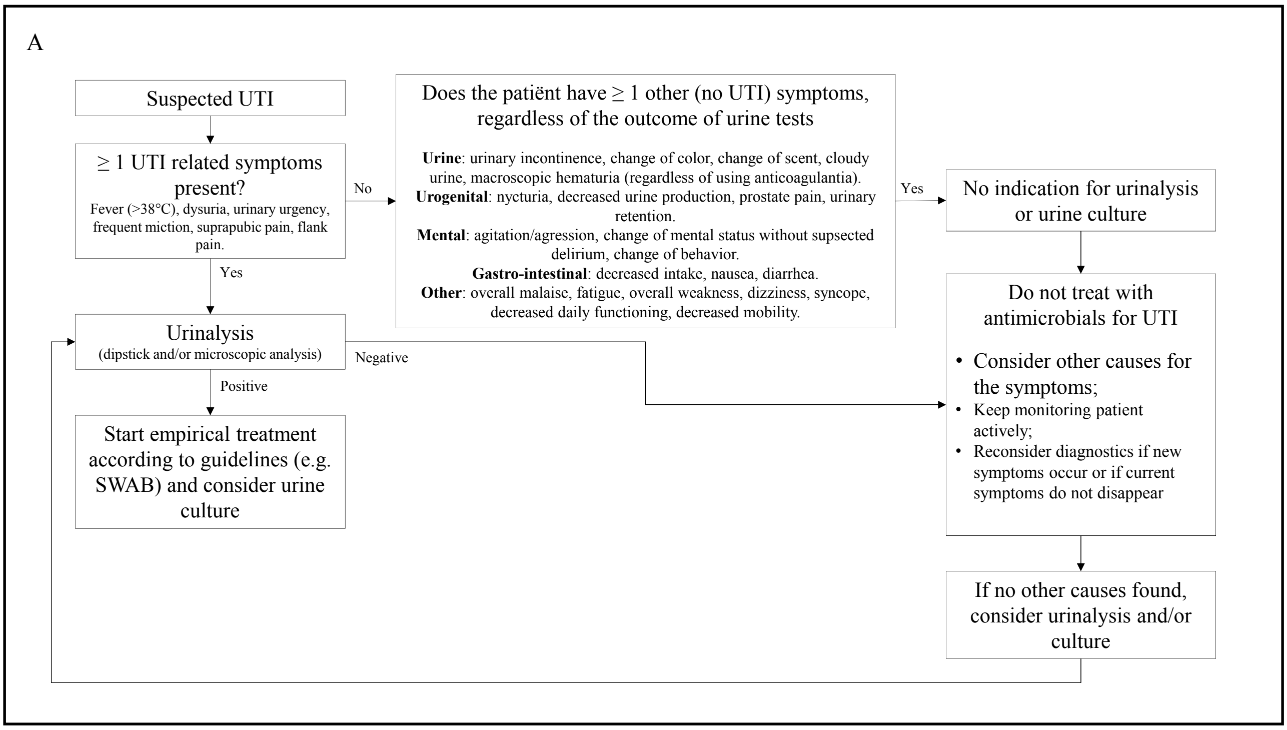
A
Suspected UTI
≥ 1 UTI related symptoms present?
Fever (>38°C), dysuria, urinary urgency, frequent miction, suprapubic pain, flank pain.
No
Yes
Does the patiënt have ≥ 1 other (no UTI) symptoms, regardless of the outcome of urine tests
Urine: urinary incontinence, change of color, change of scent, cloudy urine, macroscopic hematuria (regardless of using anticoagulantia).
Urogenital: nycturia, decreased urine production, prostate pain, urinary retention.
Mental: agitation/agression, change of mental status without supsected delirium, change of behavior.
Gastro-intestinal: decreased intake, nausea, diarrhea.
Other: overall malaise, fatigue, overall weakness, dizziness, syncope, decreased daily functioning, decreased mobility.
Yes
No indication for urinalysis or urine culture
Do not treat with antimicrobials for UTI
• Consider other causes for the symptoms;
• Keep monitoring patient actively;
• Reconsider diagnostics if new symptoms occur or if current symptoms do not disappear
Urinalysis
(dipstick and/or microscopic analysis)
Negative
Positive
Start empirical treatment according to guidelines (e.g. SWAB) and consider urine culture
If no other causes found, consider urinalysis and/or culture

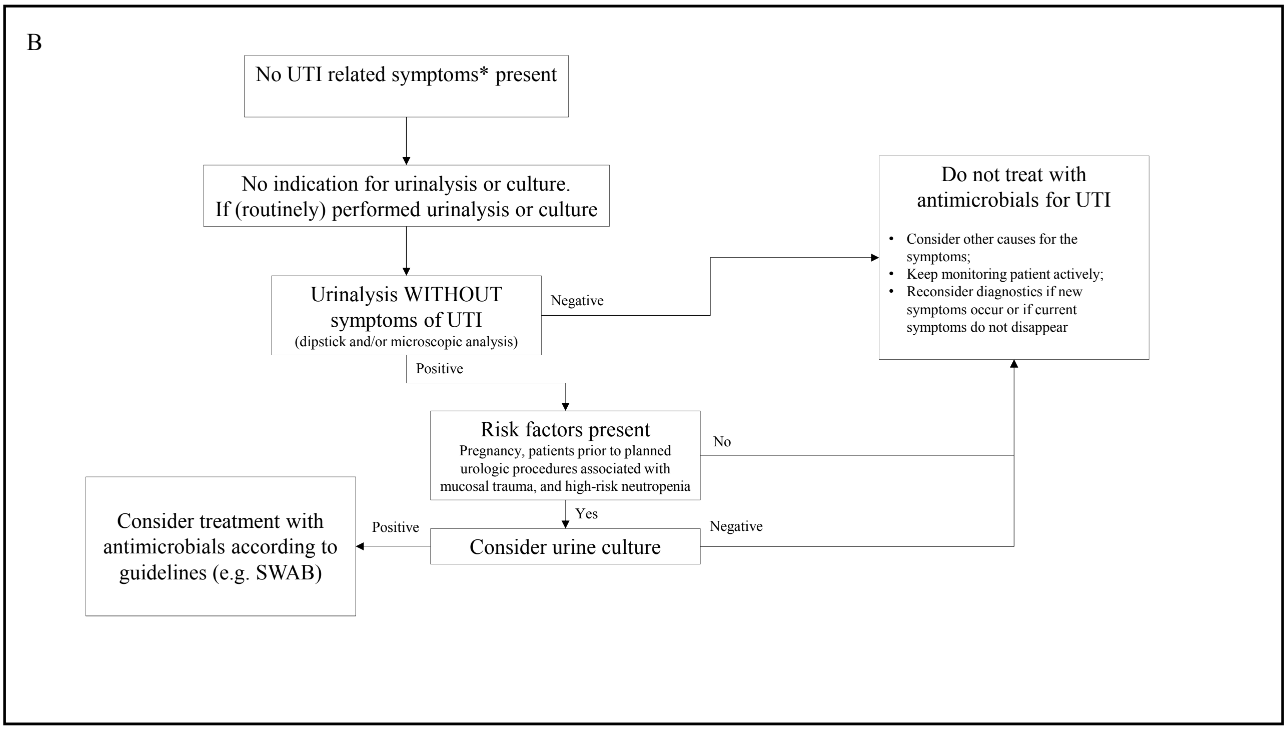
B
No UTI related symptoms* present
No indication for urinalysis or culture.
If (routinely) performed urinalysis or culture
Do not treat with antimicrobials for UTI
• Consider other causes for the symptoms;
• Keep monitoring patient actively;
• Reconsider diagnostics if new symptoms occur or if current symptoms do not disappear
Urinalysis WITHOUT symptoms of UTI
(dipstick and/or microscopic analysis)
Negative
Positive
Risk factors present
Pregnancy, patients prior to planned urologic procedures associated with mucosal trauma, and high-risk neutropenia
No
Yes
Consider treatment with antimicrobials according to guidelines (e.g. SWAB)
Positive
Consider urine culture
Negative

Reporting checklist for protocol of a clinical trial.

Based on the SPIRIT guidelines.

Instructions to authors

Complete this checklist by entering the page numbers from your manuscript where readers will find each of the items listed below.

Your article may not currently address all the items on the checklist. Please modify your text to include the missing information. If you are certain that an item does not apply, please write "n/a" and provide a short explanation.

Upload your completed checklist as an extra file when you submit to a journal.

In your methods section, say that you used the SPIRITreporting guidelines, and cite them as:

Chan A-W, Tetzlaff JM, Altman DG, Laupacis A, Gøtzsche PC, Krleža-Jerić K, Hróbjartsson A, Mann H, Dickersin K, Berlin J, Doré C, Parulekar W, Summerskill W, Groves T, Schulz K, Sox H, Rockhold FW, Rennie D, Moher D. SPIRIT 2013 Statement: Defining standard protocol items for clinical trials. Ann Intern Med. 2013;158(3):200-207

		Reporting Item	Page Number
**Administrative information**			
Title	#1	Descriptive title identifying the study design, population, interventions, and, if applicable, trial acronym	1
Trial registration	#2a	Trial identifier and registry name. If not yet registered, name of intended registry	11
Trial registration: data set	#2b	All items from the World Health Organization Trial Registration Data Set	11
Protocol version	#3	Date and version identifier	1
Funding	#4	Sources and types of financial, material, and other support	17
Roles and responsibilities: contributorship	#5a	Names, affiliations, and roles of protocol contributors	17

For peer review only

Roles and responsibilities: sponsor contact information	#5b	Name and contact information for the trial sponsor	17
Roles and responsibilities: sponsor and funder	#5c	Role of study sponsor and funders, if any, in study design; collection, management, analysis, and interpretation of data; writing of the report; and the decision to submit the report for publication, including whether they will have ultimate authority over any of these activities	17
Roles and responsibilities: committees	#5d	Composition, roles, and responsibilities of the coordinating centre, steering committee, endpoint adjudication committee, data management team, and other individuals or groups overseeing the trial, if applicable (see Item 21a for data monitoring committee)	17
**Introduction**			
Background and rationale	#6a	Description of research question and justification for undertaking the trial, including summary of relevant studies (published and unpublished) examining benefits and harms for each intervention	4
Background and rationale: choice of comparators	#6b	Explanation for choice of comparators	4
Objectives	#7	Specific objectives or hypotheses	4
Trial design	#8	Description of trial design including type of trial (eg, parallel group, crossover, factorial, single group), allocation ratio, and framework (eg, superiority, equivalence, non-inferiority, exploratory)	5
**Methods: Participants, interventions, and outcomes**			
Study setting	#9	Description of study settings (eg, community clinic, academic hospital) and list of countries where data will be collected. Reference to where list of study sites can be obtained	5
Eligibility criteria	#10	Inclusion and exclusion criteria for participants. If applicable, eligibility criteria for study centres and individuals who will	6

For peer review only

		perform the interventions (eg, surgeons, psychotherapists)	
Interventions: description	#11a	Interventions for each group with sufficient detail to allow replication, including how and when they will be administered	8
Interventions: modifications	#11b	Criteria for discontinuing or modifying allocated interventions for a given trial participant (eg, drug dose change in response to harms, participant request, or improving / worsening disease)	8
Interventions: adherance	#11c	Strategies to improve adherence to intervention protocols, and any procedures for monitoring adherence (eg, drug tablet return; laboratory tests)	9
Interventions: concomitant care	#11d	Relevant concomitant care and interventions that are permitted or prohibited during the trial	9
Outcomes	#12	Primary, secondary, and other outcomes, including the specific measurement variable (eg, systolic blood pressure), analysis metric (eg, change from baseline, final value, time to event), method of aggregation (eg, median, proportion), and time point for each outcome. Explanation of the clinical relevance of chosen efficacy and harm outcomes is strongly recommended	7
Participant timeline	#13	Time schedule of enrolment, interventions (including any run-ins and washouts), assessments, and visits for participants. A schematic diagram is highly recommended (see Figure)	5
Sample size	#14	Estimated number of participants needed to achieve study objectives and how it was determined, including clinical and statistical assumptions supporting any sample size calculations	9
Recruitment	#15	Strategies for achieving adequate participant enrolment to reach target sample size	6
**Methods: Assignment of interventions (for controlled trials)**			
Allocation: sequence generation	#16a	Method of generating the allocation sequence (eg, computer-generated random numbers), and list of any factors for stratification. To reduce predictability of a random sequence, details of any planned restriction (eg, blocking) should be provided in a separate document that is unavailable to those who enrol participants or assign interventions	5

For peer review only

Allocation concealment mechanism	#16b	Mechanism of implementing the allocation sequence (eg, central telephone; sequentially numbered, opaque, sealed envelopes), describing any steps to conceal the sequence until interventions are assigned	5
Allocation: implementation	#16c	Who will generate the allocation sequence, who will enrol participants, and who will assign participants to interventions	5
Blinding (masking)	#17a	Who will be blinded after assignment to interventions (eg, trial participants, care providers, outcome assessors, data analysts), and how	n/a
Blinding (masking): emergency unblinding	#17b	If blinded, circumstances under which unblinding is permissible, and procedure for revealing a participant's allocated intervention during the trial	n/a
**Methods: Data collection, management, and analysis**			
Data collection plan	#18a	Plans for assessment and collection of outcome, baseline, and other trial data, including any related processes to promote data quality (eg, duplicate measurements, training of assessors) and a description of study instruments (eg, questionnaires, laboratory tests) along with their reliability and validity, if known. Reference to where data collection forms can be found, if not in the protocol	7
Data collection plan: retention	#18b	Plans to promote participant retention and complete follow-up, including list of any outcome data to be collected for participants who discontinue or deviate from intervention protocols	7
Data management	#19	Plans for data entry, coding, security, and storage, including any related processes to promote data quality (eg, double data entry; range checks for data values). Reference to where details of data management procedures can be found, if not in the protocol	8
Statistics: outcomes	#20a	Statistical methods for analysing primary and secondary outcomes. Reference to where other details of the statistical analysis plan can be found, if not in the protocol	10
Statistics: additional analyses	#20b	Methods for any additional analyses (eg, subgroup and adjusted analyses)	10

For peer review only

Statistics: analysis population and missing data	#20c	Definition of analysis population relating to protocol non-adherence (eg, as randomised analysis), and any statistical methods to handle missing data (eg, multiple imputation)	10
**Methods: Monitoring**			
Data monitoring: formal committee	#21a	Composition of data monitoring committee (DMC); summary of its role and reporting structure; statement of whether it is independent from the sponsor and competing interests; and reference to where further details about its charter can be found, if not in the protocol. Alternatively, an explanation of why a DMC is not needed	n/a
Data monitoring: interim analysis	#21b	Description of any interim analyses and stopping guidelines, including who will have access to these interim results and make the final decision to terminate the trial	n/a
Harms	#22	Plans for collecting, assessing, reporting, and managing solicited and spontaneously reported adverse events and other unintended effects of trial interventions or trial conduct	11
Auditing	#23	Frequency and procedures for auditing trial conduct, if any, and whether the process will be independent from investigators and the sponsor	11
**Ethics and dissemination**			
Research ethics approval	#24	Plans for seeking research ethics committee / institutional review board (REC / IRB) approval	11
Protocol amendments	#25	Plans for communicating important protocol modifications (eg, changes to eligibility criteria, outcomes, analyses) to relevant parties (eg, investigators, REC / IRBs, trial participants, trial registries, journals, regulators)	n/a
Consent or assent	#26a	Who will obtain informed consent or assent from potential trial participants or authorised surrogates, and how (see Item 32)	11
Consent or assent: ancillary studies	#26b	Additional consent provisions for collection and use of participant data and biological specimens in ancillary studies, if applicable	11
Confidentiality	#27	How personal information about potential and enrolled participants will be collected, shared, and maintained in order to protect confidentiality before, during, and after the trial	11

For peer review only

Declaration of interests	#28	Financial and other competing interests for principal investigators for the overall trial and each study site	17
Data access	#29	Statement of who will have access to the final trial dataset, and disclosure of contractual agreements that limit such access for investigators	11
Ancillary and post trial care	#30	Provisions, if any, for ancillary and post-trial care, and for compensation to those who suffer harm from trial participation	n/a
Dissemination policy: trial results	#31a	Plans for investigators and sponsor to communicate trial results to participants, healthcare professionals, the public, and other relevant groups (eg, via publication, reporting in results databases, or other data sharing arrangements), including any publication restrictions	11
Dissemination policy: authorship	#31b	Authorship eligibility guidelines and any intended use of professional writers	11
Dissemination policy: reproducible research	#31c	Plans, if any, for granting public access to the full protocol, participant-level dataset, and statistical code	11
**Appendices**			
Informed consent materials	#32	Model consent form and other related documentation given to participants and authorised surrogates	n/a
Biological specimens	#33	Plans for collection, laboratory evaluation, and storage of biological specimens for genetic or molecular analysis in the current trial and for future use in ancillary studies, if applicable	n/a

The SPIRIT checklist is distributed under the terms of the Creative Commons Attribution License CC-BY-ND 3.0. This checklist was completed on 03. April 2020 using https://www.goodreports.org/, a tool made by the EQUATOR Network in collaboration with Penelope.ai

For peer review only

BMJ Open

De-implementation strategy to reduce overtreatment of asymptomatic bacteriuria: a study protocol for a stepped-wedge cluster randomized trial

Journal:	*BMJ Open*
Manuscript ID	bmjopen-2020-039085.R3
Article Type:	Protocol
Date Submitted by the Author:	04-Nov-2020
Complete List of Authors:	van Horrik, Tessa; Amsterdam UMC - Locatie AMC, Internal Medicine, Infectious Diseases<br>Geerlings, Suzanne; Amsterdam UMC - Locatie AMC, Internal Medicine, Infectious Diseases<br>Stalenhoef, Janneke; OLVG Locatie Oost, Internal Medicine, Infectious Diseases<br>van Nieuwkoop, Cees; HagaZiekenhuis, Internal Medicine, Infectious Diseases<br>Saanen, Joppe; Amsterdam UMC - Locatie AMC, Emergency Medicine<br>Schneeberger, Caroline; Amsterdam UMC - Locatie AMC, Medical Microbiology<br>Laan, Bart; Amsterdam UMC - Locatie AMC, Internal Medicine, Infectious Diseases
**Primary Subject Heading**:	Infectious diseases
Secondary Subject Heading:	Emergency medicine, Diagnostics
Keywords:	Urinary tract infections < UROLOGY, INFECTIOUS DISEASES, Diagnostic microbiology < INFECTIOUS DISEASES, INTERNAL MEDICINE

SCHOLARONE™
Manuscripts

I, the Submitting Author has the right to grant and does grant on behalf of all authors of the Work (as defined in the below author licence), an exclusive licence and/or a non-exclusive licence for contributions from authors who are: i) UK Crown employees; ii) where BMJ has agreed a CC-BY licence shall apply, and/or iii) in accordance with the terms applicable for US Federal Government officers or employees acting as part of their official duties; on a worldwide, perpetual, irrevocable, royalty-free basis to BMJ Publishing Group Ltd ("BMJ") its licensees and where the relevant Journal is co-owned by BMJ to the co-owners of the Journal, to publish the Work in this journal and any other BMJ products and to exploit all rights, as set out in our licence.

The Submitting Author accepts and understands that any supply made under these terms is made by BMJ to the Submitting Author unless you are acting as an employee on behalf of your employer or a postgraduate student of an affiliated institution which is paying any applicable article publishing charge ("APC") for Open Access articles. Where the Submitting Author wishes to make the Work available on an Open Access basis (and intends to pay the relevant APC), the terms of reuse of such Open Access shall be governed by a Creative Commons licence – details of these licences and which Creative Commons licence will apply to this Work are set out in our licence referred to above.

Other than as permitted in any relevant BMJ Author's Self Archiving Policies, I confirm this Work has not been accepted for publication elsewhere, is not being considered for publication elsewhere and does not duplicate material already published. I confirm all authors consent to publication of this Work and authorise the granting of this licence.

For peer review only

TITLE PAGE

Title: De-implementation strategy to reduce overtreatment of asymptomatic bacteriuria: a study protocol for a stepped-wedge cluster randomized trial

Authors: T. M. Z. X. K. van Horrik[1], S. E. Geerlings[1], J. E. Stalenhoef[2], C. van Nieuwkoop[3], J. B. Saanen[4], C. Schneeberger-van der Linden[5], B. J. Laan[1]

[1]Amsterdam UMC, University of Amsterdam, Internal Medicine, Infectious Diseases, Amsterdam, The Netherlands

[2]OLVG, Internal Medicine, Infectious Diseases, Amsterdam, The Netherlands

[3]Haga Teaching Hospital, Internal Medicine, Infectious Diseases, The Hague, The Netherlands

[4]Amsterdam UMC, University of Amsterdam, Emergency Medicine, Amsterdam, The Netherlands

[5]Amsterdam UMC, University of Amsterdam, Medical Microbiology, Amsterdam, The Netherlands

Correspondence to:

Tessa M. Z. X. K. van Horrik, Amsterdam UMC, University of Amsterdam, Internal Medicine, Infectious Diseases, Meibergdreef 9, Amsterdam, Netherlands, +31 20 5665992, t.m.vanhorrik@amsterdamumc.nl

Word count: 3070

For peer review only

For peer review only - http://bmjopen.bmj.com/site/about/guidelines.xhtml

ABSTRACT

Introduction Antimicrobial treatment of asymptomatic bacteriuria (ASB) is one of the most common unnecessary uses of antimicrobials. Earlier studies have shown that the prevalence of this inappropriate treatment ranges from 45 to 83%. Multifaceted interventions based on international guidelines and antimicrobial stewardship can decrease overtreatment of ASB. We have designed a study protocol with the main objective of reducing overtreatment of ASB by 50% through use of a de-implementation strategy.

Methods and analysis We will use a stepped-wedge cluster randomized design, comparing outcomes before and after introduction of our intervention in the emergency department of five hospitals (clusters) in the Netherlands. All patients (≥18 years old) who have a urine test performed in the emergency department will be screened for eligibility. The de-implementation strategy consists of a combination of interventions, including education, audit and feedback. The primary endpoint is overtreatment of ASB in patients without risk factors (e.g. pregnancy, planned invasive urologic procedures and neutropenia). Secondary endpoints are the duration of antimicrobial treatment for ASB, the number of urine cultures and urinalysis per 1000 patients, and overtreatment of positive urinalysis in asymptomatic patients.

Ethics and dissemination Ethical approval was obtained from Medical Ethics Research Committee of the Academic Medical Centre (Amsterdam, the Netherlands) with a waiver for informed consent. Local feasibility will be obtained by the local institutional review boards of all participating hospitals. Our study aims to reduce inappropriate screening and treatment of ASB in emergency departments, improve healthcare quality, lower the increase in antimicrobial resistance, and save costs. If proven (cost)-effective, this study provides a well suited strategy for a nationwide approach to reduce overtreatment of ASB. Relevant results of our study will be disseminated through publications in peer-reviewed journals and presentations at relevant (scientific) conferences.

Registration This trial is registered at Netherlands Trial Register, trial NL8242, trialregister.nl/trial/8242.

For peer review only

For peer review only - http://bmjopen.bmj.com/site/about/guidelines.xhtml

STRENGTHS AND LIMITATIONS OF THIS STUDY

- We will use a stepped-wedge cluster randomized design, ultimately resulting in implementation of our intervention in all participating hospitals.
- Our strategy is well suited for broad-scale de-implementation in emergency departments.
- A possible limitation of this study is that we are not able to evaluate the impact of an individual intervention, since we are using a multifaceted de-implementation strategy.
- Another possible limitation is that we are limited by documentation in the patient files and therefore, we might overestimate the number of patients with asymptomatic bacteriuria.

For peer review only

INTRODUCTION

There is a growing urgency to improve healthcare quality and save costs by implementing practices to reduce unnecessary diagnostics, diagnostic error and related overtreatment, so called low-value care practices. This is exemplified by international campaigns, such as Choosing Wisely and Do not Do prompts, that were launched to reduce unnecessary care in several medical services, resulting in less risks for the patients, saving costs and increasing value for care.[1 2] A parallel campaign initiative, titled 'To Do or Not to Do?' was introduced to de-implement unnecessary care in the Netherlands.[3]

Antimicrobial resistance, as a result of antibiotic overconsumption, is a global threat to public health and there is global consensus about reducing the use of antibiotics.[4] Urinary tract infections (UTI) are one of the most common categories of infections for which antimicrobials are prescribed.[5] However, not all antimicrobial prescriptions are necessary. This is especially true for asymptomatic bacteriuria (ASB), which is the presence of bacteria in the urine of a patient who does not have symptoms of a UTI. ASB is a common finding, especially among women, elderly, and patients with urinary catheters. The prevalence of ASB in adults over 65 years is high, since the prevalence of ASB increases with age.[6] Guidelines strongly recommend not to screen for or treat ASB with antimicrobials, except for specific patients at risk of developing a complicated UTI.[5] This is also outlined in the American Choosing Wisely campaign, which judges treatment of ASB as one of five low-value care services that should be avoided.[7] In 2015, this resulted in the recommendation: "Do not use antimicrobials to treat bacteriuria in older adults unless specific urinary tract symptoms are present".[7]

Regardless, antimicrobials are still frequently used for patients with ASB. A systematic review and meta-analysis from 2017 showed a pooled prevalence of 45% among 4129 ASB cases (95% CI 39-50) who received inappropriate antimicrobial treatment.[8] The prevalence was even higher in a recent retrospective evaluation of 25 hospitals in the United States of America (USA), in which 64% of the 2225 positive urine cultures were classified as ASB and 72% of 961 patients with ASB were treated with antimicrobials.[9] The exact use of antimicrobials for ASB in hospitals in the Netherlands is unknown. However, in 2015, a prospective study performed in ten nursing homes in the Netherlands showed that 115 (32%) of 356 residents with a possible UTI were treated inappropriately, and treatment of ASB was the most common reason.[10] These results indicate that reducing the treatment of ASB is an appropriate target of antimicrobial stewardship strategies.

For peer review only - http://bmjopen.bmj.com/site/about/guidelines.xhtml

Earlier studies demonstrated that multifaceted interventions can effectively reduce overtreatment of ASB in nursing home residents and patients with urinary catheters. In 2005, a cluster randomized trial in 12 nursing homes in Canada and the USA showed a reduction in antimicrobial use after the multifaceted implementation of a diagnostic and treatment algorithm (1.17 versus 1.59 courses of antimicrobials/1000 resident days).[11] For catheterized patients in the USA, a controlled before-after study in 2015 showed a decrease of urine culture ordering from 41.2 to 23.3 per 1000 bed-days.[12] Furthermore, short educational sessions combined with feedback in two academic tertiary acute care hospitals in Canada, performed in 2015, resulted in a reduction of overtreatment for ASB of 8% (2 of 24 patients) in the intervention group compared to 52% (14 of 29 patients) in the control group (OR 0.1; 95% CI 0.02-0.49).[13] In 2018, these findings resulted in the development of an implementation guide to reduce overtreatment of ASB.[14]

However, effectiveness studies after the launch of this implementation guide have not yet been performed in emergency departments (ED). The results of earlier studies suggest that improving ASB management in the emergency department is necessary. In 2017, a prospective observational study performed in the ED of a tertiary care centre in the USA showed that 27 of 71 (38%) older adults were not correctly diagnosed with a UTI.[15] Furthermore, overtreatment of a suspected (but unconfirmed) UTI was very common in a retrospective study performed in the emergency department of another tertiary care centre in the USA in 2013, where 63 of 66 (95%) patients were treated while they had a negative urine culture.[16]

In this study, our aim is to reduce overtreatment of ASB by 50% by using a multifaceted de-implementation strategy based on the implementation guide described above.

METHODS AND ANALYSIS

Study design and setting

For the development of our study protocol, we used the reporting guidelines for stepped-wedge cluster randomised trials.[17] We will use a repeated measurement stepped-wedge cluster randomized design. The de-implementation strategy will be introduced in the emergency department of five hospitals (clusters) in the Netherlands (one university and four general teaching hospitals) in six time periods consisting of one or two months, depending on how many patients will be included in the first month. Therefore, the total study duration is six to twelve months (Figure 1). The order in which the participating hospitals will receive the intervention

For peer review only - http://bmjopen.bmj.com/site/about/guidelines.xhtml

For peer review only

will be randomly determined by an independent data manager. Every cluster will start in control condition and by the end of the trial every cluster will have received the intervention. We planned to start our study on 1 May 2020. However, due to the COVID-19 pandemic, the start of our study will be postponed until 1 October 2020.

Patient selection

We will screen all patients (≥ 18 years old) who have urine tests (culture and/or urinalysis) that were obtained during an ED presentation (Figure 2). First, we will exclude all patients that have no positive urinalysis or urine culture, so if both the urinalysis and culture are negative. (Table 1). Further exclusion criteria are patients with a symptomatic UTI (systemic or local symptoms, according to the Centre for Disease Control's (CDC) National Healthcare Safety Network for Urinary Tract Infection) (Table 1),[18] active treatment for a UTI on presentation, patients with an alternate site of infection for which they receive antimicrobial treatment, and patients with ASB and risk factors, defined as pregnant women, patients prior to planned invasive urologic procedures associated with mucosal trauma (including transurethral surgery of the prostate or bladder, ureteroscopy including lithotripsy, and percutaneous stone surgery), and high-risk neutropenia (defined as absolute neutrophil count <500 cells/µl). Patients with altered mental status (AMS) and with possible systemic symptoms of an infection and bacteriuria, since treatment with antimicrobials is then considered appropriate.

Table 1 Definitions of positive urinalysis, urinary tract infection and asymptomatic bacteriuria

Item	Definition
Positive urinalysis[19]	Urinalysis must have one or more of the following results:<br>Dipstick<br>• Abnormal nitrites<br>• Abnormal leukocyte esterase<br>Microscopic analysis:<br>• >5 leukocytes/high-powered field (or reference ranges according to local standard operating procedures)<br>• Abnormal amount of bacteria according local operating procedures.
Asymptomatic bacteriuria[5]	The presence of one or more species of bacteria growing in the urine at specified quantitative counts (≥10^5 CFU/ml) regardless of the presence

For peer review only - http://bmjopen.bmj.com/site/about/guidelines.xhtml

	of pyuria, without UTI symptoms or signs.
Urinary tract infection[18]	Patient has at least one of the following signs or symptoms: fever (>38.0°C)†, suprapubic tenderness†, costovertebral angle pain or tenderness†, urinary urgency, urinary frequency, or dysuria* AND patient has a urine culture with no more than two species of organisms identified, at least one of which is a bacterium of ≥10^5 CFU/ml.
Catheter-associated* urinary tract infection[18]	A urinary tract infection where a urinary catheter was in place for >2 days on the date of event, with day of placement being day 1, and was in place on the date of event or the day before.

†With no other recognized cause. *A urinary catheter in situ could cause patient complaints of frequency, urgency, or dysuria. Therefore, these symptoms cannot be used in patients with catheters.

Primary and secondary endpoints

The primary endpoint is the percentage of patients with ASB without risk factors or another alternative site of infections, who are treated with antimicrobials. We define ASB according to the international guideline provided by the Infectious Diseases Society of America (IDSA) (Table 1).[5] Secondary endpoints are the duration of antimicrobial treatment for ASB, the number of urine cultures and urinalysis (dipsticks and microscopic analysis) per 1000 patients at the emergency department, the percentage of asymptomatic patients treated for urine cultures that are considered positive in daily practice; a quantitative count of bacteria ≥10^3 CFU/ml, and the percentage of patients without symptoms or risk factors treated with antimicrobials for a positive urinalysis without an obtained culture. Explorative analyses will evaluate the total number of urine cultures ordered in the hospital and indications for negative urine cultures during the baseline period.

Data collection

The study will collate a list of all urine cultures and urinalyses performed in the emergency department including corresponding microbiology laboratory and clinical chemistry data. All relevant patient information, including the presence of other possible infections, of those with positive cultures will be reviewed from medical and nursing records by a research physician to determine if patients have ASB. For patients with ASB, we will collect data on antimicrobial treatment, including antimicrobial prescription and duration of antimicrobial therapy, and the specified bacterial count and pathogen according to the standard operating procedure of the

For peer review only - http://bmjopen.bmj.com/site/about/guidelines.xhtml

microbiology laboratory. For feasibility reasons, a random sample of a total number of 220 negative urine cultures will be screened by medical record review to evaluate the indication for testing during the baseline period. We will use this data in the further development of an additional local de-implementation strategy based on the assessment of determinants of current practice. Furthermore, we will collect the patient level variables, including patient characteristics (age, gender, residency in nursing home), Charlson comorbidity index,[20] Modified Early Warning Score (MEWS),[21] chronic urinary catheter use, reason for presentation at the emergency department, and use of antimicrobials for uropathogens in the last seven days before presentation at the emergency department. We will also collect the reasons for urine testing documented in the medical record. Data from medical records will be collected in an electronic GCP-compliant database, and we will audit a random sample of 10% to assure the validity of data collection.

De-implementation strategy

Our de-implementation strategy is a tailored multifaceted intervention based on a Dutch de-implementation guide and an international implementation guide to reduce overtreatment of ASB in low-risk patients.[14 22] We will use a combination of interventions, such as education, audit and feedback and organizational adjustments, because these strategies have shown to be successful for reducing treatment of ASB in prior studies.[11 23] The de-implementation strategy can be divided into a general part that will be introduced in every hospital by our project group, and additional local strategies introduced by local healthcare workers, focused on the local setting (Table 2).

For the general strategy, we will start the intervention period by holding one or more educational meetings about overtreatment of ASB for all healthcare workers in the emergency department. During these educational meetings, we will inform all healthcare workers about the current ASB guidelines and discuss the local barriers and facilitators to reduce its overtreatment. Next to this, the results of the baseline period will be used as competitive feedback report between the participating hospitals. This competitive feedback report will be used to raise awareness among healthcare workers.[24] During this meeting we will distribute posters and pocket cards with an algorithm (Figure 3a and 3b) for appropriate indications for urine cultures and urinalysis, and overtreatment of ASB, based on current guidelines.[5 25] The kick-off meeting will be led by the project coordinator and the local champion. These tools will be available throughout the intervention period and after the

For peer review only - http://bmjopen.bmj.com/site/about/guidelines.xhtml

intervention period, if necessary. We will send emails with the anonymous feedback report and the algorithm to healthcare workers who were not able to join the meeting.

For the additional local part of the strategy, a local champion, one of the leading physicians, will be appointed in each participating hospital and will be responsible for the de-implementation strategy. The local champion will start an asymptomatic bacteriuria quality improvement team (ASB-team), which will consist of at least an emergency doctor, infectious disease physician, a resident internal medicine, and a nurse from the emergency department. The ASB-team will be motivated to assess barriers and facilitators within their hospital and to start additional interventions based on the local setting. For example, by educating and/or reminding healthcare workers about appropriate urine testing and treatment for ASB, and evaluating and/or adjusting local protocols to reduce inappropriate urine testing and antimicrobial use. Further, the ASB-team will collaborate with the antibiotic stewardship teams. During the intervention period, the project coordinator will be in contact with the local champion and/or the ASB-team once every two weeks to exchange ideas and experiences based on interim results (ordering patterns of urine culture) and assessment of local facilitators and barriers.

Table 2 Summary of the de-implementation strategy

**Item**	**Description**
**General part**	
Educational meeting with competitive feedback	Combinations of strategies to reduce treatment of ASB.
Clinical decision tools	Pocket cards and posters with algorithms as an aid and reminder for appropriate and inappropriate urine testing and treatment for ASB.
**Local part**	
Local champion	One of the leading physicians who is responsible for the de-implementation strategy.
ASB-team	Consists of a local champion, emergency doctor, infectious disease physician, resident internal medicine, and nurse for the emergency department.
Additional interventions	Based on local setting. For example, education and/or reminding healthcare workers about appropriate urine testing and treatment of ASB.

For peer review only - http://bmjopen.bmj.com/site/about/guidelines.xhtml

Sample size

We will use a stepped-wedge hospital-randomized design with six time periods and five steps, in which one hospital will switch from control to treatment at each step. Previous studies have shown reduction of inappropriate treatment for ASB of approximately 50% following de-implementation strategies.[8] Therefore, we anticipate that the proportion of patients who received inappropriate antimicrobial treatment for ASB will be reduced by 50% from 0.450 to 0.225 after the de-implementation strategy. Assuming an intra-hospital correlation coefficient of 0.10, a total of at least 420 patients (= 14 patients * 6 time periods * 5 hospitals) will achieve 80% power to detect this reduction using the two-sided Wald Z-Test with a significance level of 0.05. We used PASS version 15.0.10 to calculate this sample size. During the same time period, we will include additional patients with positive urinalyses.

Statistical analysis

Effect evaluation

Categorical data were summarised as frequency and percentage, and continuous data as mean (standard deviation) or median (range). We will use fixed effects models to analyse the overall effect of our de-implementation strategy on overtreatment of ASB.[26] To adjust for clustering and temporal trends, we will include the clusters (hospitals) as a categorical predictor and the time in months as a fixed effect in the model. To correct for the Level 2 variance, we will multiply the standard error estimates by the DEFT, which is the square root of the design effect. We will evaluate the differences between baseline period and intervention period according an intention to treat principle. To adjust for confounders, we will include the main risk factors for overtreatment of ASB (namely age, acutely altered mental status, and positive urinalysis)[27] in the model. We will use the Akaike's Information Criterion and ANOVA tables to compare the fitting of the models. We will report the results of the adjusted, unadjusted, and intercept model. A two-sided p value < 0.05 was considered significant.

Process evaluation

During the planned regular meetings with members of the local ASB teams to report results and feedback, we will evaluate the introduced interventions. By exchanging thoughts and experiences on the implemented interventions, we will be able to determine what intervention(s) will work best in daily practice. For the process

For peer review only

evaluation, we will evaluate the de-implementation strategy adherence and compliance. Therefore, all elements of the de-implementation strategy will be explored in all participating hospitals. We will award the hospitals with feedback points for all elements of the de-implementation strategy.[28] For example, points will be given for the number of persons present at the planned meetings and for every strategy that is implemented.

Economic evaluation

We will primarily perform a cost-effectiveness analysis, in which we evaluate the cost effectiveness ratio (CER) (CER = cost of de-implementation / healthcare cost reduction). Our goal is to achieve a reduction in urine culture, urinalysis, and antimicrobial prescriptions, and thereby a reduction in costs. The costs of our de-implementation strategy will be divided in non-recurrent study-related costs and recurrent de-implementation-related costs. Non-recurrent study-related costs include material costs, developmental costs of the de-implementation strategy, and costs of evaluation of the de-implementation. Recurrent costs include the costs to introduce the de-implementation strategy, such as time of the extra meetings of the ASB-team and time invested by the local champion. For feasibility reasons, we will use the number of all urine cultures, urinalyses and antimicrobial prescriptions, representing the main cost benefits due to our strategy. The costs for urine cultures, urinalyses, and antimicrobials used in the analyses will be based on Dutch guideline on healthcare economic evaluations.[29] The result of the cost-effectiveness analysis will be used in a budget impact analysis to assess the financial impact on the hospital's budget to implement and sustain the de-implementation strategy.

ETHICS AND DISSEMINATION

Ethical approval was obtained on 13 December 2019 from Medical Ethics Research Committee of the Academic Medical Centre (Amsterdam, the Netherlands), with a waiver for informed consent. Local feasibility will be obtained by the local institutional review boards of all participating hospitals, of which currently all but one have approved. This trial is registered at Netherlands Trial Register, trial NL8242, trialregister.nl/trial/8242. All relevant results of our study will be disseminated through publications in peer-reviewed journals and presentations at relevant (scientific) conferences. No identifiable patient data will be disseminated.

Patient and Public Involvement

For peer review only - http://bmjopen.bmj.com/site/about/guidelines.xhtml

This research will be performed without patient involvement. Patients will not be invited to comment on the study design and will not be consulted to develop patient relevant outcomes or interpret the results. Patients will not be invited to contribute to the writing or editing of this document for readability or accuracy.

Data availability

Data collected from this study, including de-identified individual participant data, will be made available upon publication to investigators whose proposed use of the data has been approved by an independent review committee identified for this purpose. Proposals should be directed to the chief investigator Prof. Dr. Suzanne E. Geerlings (s.e.geerlings@amsterdamumc.nl); to gain access, data requestors will need to sign a data access agreement.

DISCUSSION

This study protocol describes the design, de-implementation strategy and process evaluation to reduce overtreatment of ASB in the daily practice of emergency medicine. Our de-implementation strategy could prevent the use of unnecessary urine diagnostics and subsequent inappropriate treatment of ASB, improve healthcare quality, lower the increase in antimicrobial resistance, and save costs. If proven to be (cost) effective, this study could provide a nationwide strategy to reduce overtreatment of ASB.

In our study, we will not evaluate the clinical outcome of our strategy, including potential clinical benefits, such as reduced side effects of antibiotics, since previous studies have already shown that ASB in non-risk patients does not lead to an increased risk of symptomatic UTI and that it is safe to not treat ASB.[30 31]

By using a stepped-wedge cluster randomized design, we will be able to compare the outcomes within a cluster between the time intervals in which a cluster received the control and the interventions. Furthermore, our intervention will be enrolled in a systematic manner and eventually be introduced in all participating hospitals. A novel part of our study compared to previous studies that aimed to reduce overtreatment of ASB is that we will be using local champions in each hospital and adjust the intervention based on the local situation.

A limitation of stepped wedge designs is that this require larger sample sizes, as patients' characteristics may be similar within one cluster. By randomization of the start time of the clusters, we intend to balance these

For peer review only - http://bmjopen.bmj.com/site/about/guidelines.xhtml

important characteristics. However, this cannot be ensured with our relatively small number of clusters.[32] Another possible limitation is that we will not prospectively assess clinical symptoms ourselves, and are limited to documentation of symptomatology in patient files. This might overestimate the number of patients with asymptomatic bacteriuria in the baseline and intervention periods. Since this should be similar in the baseline and intervention periods, we do not expect that this will influence our results. Furthermore, we cannot evaluate the impact of an individual intervention. However, we expect that our de-implementation strategy is low cost and well suitable for broad scale implementation.

For peer review only

For peer review only - http://bmjopen.bmj.com/site/about/guidelines.xhtml

REFERENCES

1. The National Institute for Health and Care Excellence (NICE). NICE Do not Do prompts 2013 [Available from: https://www.nice.org.uk/ sharedlearning/nice-do-not-do-prompts accessed 09-09-2019.

2. American Board of Internal Medicine Foundation. Choosing Wisely [Available from: http://www.choosingwisely.org/ accessed 09-09-2019.

3. Nederlandse Federatie van UMC. Doen of laten? [Available from: https://www.doenoflaten.nl/ accessed 09-09-2019.

4. World Health Organization. Global action plan on antimicrobial resistance. Geneva2015 [Available from: https://apps.who.int/iris/bitstream/handle/10665/193736/9789241509763_eng.pdf?sequence=1 accessed 10-10-2019.

5. Nicolle LE, Gupta K, Bradley SF, et al. Clinical Practice Guideline for the Management of Asymptomatic Bacteriuria: 2019 Update by the Infectious Diseases Society of Americaa. *Clinical infectious diseases : an official publication of the Infectious Diseases Society of America* 2019 doi: 10.1093/cid/ciy1121 [published Online First: 2019/03/22]

6. Nicolle LE. Asymptomatic bacteriuria: when to screen and when to treat. *Infectious disease clinics of North America* 2003;17(2):367-94. [published Online First: 2003/07/10]

7. Choosing Wisely: an initiative of the ABIM Foundation. Ten things physicians and patients should question 2013 [Available from: https://www.choosingwisely.org/doctor-patient-lists/american-geriatrics-society/ accessed 09-09-2019.

8. Flokas ME, Andreatos N, Alevizakos M, et al. Inappropriate Management of Asymptomatic Patients With Positive Urine Cultures: A Systematic Review and Meta-analysis. *Open forum infectious diseases* 2017;4(4):ofx207. doi: 10.1093/ofid/ofx207 [published Online First: 2017/12/12]

9. Spivak ES, Burk M, Zhang R, et al. Management of Bacteriuria in Veterans Affairs Hospitals. *Clinical infectious diseases : an official publication of the Infectious Diseases Society of America* 2017;65(6):910-17. doi: 10.1093/cid/cix474 [published Online First: 2017/05/23]

10. van Buul LW, Veenhuizen RB, Achterberg WP, et al. Antibiotic prescribing in Dutch nursing homes: how appropriate is it? *J Am Med Dir Assoc* 2015;16(3):229-37. doi: 10.1016/j.jamda.2014.10.003 [published Online First: 2014/12/03]

11. Loeb M, Brazil K, Lohfeld L, et al. Effect of a multifaceted intervention on number of antimicrobial prescriptions for suspected urinary tract infections in residents of nursing homes: cluster randomised

For peer review only - http://bmjopen.bmj.com/site/about/guidelines.xhtml

controlled trial. *BMJ (Clinical research ed)* 2005;331(7518):669. doi: 10.1136/bmj.38602.586343.55 [published Online First: 2005/09/10]

12. Trautner BW, Grigoryan L, Petersen NJ, et al. Effectiveness of an Antimicrobial Stewardship Approach for Urinary Catheter-Associated Asymptomatic Bacteriuria. *JAMA internal medicine* 2015;175(7):1120-7. doi: 10.1001/jamainternmed.2015.1878 [published Online First: 2015/05/27]
13. Irfan N, Brooks A, Mithoowani S, et al. A Controlled Quasi-Experimental Study of an Educational Intervention to Reduce the Unnecessary Use of Antimicrobials For Asymptomatic Bacteriuria. *PloS one* 2015;10(7):e0132071. doi: 10.1371/journal.pone.0132071 [published Online First: 2015/07/17]
14. Daniel M, Keller S, Mozafarihashjin M, et al. An Implementation Guide to Reducing Overtreatment of Asymptomatic Bacteriuria. *JAMA internal medicine* 2018;178(2):271-76. doi: 10.1001/jamainternmed.2017.7290 [published Online First: 2017/12/12]
15. Caterino JM, Leininger R, Kline DM, et al. Accuracy of Current Diagnostic Criteria for Acute Bacterial Infection in Older Adults in the Emergency Department. *Journal of the American Geriatrics Society* 2017;65(8):1802-09. doi: 10.1111/jgs.14912 [published Online First: 2017/04/26]
16. Gordon LB, Waxman MJ, Ragsdale L, et al. Overtreatment of presumed urinary tract infection in older women presenting to the emergency department. *Journal of the American Geriatrics Society* 2013;61(5):788-92. doi: 10.1111/jgs.12203 [published Online First: 2013/04/18]
17. Hemming K, Haines TP, Chilton PJ, et al. The stepped wedge cluster randomised trial: rationale, design, analysis, and reporting. *BMJ (Clinical research ed)* 2015;350:h391. doi: 10.1136/bmj.h391 [published Online First: 2015/02/11]
18. CDC National Healthcare Safety Network (NHSN). Patient Safety Component Manual 2019 [Available from: https://www.cdc.gov/nhsn/pdfs/pscmanual/pcsmanual_current.pdf accessed 26-09-2019.
19. Deville WL, Yzermans JC, van Duijn NP, et al. The urine dipstick test useful to rule out infections. A meta-analysis of the accuracy. *BMC urology* 2004;4:4. doi: 10.1186/1471-2490-4-4 [published Online First: 2004/06/04]
20. Quan H, Li B, Couris CM, et al. Updating and validating the Charlson comorbidity index and score for risk adjustment in hospital discharge abstracts using data from 6 countries. *American journal of epidemiology* 2011;173(6):676-82. doi: 10.1093/aje/kwq433 [published Online First: 2011/02/19]

For peer review only

21. Subbe CP, Kruger M, Rutherford P, et al. Validation of a modified Early Warning Score in medical admissions. *QJM* 2001;94(10):521-6. doi: 10.1093/qjmed/94.10.521 [published Online First: 2001/10/06]

22. van Dulmen S, Kool T, Verkerk E. Deïmplementatiegids 2019 [Available from: https://www.doenoflaten.nl/pdf/deïmplementatiegids_jan_2019.pdf accessed 10-10-2019.

23. McMaughan DK, Nwaiwu O, Zhao H, et al. Impact of a decision-making aid for suspected urinary tract infections on antibiotic overuse in nursing homes. *BMC geriatrics* 2016;16:81. doi: 10.1186/s12877-016-0255-9 [published Online First: 2016/04/17]

24. Spoorenberg V, Hulscher ME, Geskus RB, et al. A Cluster-Randomized Trial of Two Strategies to Improve Antibiotic Use for Patients with a Complicated Urinary Tract Infection. *PloS one* 2015;10(12):e0142672. doi: 10.1371/journal.pone.0142672 [published Online First: 2015/12/05]

25. Verenso. Urineweginfecties bij kwetsbare ouderen 2018 [Available from: https://www.verenso.nl/richtlijnen-en-praktijkvoering/richtlijnendatabase/urineweginfecties accessed 11-10-2019.

26. McNeish D, Stapleton LM. Modeling Clustered Data with Very Few Clusters. *Multivariate Behav Res* 2016;51(4):495-518. doi: 10.1080/00273171.2016.1167008 [published Online First: 2016/06/09]

27. Petty LA, Vaughn VM, Flanders SA, et al. Risk Factors and Outcomes Associated With Treatment of Asymptomatic Bacteriuria in Hospitalized Patients. *JAMA internal medicine* 2019 doi: 10.1001/jamainternmed.2019.2871 [published Online First: 2019/08/27]

28. Laan BJ, Maaskant JM, Spijkerman IJB, et al. De-implementation strategy to reduce inappropriate use of intravenous and urinary catheters (RICAT): a multicentre, prospective, interrupted time-series and before and after study. *The Lancet Infectious diseases* 2020 doi: 10.1016/S1473-3099(19)30709-1 [published Online First: 2020/03/11]

29. Hakkaart-van Roijen L, van der Linden N, Bouwmans C, et al. Kostenhandleiding: Methodologie van kostenonderzoek en referentieprijzen voor economische evaluaties in de gezondheidszorg 2016 [Available from: https://www.zorginstituutnederland.nl/publicaties/publicatie/2016/02/29/richtlijn-voor-het-uitvoeren-van-economische-evaluaties-in-de-gezondheidszorg accessed 27-09-2019.

30. Harding GK, Zhanel GG, Nicolle LE, et al. Antimicrobial treatment in diabetic women with asymptomatic bacteriuria. *The New England journal of medicine* 2002;347(20):1576-83. doi: 10.1056/NEJMoa021042 [published Online First: 2002/11/15]

For peer review only - http://bmjopen.bmj.com/site/about/guidelines.xhtml

31. Bengtsson C, Bengtsson U, Bjorkelund C, et al. Bacteriuria in a population sample of women: 24-year follow-up study. Results from the prospective population-based study of women in Gothenburg, Sweden. *Scandinavian journal of urology and nephrology* 1998;32(4):284-9. [published Online First: 1998/10/09]

32. Ellenberg SS. The Stepped-Wedge Clinical Trial: Evaluation by Rolling Deployment. *Jama* 2018;319(6):607-08. doi: 10.1001/jama.2017.21993 [published Online First: 2018/02/17]

For peer review only

AUTHORS' CONTRIBUTIONS

BJL and SEG were involved in the conception of the study and coordinated the funding application. TVH, SEG and BJL drafted the manuscript. BJL outlined the statistical analysis with support of the Clinical Research Unit of the Academic Medical Centre (Amsterdam, the Netherlands). JES, CN, JBS, and CS revised the manuscript critically for important intellectual content. All authors read and approved the final version of the manuscript.

FUNDING STATEMENT

This study is supported by the Netherlands Organisation for Health Research and Development (ZonMw) grant number 839205002. They will have no role in the study design or analysis.

COMPETING INTERESTS STATEMENT

The authors declare that they have no competing interests.

ACKNOWLEDGEMENTS

We thank Vanessa C. Harris (physician Infectious Diseases, Internal Medicine, Amsterdam UMC, University of Amsterdam, Netherlands), for proof-reading and spell checking the manuscript.

FIGURES

Figure 1 The stepped wedge design for de-implementation strategy.

Figure 2 Study profile. ASB=Asymptomatic bacteriuria. UTI=Urinary tract infection.

*Risk factors are pregnancy, patients prior to planned urologic procedures associated with mucosal trauma, and high-risk neutropenia.

Figure 3a Algorithm of indications for urinalysis and cultures in symptomatic patients.

ASB=Asymptomatic bacteriuria. UTI=Urinary tract infection. SWAB=Stichting Werkgroep Antibiotica Beleid (the Dutch Working Party on Antibiotic Policy).

Figure 3b Algorithm of indications for urinalysis and cultures in asymptomatic patients.

ASB=Asymptomatic bacteriuria. UTI=Urinary tract infection. SWAB=Stichting Werkgroep Antibiotica Beleid (the Dutch Working Party on Antibiotic Policy).

For peer review only - http://bmjopen.bmj.com/site/about/guidelines.xhtml

*Fever (>38°C), painful urination, urinary urgency, frequent miction, suprapubic pain, flank pain.

For peer review only

For peer review only - http://bmjopen.bmj.com/site/about/guidelines.xhtml

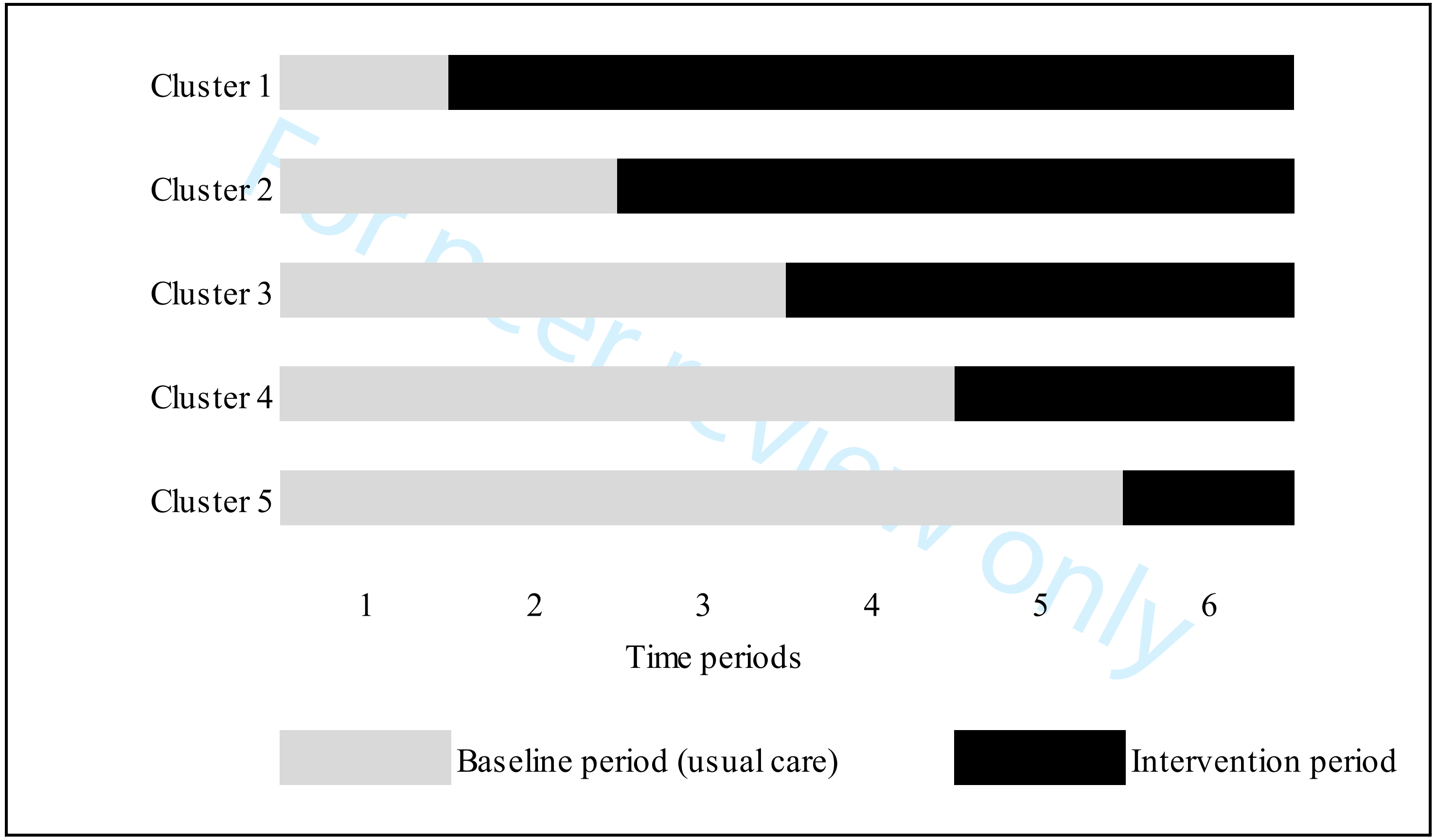

For peer review only

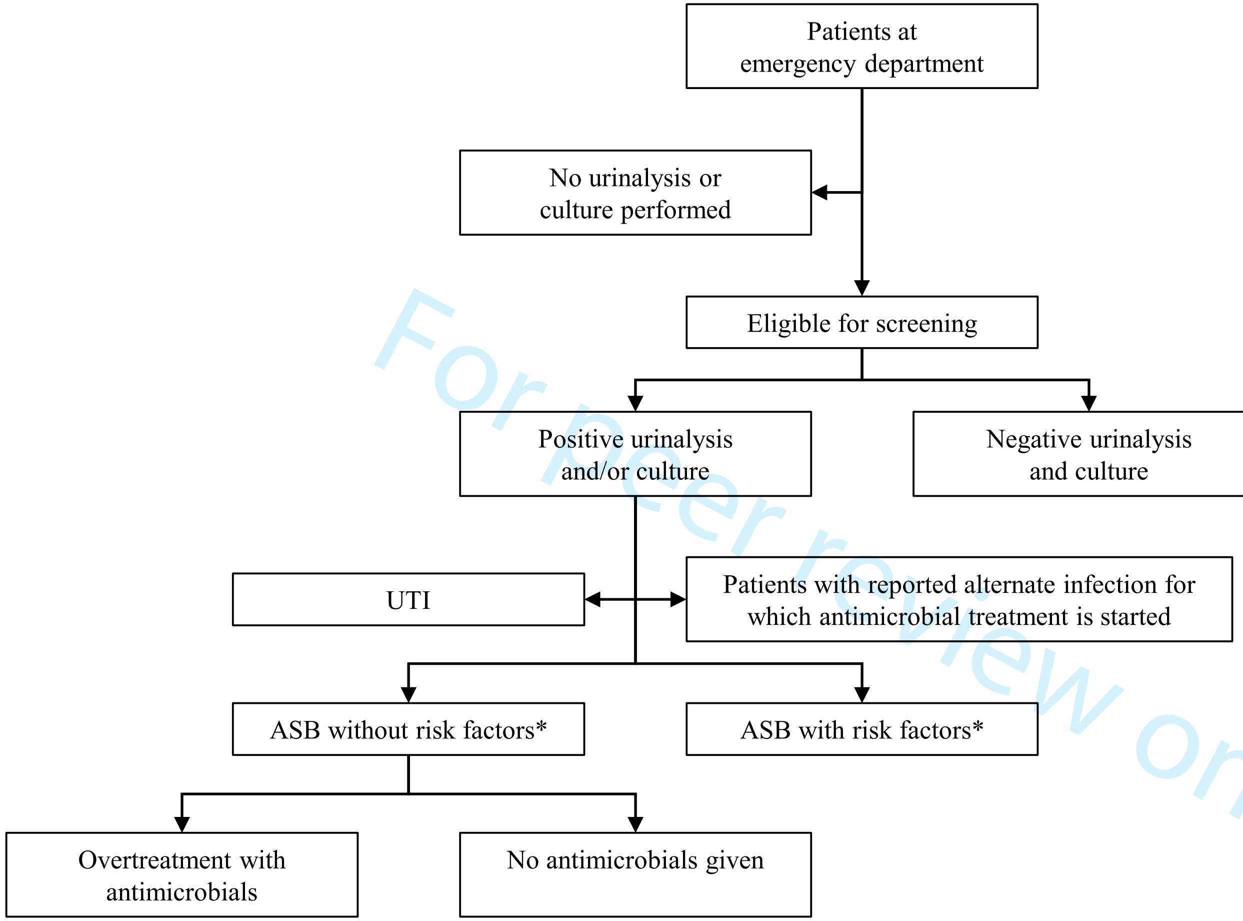

For peer review only

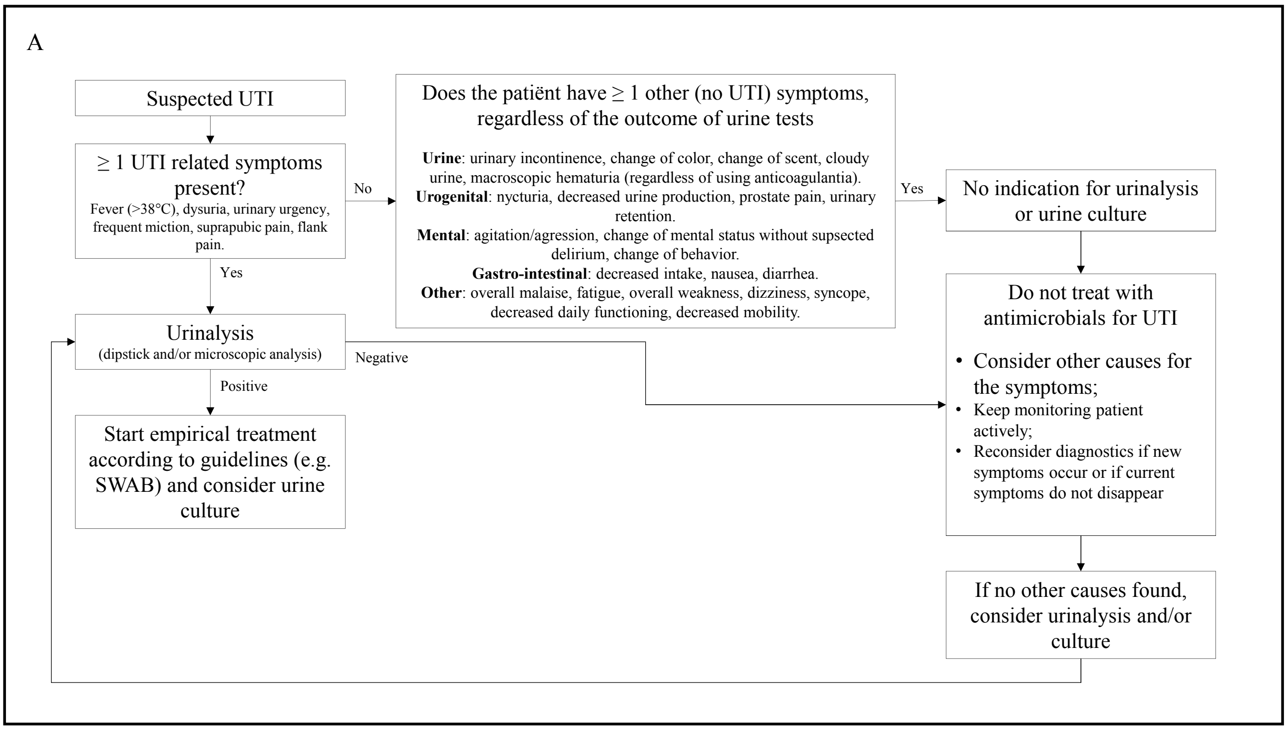
A
Suspected UTI
≥ 1 UTI related symptoms present?
Fever (>38°C), dysuria, urinary urgency, frequent miction, suprapubic pain, flank pain.
No
Yes
Does the patiënt have ≥ 1 other (no UTI) symptoms, regardless of the outcome of urine tests
Urine: urinary incontinence, change of color, change of scent, cloudy urine, macroscopic hematuria (regardless of using anticoagulantia).
Urogenital: nycturia, decreased urine production, prostate pain, urinary retention.
Mental: agitation/agression, change of mental status without supsected delirium, change of behavior.
Gastro-intestinal: decreased intake, nausea, diarrhea.
Other: overall malaise, fatigue, overall weakness, dizziness, syncope, decreased daily functioning, decreased mobility.
Yes
No indication for urinalysis or urine culture
Do not treat with antimicrobials for UTI
• Consider other causes for the symptoms;
• Keep monitoring patient actively;
• Reconsider diagnostics if new symptoms occur or if current symptoms do not disappear
Urinalysis
(dipstick and/or microscopic analysis)
Negative
Positive
Start empirical treatment according to guidelines (e.g. SWAB) and consider urine culture
If no other causes found, consider urinalysis and/or culture

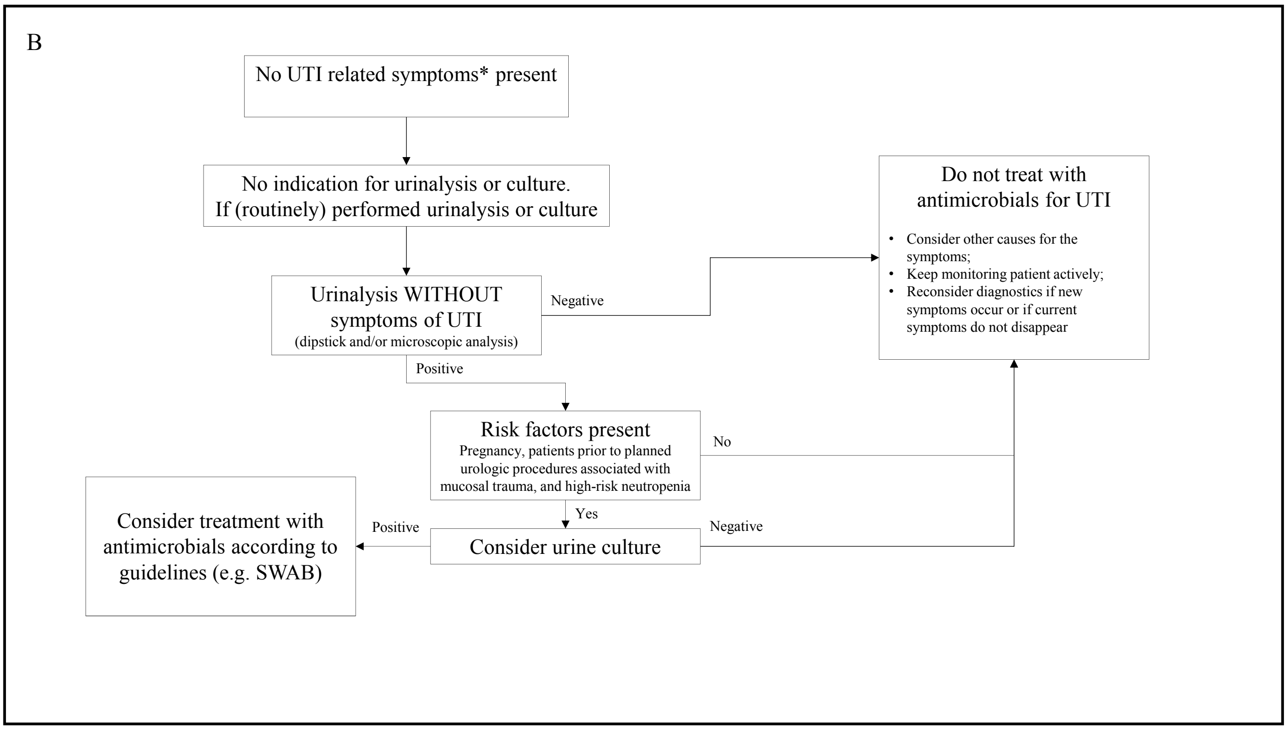
B
No UTI related symptoms* present
No indication for urinalysis or culture.
If (routinely) performed urinalysis or culture
Urinalysis WITHOUT symptoms of UTI
(dipstick and/or microscopic analysis)
Negative
Positive
Do not treat with antimicrobials for UTI
• Consider other causes for the symptoms;
• Keep monitoring patient actively;
• Reconsider diagnostics if new symptoms occur or if current symptoms do not disappear
Risk factors present
Pregnancy, patients prior to planned urologic procedures associated with mucosal trauma, and high-risk neutropenia
No
Yes
Consider urine culture
Positive
Negative
Consider treatment with antimicrobials according to guidelines (e.g. SWAB)

Reporting checklist for protocol of a clinical trial.

Based on the SPIRIT guidelines.

Instructions to authors

Complete this checklist by entering the page numbers from your manuscript where readers will find each of the items listed below.

Your article may not currently address all the items on the checklist. Please modify your text to include the missing information. If you are certain that an item does not apply, please write "n/a" and provide a short explanation.

Upload your completed checklist as an extra file when you submit to a journal.

In your methods section, say that you used the SPIRITreporting guidelines, and cite them as:

Chan A-W, Tetzlaff JM, Altman DG, Laupacis A, Gøtzsche PC, Krleža-Jerić K, Hróbjartsson A, Mann H, Dickersin K, Berlin J, Doré C, Parulekar W, Summerskill W, Groves T, Schulz K, Sox H, Rockhold FW, Rennie D, Moher D. SPIRIT 2013 Statement: Defining standard protocol items for clinical trials. Ann Intern Med. 2013;158(3):200-207

		Reporting Item	Page Number
**Administrative information**			
Title	#1	Descriptive title identifying the study design, population, interventions, and, if applicable, trial acronym	1
Trial registration	#2a	Trial identifier and registry name. If not yet registered, name of intended registry	11
Trial registration: data set	#2b	All items from the World Health Organization Trial Registration Data Set	11
Protocol version	#3	Date and version identifier	1
Funding	#4	Sources and types of financial, material, and other support	17
Roles and responsibilities: contributorship	#5a	Names, affiliations, and roles of protocol contributors	17

For peer review only

Roles and responsibilities: sponsor contact information	#5b	Name and contact information for the trial sponsor	17
Roles and responsibilities: sponsor and funder	#5c	Role of study sponsor and funders, if any, in study design; collection, management, analysis, and interpretation of data; writing of the report; and the decision to submit the report for publication, including whether they will have ultimate authority over any of these activities	17
Roles and responsibilities: committees	#5d	Composition, roles, and responsibilities of the coordinating centre, steering committee, endpoint adjudication committee, data management team, and other individuals or groups overseeing the trial, if applicable (see Item 21a for data monitoring committee)	17
**Introduction**			
Background and rationale	#6a	Description of research question and justification for undertaking the trial, including summary of relevant studies (published and unpublished) examining benefits and harms for each intervention	4
Background and rationale: choice of comparators	#6b	Explanation for choice of comparators	4
Objectives	#7	Specific objectives or hypotheses	4
Trial design	#8	Description of trial design including type of trial (eg, parallel group, crossover, factorial, single group), allocation ratio, and framework (eg, superiority, equivalence, non-inferiority, exploratory)	5
**Methods: Participants, interventions, and outcomes**			
Study setting	#9	Description of study settings (eg, community clinic, academic hospital) and list of countries where data will be collected. Reference to where list of study sites can be obtained	5
Eligibility criteria	#10	Inclusion and exclusion criteria for participants. If applicable, eligibility criteria for study centres and individuals who will	6

For peer review only

		perform the interventions (eg, surgeons, psychotherapists)	
Interventions: description	#11a	Interventions for each group with sufficient detail to allow replication, including how and when they will be administered	8
Interventions: modifications	#11b	Criteria for discontinuing or modifying allocated interventions for a given trial participant (eg, drug dose change in response to harms, participant request, or improving / worsening disease)	8
Interventions: adherance	#11c	Strategies to improve adherence to intervention protocols, and any procedures for monitoring adherence (eg, drug tablet return; laboratory tests)	9
Interventions: concomitant care	#11d	Relevant concomitant care and interventions that are permitted or prohibited during the trial	9
Outcomes	#12	Primary, secondary, and other outcomes, including the specific measurement variable (eg, systolic blood pressure), analysis metric (eg, change from baseline, final value, time to event), method of aggregation (eg, median, proportion), and time point for each outcome. Explanation of the clinical relevance of chosen efficacy and harm outcomes is strongly recommended	7
Participant timeline	#13	Time schedule of enrolment, interventions (including any run-ins and washouts), assessments, and visits for participants. A schematic diagram is highly recommended (see Figure)	5
Sample size	#14	Estimated number of participants needed to achieve study objectives and how it was determined, including clinical and statistical assumptions supporting any sample size calculations	9
Recruitment	#15	Strategies for achieving adequate participant enrolment to reach target sample size	6
**Methods: Assignment of interventions (for controlled trials)**			
Allocation: sequence generation	#16a	Method of generating the allocation sequence (eg, computer-generated random numbers), and list of any factors for stratification. To reduce predictability of a random sequence, details of any planned restriction (eg, blocking) should be provided in a separate document that is unavailable to those who enrol participants or assign interventions	5

For peer review only

Allocation concealment mechanism	#16b	Mechanism of implementing the allocation sequence (eg, central telephone; sequentially numbered, opaque, sealed envelopes), describing any steps to conceal the sequence until interventions are assigned	5
Allocation: implementation	#16c	Who will generate the allocation sequence, who will enrol participants, and who will assign participants to interventions	5
Blinding (masking)	#17a	Who will be blinded after assignment to interventions (eg, trial participants, care providers, outcome assessors, data analysts), and how	n/a
Blinding (masking): emergency unblinding	#17b	If blinded, circumstances under which unblinding is permissible, and procedure for revealing a participant's allocated intervention during the trial	n/a
**Methods: Data collection, management, and analysis**			
Data collection plan	#18a	Plans for assessment and collection of outcome, baseline, and other trial data, including any related processes to promote data quality (eg, duplicate measurements, training of assessors) and a description of study instruments (eg, questionnaires, laboratory tests) along with their reliability and validity, if known. Reference to where data collection forms can be found, if not in the protocol	7
Data collection plan: retention	#18b	Plans to promote participant retention and complete follow-up, including list of any outcome data to be collected for participants who discontinue or deviate from intervention protocols	7
Data management	#19	Plans for data entry, coding, security, and storage, including any related processes to promote data quality (eg, double data entry; range checks for data values). Reference to where details of data management procedures can be found, if not in the protocol	8
Statistics: outcomes	#20a	Statistical methods for analysing primary and secondary outcomes. Reference to where other details of the statistical analysis plan can be found, if not in the protocol	10
Statistics: additional analyses	#20b	Methods for any additional analyses (eg, subgroup and adjusted analyses)	10

For peer review only

Statistics: analysis population and missing data	#20c	Definition of analysis population relating to protocol non-adherence (eg, as randomised analysis), and any statistical methods to handle missing data (eg, multiple imputation)	10
**Methods: Monitoring**			
Data monitoring: formal committee	#21a	Composition of data monitoring committee (DMC); summary of its role and reporting structure; statement of whether it is independent from the sponsor and competing interests; and reference to where further details about its charter can be found, if not in the protocol. Alternatively, an explanation of why a DMC is not needed	n/a
Data monitoring: interim analysis	#21b	Description of any interim analyses and stopping guidelines, including who will have access to these interim results and make the final decision to terminate the trial	n/a
Harms	#22	Plans for collecting, assessing, reporting, and managing solicited and spontaneously reported adverse events and other unintended effects of trial interventions or trial conduct	11
Auditing	#23	Frequency and procedures for auditing trial conduct, if any, and whether the process will be independent from investigators and the sponsor	11
**Ethics and dissemination**			
Research ethics approval	#24	Plans for seeking research ethics committee / institutional review board (REC / IRB) approval	11
Protocol amendments	#25	Plans for communicating important protocol modifications (eg, changes to eligibility criteria, outcomes, analyses) to relevant parties (eg, investigators, REC / IRBs, trial participants, trial registries, journals, regulators)	n/a
Consent or assent	#26a	Who will obtain informed consent or assent from potential trial participants or authorised surrogates, and how (see Item 32)	11
Consent or assent: ancillary studies	#26b	Additional consent provisions for collection and use of participant data and biological specimens in ancillary studies, if applicable	11
Confidentiality	#27	How personal information about potential and enrolled participants will be collected, shared, and maintained in order to protect confidentiality before, during, and after the trial	11

For peer review only

Declaration of interests	#28	Financial and other competing interests for principal investigators for the overall trial and each study site	17
Data access	#29	Statement of who will have access to the final trial dataset, and disclosure of contractual agreements that limit such access for investigators	11
Ancillary and post trial care	#30	Provisions, if any, for ancillary and post-trial care, and for compensation to those who suffer harm from trial participation	n/a
Dissemination policy: trial results	#31a	Plans for investigators and sponsor to communicate trial results to participants, healthcare professionals, the public, and other relevant groups (eg, via publication, reporting in results databases, or other data sharing arrangements), including any publication restrictions	11
Dissemination policy: authorship	#31b	Authorship eligibility guidelines and any intended use of professional writers	11
Dissemination policy: reproducible research	#31c	Plans, if any, for granting public access to the full protocol, participant-level dataset, and statistical code	11
**Appendices**			
Informed consent materials	#32	Model consent form and other related documentation given to participants and authorised surrogates	n/a
Biological specimens	#33	Plans for collection, laboratory evaluation, and storage of biological specimens for genetic or molecular analysis in the current trial and for future use in ancillary studies, if applicable	n/a

The SPIRIT checklist is distributed under the terms of the Creative Commons Attribution License CC-BY-ND 3.0. This checklist was completed on 03. April 2020 using https://www.goodreports.org/, a tool made by the EQUATOR Network in collaboration with Penelope.ai

For peer review only
